# Pangenome comparison of *Bacteroides fragilis* genomospecies unveils genetic diversity and ecological insights

**DOI:** 10.1128/msystems.00516-24

**Published:** 2024-06-27

**Authors:** Renee E. Oles, Marvic Carrillo Terrazas, Luke R. Loomis, Chia-Yun Hsu, Caitlin Tribelhorn, Pedro Belda-Ferre, Allison C. Ea, MacKenzie Bryant, Jocelyn A. Young, Hannah C. Carrow, William J. Sandborn, Parambir S. Dulai, Mamata Sivagnanam, David Pride, Rob Knight, Hiutung Chu

**Affiliations:** 1 Department of Pathology, University of California, San Diego, California, USA; 2 Department of Pediatrics, School of Medicine, University of California, San Diego, California, USA; 3 Rady Children’s Hospital, San Diego, California, USA; 4 Division of Gastroenterology, University of California, San Diego, California, USA; 5 Center for Microbiome Innovation, University of California, San Diego, California, USA; 6 Division of Gastroenterology, Northwestern University, Chicago, Illinois, USA; 7 Center for Innovative Phage Applications and Therapeutics (IPATH), University of California, San Diego, California, USA; 8 Center of Advanced Laboratory Medicine (CALM), University of California, San Diego, California, USA; 9 Shu Chien-Gene Lay Department of Bioengineering, University of California, San Diego, California, USA; 10 Department of Computer Science & Engineering, University of California, San Diego, California, USA; 11 Halıcıoğlu Data Science Institute, University of California, San Diego, California, USA; 12 Chiba University-UC San Diego Center for Mucosal Immunology, Allergy and Vaccines (cMAV), University of California, San Diego, California, USA; Institute for Systems Biology, Seattle, Washington, USA

**Keywords:** pangenome, commensal bacteria, genomic diversity, niche adaptation, *Bacteroides*

## Abstract

*Bacteroides fragilis* is a Gram-negative commensal bacterium commonly found in the human colon, which differentiates into two genomospecies termed divisions I and II. Through a comprehensive collection of 694 *B. fragilis* whole genome sequences, we identify novel features distinguishing these divisions. Our study reveals a distinct geographic distribution with division I strains predominantly found in North America and division II strains in Asia. Additionally, division II strains are more frequently associated with bloodstream infections, suggesting a distinct pathogenic potential. We report differences between the two divisions in gene abundance related to metabolism, virulence, stress response, and colonization strategies. Notably, division II strains harbor more antimicrobial resistance (AMR) genes than division I strains. These findings offer new insights into the functional roles of division I and II strains, indicating specialized niches within the intestine and potential pathogenic roles in extraintestinal sites.

**IMPORTANCE:**

Understanding the distinct functions of microbial species in the gut microbiome is crucial for deciphering their impact on human health. Classifying division II strains as *Bacteroides fragilis* can lead to erroneous associations, as researchers may mistakenly attribute characteristics observed in division II strains to the more extensively studied division I *B. fragilis*. Our findings underscore the necessity of recognizing these divisions as separate species with distinct functions. We unveil new findings of differential gene prevalence between division I and II strains in genes associated with intestinal colonization and survival strategies, potentially influencing their role as gut commensals and their pathogenicity in extraintestinal sites. Despite the significant niche overlap and colonization patterns between these groups, our study highlights the complex dynamics that govern strain distribution and behavior, emphasizing the need for a nuanced understanding of these microorganisms.

## OBSERVATION

*Bacteroides fragilis* is a persistent colonizer of the human gut linked to both health and disease (1) and is composed of two genomospecies termed divisions I and II. They have primarily been differentiated through the presence of *cepA*, a beta-lactamase, which is unique to division I (2), and the chromosomally encoded carbapenemase gene (*cfiA* or *ccrA*), which is unique to division II and provides resistance to beta-lactamase inhibitors (3, 4). Due to their genetic similarity, traditional methods such as 16S rRNA gene analysis cannot distinguish between these divisions, yet they share an average nucleotide identity (ANI) of 87%, below the typical species cutoff of 96% (3, 5–10). Here, we conduct a comprehensive genomic comparison and identified genes conserved within each *B. fragilis* division, but not shared between them, shedding light on the unique biological roles and functions of these divisions within their ecological niches.

**Fig 1:**
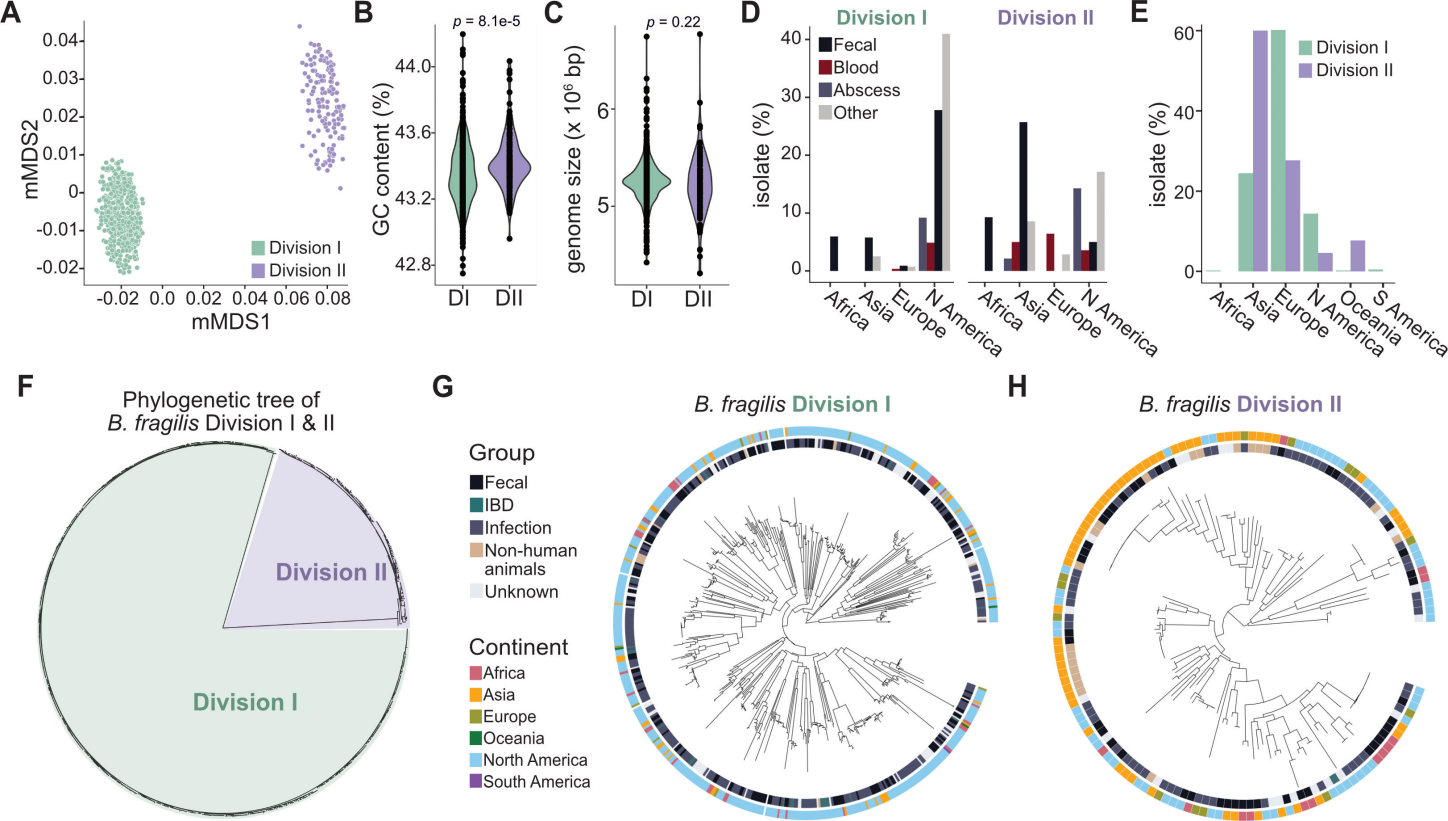
*B. fragilis* is composed of two monophyletic divisions. (**A**) Metric multidimensional scaling (mMDS) of the k-mer based Mash distances of 694 strains, colored by divisions I (green, *n* = 554) and II (purple, *n* = 140). (B) GC content (%) of isolate assemblies in division I and II isolates. Average for division I = 43.35% ± 0.19 and division II = 43.42% ± 0.16 (*P* = 8.1e-5, Welch’s *t*-test with unequal variance; *n* = 694). (C) Genome size (bp) of isolate assemblies in division I and II isolates. Average for division I = 5.26×10^6^ bp and division II = 5.22×10^6^ bp (*P* = 0.22, Welch’s *t*-test with unequal variance; *n* = 694). (D) The proportion of isolates originating from abscess (*P* = 0.18), blood (*P* = 0.0049), and fecal (*P* = 0.0011) samples in division I compared with division II, *P*-values from Fisher’s exact test. Division I: total = 554, fecal = 228, blood = 30, abscess = 51; division II: total = 140, fecal = 56, blood = 21, abscess = 23. The proportion of isolates originating from Africa (*P* = 0.18), Asia (*P* = 2.2e-16), Europe (*P* = 0.00019), or North America (*P* = 2.2e-16) in division I compared with division II, *P*-values from Fisher’s Exact Test. Division I: total = 554, Africa = 33, Asia = 46, Europe = 11, North America = 459; division II: total = 140, Africa = 13, Asia = 58, Europe = 13, North America = 56. (E) Distribution of isolates in each continent per division in the Pasoli et al., 2019 data set. The proportion of isolates originating from Africa (*P* = 1), Asia (*P* = 3.9e-08), Europe (*P* = 9.4e-07), North America (*P* = 0.029), Oceania (*P* = 0.00017), or South America (*P* = 1) in division I (green) compared with division II (purple), *P*-values from Fisher’s exact test. Division I: *n* = 437, Africa = 1, Asia = 107, Europe = 263, North America = 63, Oceania = 1, South America = 2; Division II: *n* = 65, Africa = 0, Asia = 39, Europe = 18, North America = 3, Oceania = 5, South America = 0. (F) Phylogenetic tree of the core genome alignment of 694 strains through maximum likelihood, midpoint rooted, colored by divisions I (green) and II (purple). (G) The phylogenetic tree of the core genome alignment of division I strains through maximum likelihood, midpoint rooted, annotated with the inner ring, Group: healthy, infection, IBD, non-human animal, unknown; and outer ring, Continent: Asia, Africa, Europe, Oceania, North America, and South America (*n* = 554).(H) The phylogenetic tree of the core genome alignment of division II strains through maximum likelihood, midpoint rooted, annotated with the inner ring, Group: healthy, infection, IBD, non-human animal, unknown; and outer ring, Continent: Asia, Africa, Europe, Oceania, North America, and South America (*n* = 140).

We analyze 694 *B. fragilis* whole genome sequences, including 139 from our own collection, which we isolated and sequenced for the first time, and the remaining from public sources (Tables S1 and S2). To compare the genetic relatedness between divisions, we employed Mash, a whole genome k-mer-based approach (11) to determine the genetic distance between each strain (Fig. 1A). Metric multidimensional scaling (mMDS) reveals a clear separation of strains into two distinct divisions (Fig. 1A). To further support this distinction, we discovered a significant difference in GC content between the divisions (Welch’s *t*-test, *P* = 8.1e-5; Cohen’s effect size, d = 0.35) (Fig. 1B)**,** although no differences were found in genome size (Welch’s *t*-test, *P* = 0.22) (Fig. 1C). We also observe a difference in the average GC content in the core genes (present in >99% of isolates) of divisions I (44.6% ± 4.1) and II (45.0% ± 4.0), demonstrating the same trend where division II strains have a moderately higher average GC content than division I (Welch’s *t*-test, *P* = 8.3e-21). Of the shared core genes in division I versus II, the average GC content per gene in divisions I and II is 44.9% ± 3.7 and 45.0% ± 3.8, respectively (Welch’s *t*-test, *P* = 0.0081). However, core genes exclusive to division I have an average GC content of 43.0% ± 5.5, whereas those unique to division II are 44.2% ± 5.7 (Welch’s *t*-test, *P* = 4.2e-19), suggesting the differences in GC content stem from recent evolution between divisions. Although significant, the GC content difference is subtle and may not accurately categorize any given isolate as either division I or II. Finally, based on the maximum likelihood, midpoint-rooted phylogeny of the core genome alignment, divisions I and II separate into discrete clades (Fig. 1F).

We next investigated whether divisions I and II are associated with disease states, isolation sites, or other metadata categories. Division I strains are more prevalent (80% of the total; 554 of 694) than division II. Among the 409 isolates from abscesses, fecal samples, or blood, division I strains are more commonly isolated from fecal samples (74%) compared with division II (56%, Fisher’s exact test, *P* = 0.0011) (Fig. 1D). Conversely, division II strains are more frequently associated with abscesses (23%) or blood (21%) compared with division I strains (16% from abscess, Fisher’s exact test, *P* = 0.18, and 10% from blood, Fisher’s exact test, *P* = 0.0049) (Fig. 1D). Notably, division I and II strains exhibit variations in the continent of isolation. Moreover, 84% (*n* = 459) of division I strains originate from North America, compared with only 40% (*n* = 56) of division II strains (Fisher’s exact test, *P* = 2.2e-16) (Fig. 1D and H). In contrast, only 8% of division I strains originate from Asia (*n* = 46), compared with 41% (*n* = 58) of division II strains (Fisher’s exact test, *P* = 2.2e-16) (Fig. 1D and G). To further explore the geographical distribution of these divisions, we examined 502 species-genome bins (SGBs) classified as *B. fragilis*, which were reconstructed from 9,428 human gut metagenomic samples worldwide (12). 87% and 13% of strains belong to divisions I and II, respectively. No host harbor both divisions, in line with reports from other studies (13–16). Most of the division I strains (75%) originate from Europe or North America, whereas most division II strains (60%) are from Asia (Fig. 1E). This aligns with previous reports indicating a higher rate of *cfiA*+isolates (division II) in Japan, Hong Kong, and India (17). This geographic disparity suggests the under-representation of division II strains in public databases may be due to the limited sampling of specific populations (18).

**Fig 2:**
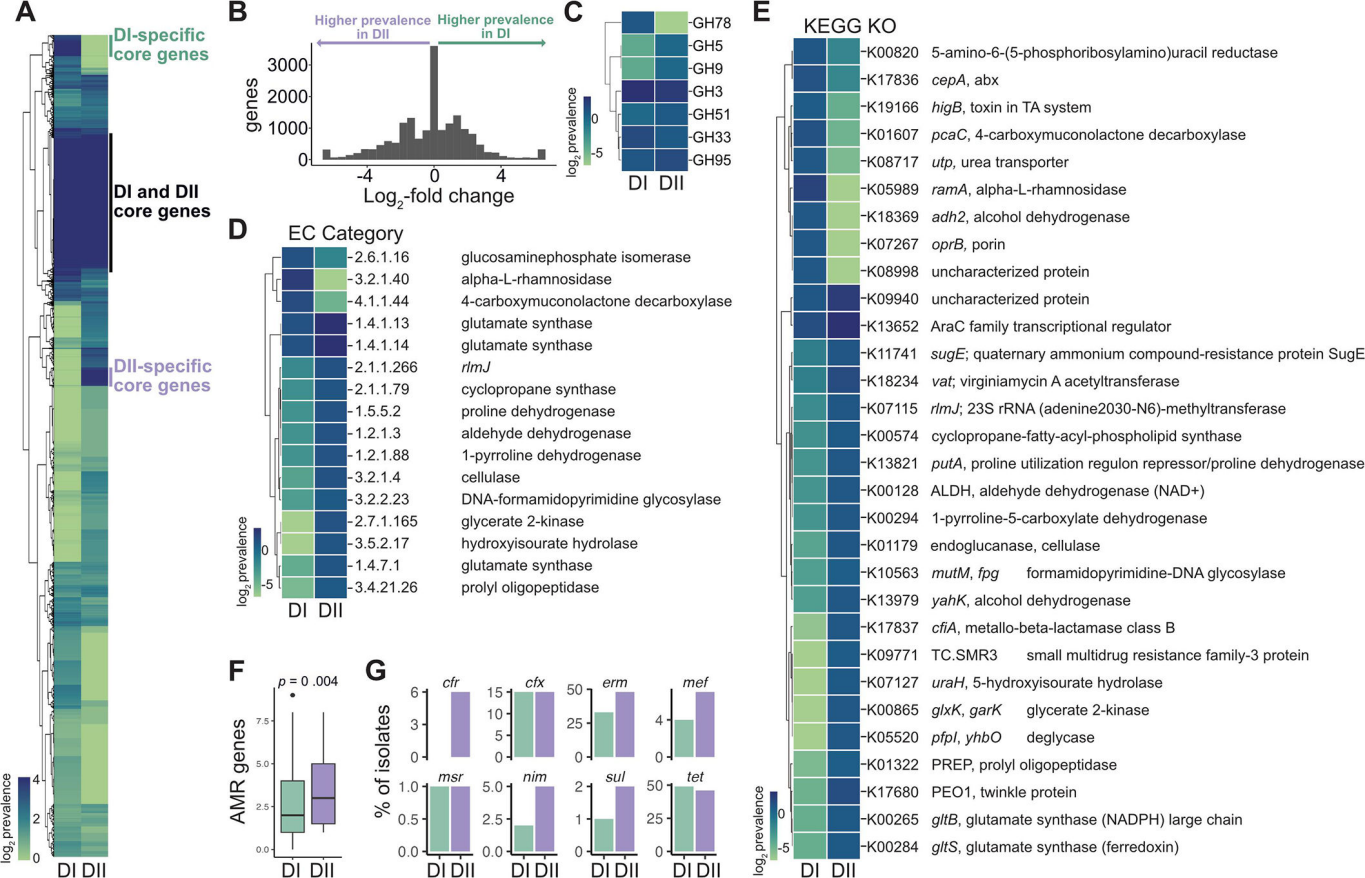
*B. fragilis* divisions I and II segregated by multiple differentially abundant genes and gene categories. (**A**) Relative log_2_ gene abundance heatmap by division, where genes are clustered by R pheatmap complete method, annotated by regions of gene clusters core to both divisions, core only to division I, or core only to division II. (**B**) Histogram of log_2_-fold change of prevalence between all genes in division I versus II. (**C-E**) Log_2_ average number of genes per isolate in categories, (**C**) carbohydrate-active enzymes (CAZy) (log_2_-fold change ≥0.5), (**D**) EC category (log_2_-fold change ≥1), and (E) KEGG KO (log_2_-fold change ≥0.5) between divisions I and II, displaying categories significant by Kruskal–Wallis test (corrected *P* ≤ 0.01). Legend is log_2_ average number of genes per isolate in each category. (**F**) Total number of antimicrobial resistance (AMR) genes per isolate for each division; *P* = 0.004, Welch’s *t*-test. (**G**) The percentage of isolates per division with each antimicrobial resistance gene. *cfr,* chloramphenicol–florfenicol resistance gene*; cfx,* cefuroxime resistance gene*, erm,* erythromycin resistance gene; *mef,* macrolide efflux gene; *msr,* macrolide efflux gene*, nim,* nitroimidazole resistance gene; *sul,* sulfonamide resistance gene*; tet,* tetracycline resistance gene.

Using Panpiper (19), we compared the pangenomes of *B. fragilis* division I and II, and identified 794 genes with differential prevalence, including the exclusive presence of *cfiA* in division II and *cepA* in division I (Fig. 2A, B and E; Table S3) (2, 4). We next assessed the differential abundance of carbohydrate-active enzymes, along with reference metabolic (EC) and reference KEGG orthology pathways (KEGG KO) (Fig. 2C through E). Our analysis reveals division-specific metabolic capabilities and potential ecological niches. Division II strains have genes favoring the degradation of plant cell walls, including glycosyl hydrolases (GH5, GH9, GH51, and GH95) (Fig. 2C), suggesting adaptation to dietary variations. Specifically, BFAG_03498 (ko:K01179, GH9) is predicted to mediate the breakdown of cellulose, BFAG_02344 (GH51) is involved in the breakdown of arabinose-containing polysaccharides, and BFAG_0465 (GH95), an alpha-L-fucosidase, is involved in the cleavage of internal beta-1,4-glycosidic bonds present in plant cell walls (20) (Table S3). One possible explanation for the higher prevalence of plant cell wall degradation genes in division II strains may be dietary differences among hosts of divisions I and II, potentially linked to their distinct geographical distributions (Fig. 1D and E) (21). Division I strains harbor genes indicative of complex carbohydrate degradation, a hallmark feature of gut-resident commensal *Bacteroides* (1, 22). This includes two predicted alpha-L-rhamnosidases (BF9343_0522, BF9343_0310; GH78), which are core genes exclusive to division I (Fig. 2C; Table S3). Division I strains also exhibit an enrichment of GH33 sialidases (Fig. 2C), which catalyze the cleavage of terminal sialic acid residue. Although sialidases have been linked to virulence (23), the *B. fragilis* GH33 sialidase mediates intestinal colonization and persistence during early life (24). Because sialic acid is identified in capsular polysaccharides and lipooligosaccharides (25), its presence may influence colonization and interactions within the host. Division I strains are also enriched in the type VI secretion system GA3, with 84.4% of division I strains having all T6SSiii GA3 structural genes (BF9343_1919–1925, 1931, 1940–1943) (26) compared with 48.1% in division II. This system, exclusive to *B. fragilis*, is recognized for mediating intra-strain competition and colonization dynamics (27–29). The differential abundance of glycosyl hydrolases and T6SSiii GA3 suggests distinct colonization strategies between division I and II strains within the gut.

Division I and II strains may occupy distinct ecological niches, distinguished by genes associated with metabolism and pathogenicity. Division II strains exhibit an increased abundance in genes related to proline degradation and glutamate synthesis pathways (EC 3.4.21.26, BFAG_03703; EC 1.5.5.2, BFAG_03859) (Fig. 2D; Table S3). Additionally, these strains have an increased abundance of the gene encoding DNA-formamidopyrimidine glycosylase (EC 3.2.2.23, BFAG_03121), crucial for DNA repair mechanisms against mutagenesis and cell death induced by alkylating agents (Fig. 2D; Table S3). We also observed differential prevalence in genes and pathways related to multidrug resistance. Division I strains have an increased prevalence of gamma-carboxymuconolactone decarboxylase (EC 4.1.1.44) (Fig. 2D) associated with the breakdown of aromatic compounds and antimicrobial resistance (AMR) (30). We indentify a putative erythromycin esterase that detoxifies macrolides also more abundant in division I (31). Conversely, division II strains have a higher abundance of efflux proteins (K09771, K11741) (Fig. 2E; Table S3) and virginiamycin A acetyltransferase (*vat,* K18234), providing resistance to streptogramins (Fig. 2E; Table S3). Indeed, division II strains harbor a higher number of known AMR genes per isolate compared with division I (*P* = 0.004) (Fig. 2F and G), indicating a potential for increased virulence. Further experimental studies are necessary to determine the functional impact of division-specific genes to understand their roles and interactions within the intestinal ecosystem and host. Collectively, our comparative genomics study unveils distinct geographical distribution and genetic signatures within *B. fragilis* divisions, offering insights into their intricate interactions with the host and respective ecological niches.

## SUPPLEMENTAL MATERIAL

Supplemental methods msystems.00516-24-s0001.docx Additional experimental details and methods.

Table S1 msystems.00516-24-s0002.docx Isolation data for newly isolated and/or sequenced strains.

Table S2 msystems.00516-24-s0003.docx Accession numbers and source for *B. fragilis* strains from public repositories.

Table S3 msystems.00516-24-s0004.docx Genes differentially prevalent between division I and II strains.

ASM does not own the copyrights to Supplemental Material that may be linked to, or accessed through, an article. The authors have granted ASM a non-exclusive, world-wide license to publish the Supplemental Material files. Please contact the corresponding author directly for reuse.
