# Supplementary material for: Pangenome comparison of Bacteroides fragilis genomospecies unveils genetic diversity and ecological insights
Source: mSystems. 2024 Jun 27;9(7):e00516-24. doi: 10.1128/msystems.00516-24 (PMC11265264; doi:10.1128/msystems.00516-24)
Supplement: Supplemental methods — Additional experimental details and methods. [file msystems.00516-24-s0001.docx]

**SUPPLEMENTAL METHODS**

**Isolation source and metadata on newly deposited strains**

*Bacteroides fragilis* strain NCTC9343 was obtained from the American Type Culture Collection (ATCC). We obtained a cohort of historical strains from Eric Martens (University of Michigan) (Pudlo et al., 2022), originally from Abigail Salyers (University of Illinois at Urbana-Champaign) (Shoemaker et al., 2001). All strains sequenced for the first time in this study are listed in Supplementary Table 1.

Samples from healthy donors and patients were collected with the approval of the University of California San Diego Institutional Research Board, with written informed consent signed by subjects prior to sample collection. Healthy and patient stool samples were collected under IRB #141853, #150675, and #190012, and extra-intestinal *B. fragilis* strains were collected from the UC San Diego Clinical Microbiology Facility under IRB# 160524. *Bacteroides fragilis* strains were isolated from approximately 0.5 g fecal materials, homogenized in sterile 30% glycerol, diluted 1:10, and plated in BHI-S with gentamicin (100 µg/mL). Colonies were picked from BHI-S plates and identities were determined using *Bacteroides* species-specific primers by qPCR and verified by Sanger sequencing using primers for 16S rRNA (27F/1492R). *B. fragilis* strains were banked in glycerol and stored in -80 ºC for downstream whole genome sequencing. More detailed metadata information for each newly sequenced strain is available in Supplementary Table 1. Public strains were obtained from the NCBI repository for *Bacteroides fragilis* samples.

**Metagenomic Library Preparation and Sequencing**

All strains were grown in brain heart infusion (BHI) broth (BD Biosciences) supplemented with 5 μg/ml hemin (Sigma) and 0.5 μg/ml Vitamin K (Sigma) in anaerobic conditions (10% H_2_, 10% CO_2_, 80% N2; Coy Lab Products) at 37 °C in.  DNA was extracted from an overnight culture with the MagMAX CORE Nucleic Acid Purification Kit (ThermoFisher), as described previously (Shaffer et al., 2021).

Shotgun sequencing libraries were prepared following using the KAPA HyperPlus (Roche) library preparation protocol (Sanders et al., 2019). Briefly, during the KAPA HyperPlus protocol, with a Mosquito HV micropipetting robot (SPT Labtech), reagents for enzymatic fragmentation, end-repair and A-tailing, and adaptor ligation were added at a 1:10 ratio of the recommended volumes. An equal volume of each sample was pooled with an Echo 550 acoustic liquid handler (Labcyte) after quantification using the PicoGreen™ dsDNA Assay Kit (Thermo Fisher Scientific). The QIAquick PCR Purification Kit (QIAGEN) was used for PCR clean up. Size selection was done with a Pippin Prep electrophoresis instrument (Sage Sciences) to obtain fragments of 300-700 bp. High Sensitivity D1000 Tapestation (Agilent, 5067-5584 & 5067-5585) was used to determine quality. The pool concentration was sequenced on a low-ouput iSeq v2 (300 cycle) (Illumina). From the iSeq run, raw reads were demultiplexed and normalized pooling values were calculated based on read counts to pool the original libraries, repeating PCR cleanup and size-selection to 300-700 bp (Brennan et al., 2023). This pool was sequenced with an Illumina NovaSeq 6000 with 2 x 150 bp chemistry at the Institute for Genomic Medicine (IGM) at UC San Diego.

After raw sequence reads were generated from the NovaSeq, BCL files were demultiplexed, quality filtered, and human sequence depleted (Chen et al., 2018; Li, 2018). Resulting FASTQ were uploaded into Qiita (Gonzalez et al., 2018) study ID #14360 (<https://qiita.ucsd.edu/public/?study_id=14360>).

**Pangenomic analysis with Panpiper**

We analyzed 694 whole genomes sequences isolated either for the first time (Table 1) or publicly available isolates (Table 2). Isolate assembly, quality control, and annotation were conducted with Panpiper (Oles, 2023). In brief, assembly was conducted by Shovill (Seemann, 2022). Quality control was conducted with CheckM2 (Chklovski et al., 2023) with filters of >95% completeness and <5% contamination. FastANI (Jain et al., 2018) was used as an additional quality control for sequence similarity with a ≥ 95% similarity threshold for all strains to a reference (NCTC 9343 for division I and 3_1_12 for division II). Bakta was used for annotation (Schwengers et al., 2021), and Panaroo was used to create a division I pangenome, division II pangenome, and a combined division I, division II pangenome (Tonkin-Hill et al., 2020). For each pangenome, we created a phylogenetic tree of the core genome alignment by maximum likelihood, midpoint rooted. For the division I versus II comparisons, we used MASH (Abram et al., 2021) to create a similarity measurement between each pair of samples. We converted this into a distance matrix and conducted a metric multidimensional scaling (mMDS). We assessed significance between the divisions through PERMANOVA.

**Genome size and GC content analyses**

We measured GC content and genome size differences between division I and II through Welch’s t-test with unequal variance using the data from the results of the CheckM2 analysis for each assembly. We also measured the GC content of all genes that are just in the core genome by selecting each core gene and extracting the sequence for each isolate we analyzed. We also conducted the same analysis for subsets of core genes, i.e. those present in both divisions and those present just in a single division. We assessed significance between these groups through Welch’s t-test. We compared differences in metadata categories between the divisions with Fisher’s Exact Test. We found the percentage of division I and II isolates that were isolated from each continent. We also found the percentage of division I and II isolates that were isolated from the blood, fecal, abscesses, or other. We utilized the Pasolli, 2019 SGB dataset to analyze metadata associated with bins classified as *B. fragilis* (Pasolli et al., 2019)*.* We downloaded all 502 bins labeled as *B. fragilis*, identifying them as division I or II through their percent identity to a reference (NCTC 9343 for division I and 3_1_12 for division II). We then used the Pasolli metadata to determine which bins came from which continents.

**Gene differential abundance**

We analyzed genomic differences between division I and II testing for differential prevalence using the gene presence/absence matrix of the full division I and II pangenome (adjusted Kruskal–Wallis p-value ≤ 0.05 and log-fold change ≥ 2). We then assessed the differential abundance of carbohydrate-active enzymes, along with reference metabolic (EC) and reference KEGG orthology pathways (KEGG KO) with the gene matrices of the pangenomes of division I and II, determining significant differential abundance by a Kruskal-Wallis test (corrected p ≤ 0.01). We identified specific genes of interest through the Bakta annotation, referencing both Refseq, Locus_tag, and Uniref. We identified genes homologous to the Type VI secretion system (T6SS) GA3 in NCTC9343, the type strain of *B. fragilis,* using the locus tags BF9343_1919-1925, 1931, 1940-1943 with blastn homology of greater than 90% over greater than 50% of the length of the gene (Robitaille et al., 2023). We acknowledge that the T6SS GA3 system is highly heterogenous, and some strains may have evolved a version of this operon that may not be detected as homologous to the NCTC 9343 system; therefore, for the purpose of this paper, we just define presence of T6SS GA 3 in terms of the system present in NCTC 9343. We identified antimicrobial resistance genes through AMRFinder (Feldgarden et al., 2021), comparing the average number of antimicrobial resistance genes in the two divisions through Welch’s t-test.

**REFERENCES (for supplemental methods)**

Abram, K., Udaondo, Z., Bleker, C., Wanchai, V., Wassenaar, T. M., Robeson, M. S., & Ussery, D. W. (2021). Mash-based analyses of Escherichia coli genomes reveal 14 distinct phylogroups. *Communications Biology*, *4*(1), Article 1. https://doi.org/10.1038/s42003-020-01626-5

Brennan, C., Salido, R. A., Belda-Ferre, P., Bryant, M., Cowart, C., Tiu, M. D., González, A., McDonald, D., Tribelhorn, C., Zarrinpar, A., & Knight, R. (2023). *Maximizing the potential of high-throughput next-generation sequencing through precise normalization based on read count distribution*. *8*(4).

Chen, S., Zhou, Y., Chen, Y., & Gu, J. (2018). fastp: An ultra-fast all-in-one FASTQ preprocessor. *Bioinformatics*, *34*(17), i884–i890. https://doi.org/10.1093/bioinformatics/bty560

Chklovski, A., Parks, D. H., Woodcroft, B. J., & Tyson, G. W. (2023). CheckM2: A rapid, scalable and accurate tool for assessing microbial genome quality using machine learning. *Nature Methods*, *20*(8), Article 8. https://doi.org/10.1038/s41592-023-01940-w

Feldgarden, M., Brover, V., Gonzalez-Escalona, N., Frye, J. G., Haendiges, J., Haft, D. H., Hoffmann, M., Pettengill, J. B., Prasad, A. B., Tillman, G. E., Tyson, G. H., & Klimke, W. (2021). AMRFinderPlus and the Reference Gene Catalog facilitate examination of the genomic links among antimicrobial resistance, stress response, and virulence. *Scientific Reports*, *11*(1), 12728. https://doi.org/10.1038/s41598-021-91456-0

Gonzalez, A., Navas-Molina, J. A., Kosciolek, T., McDonald, D., Vázquez-Baeza, Y., Ackermann, G., DeReus, J., Janssen, S., Swafford, A. D., Orchanian, S. B., Sanders, J. G., Shorenstein, J., Holste, H., Petrus, S., Robbins-Pianka, A., Brislawn, C. J., Wang, M., Rideout, J. R., Bolyen, E., … Knight, R. (2018). Qiita: Rapid, web-enabled microbiome meta-analysis. *Nature Methods*, *15*(10), 796–798. https://doi.org/10.1038/s41592-018-0141-9

Jain, C., Rodriguez-R, L. M., Phillippy, A. M., Konstantinidis, K. T., & Aluru, S. (2018). High throughput ANI analysis of 90K prokaryotic genomes reveals clear species boundaries. *Nature Communications*, *9*(1), Article 1. https://doi.org/10.1038/s41467-018-07641-9

Li, H. (2018). Minimap2: Pairwise alignment for nucleotide sequences. *Bioinformatics*, *34*(18), 3094–3100. https://doi.org/10.1093/bioinformatics/bty191

Oles R. 2023. rolesucsd/Panpiper. GitHub repository. DOI:10.5281/zenodo.11186447

Pasolli, E., Asnicar, F., Manara, S., Zolfo, M., Karcher, N., Armanini, F., Beghini, F., Manghi, P., Tett, A., Ghensi, P., Collado, M. C., Rice, B. L., DuLong, C., Morgan, X. C., Golden, C. D., Quince, C., Huttenhower, C., & Segata, N. (2019). Extensive Unexplored Human Microbiome Diversity Revealed by Over 150,000 Genomes from Metagenomes Spanning Age, Geography, and Lifestyle. *Cell*, *176*(3), 649-662.e20. https://doi.org/10.1016/j.cell.2019.01.001

Pudlo, N. A., Urs, K., Crawford, R., Pirani, A., Atherly, T., Jimenez, R., Terrapon, N., Henrissat, B., Peterson, D., Ziemer, C., Snitkin, E., & Martens, E. C. (2022). Phenotypic and Genomic Diversification in Complex Carbohydrate-Degrading Human Gut Bacteria. *mSystems*, *7*(1), e0094721. https://doi.org/10.1128/msystems.00947-21

Robitaille, Sophie, Emilia L. Simmons, Adrian J. Verster, Emily Ann McClure, Darlene B. Royce, Evan Trus, Kerry Swartz, et al. 2023. “Community Composition and the Environment Modulate the Population Dynamics of Type VI Secretion in Human Gut Bacteria.” Nature ecology & evolution 7(12): 2092–2107. doi:[10.1038/s41559-023-02230-6](https://doi.org/10.1038/s41559-023-02230-6).

Sanders, J. G., Nurk, S., Salido, R. A., Minich, J., Xu, Z. Z., Zhu, Q., Martino, C., Fedarko, M., Arthur, T. D., Chen, F., Boland, B. S., Humphrey, G. C., Brennan, C., Sanders, K., Gaffney, J., Jepsen, K., Khosroheidari, M., Green, C., Liyanage, M., … Knight, R. (2019). Optimizing sequencing protocols for leaderboard metagenomics by combining long and short reads. *Genome Biology*, *20*(1), 226. https://doi.org/10.1186/s13059-019-1834-9

Schwengers, O., Jelonek, L., Dieckmann, M. A., Beyvers, S., Blom, J., & Goesmann, A. (2021). Bakta: Rapid and standardized annotation of bacterial genomes via alignment-free sequence identification. *Microbial Genomics*, *7*(11), 000685. https://doi.org/10.1099/mgen.0.000685

Seemann, T. (2022). *Shovill* [Perl]. https://github.com/tseemann/shovill (Original work published 2016)

Shaffer, J. P., Marotz, C., Belda-Ferre, P., Martino, C., Wandro, S., Estaki, M., Salido, R. A., Carpenter, C. S., Zaramela, L. S., Minich, J. J., Bryant, M., Sanders, K., Fraraccio, S., Ackermann, G., Humphrey, G., Swafford, A. D., Miller-Montgomery, S., & Knight, R. (2021). A comparison of DNA/RNA extraction protocols for high-throughput sequencing of microbial communities. *Biotechniques*, *70*(3), 149–159. https://doi.org/10.2144/btn-2020-0153

Shoemaker, N. B., Vlamakis, H., Hayes, K., & Salyers, A. A. (2001). Evidence for Extensive Resistance Gene Transfer amongBacteroides spp. And among Bacteroides and Other Genera in the Human Colon. *Applied and Environmental Microbiology*, *67*(2), 561–568. https://doi.org/10.1128/AEM.67.2.561-568.2001

Tonkin-Hill, G., MacAlasdair, N., Ruis, C., Weimann, A., Horesh, G., Lees, J. A., Gladstone, R. A., Lo, S., Beaudoin, C., Floto, R. A., Frost, S. D. W., Corander, J., Bentley, S. D., & Parkhill, J. (2020). Producing polished prokaryotic pangenomes with the Panaroo pipeline. *Genome Biology*, *21*(1), 180. https://doi.org/10.1186/s13059-020-02090-4
