# Supplementary material for: Pangenome comparison of Bacteroides fragilis genomospecies unveils genetic diversity and ecological insights
Source: mSystems. 2024 Jun 27;9(7):e00516-24. doi: 10.1128/msystems.00516-24 (PMC11265264; doi:10.1128/msystems.00516-24)
Supplement: Table S1 — Isolation data for newly isolated and/or sequenced strains. [file msystems.00516-24-s0002.docx]

Sample Chu Sample_Name Division Group Site City State Country Continent Year.Isolated Host GC Genome.size scaffolds

363192522_S5_L001 HCBf049 FIT025E3CAL P2-G8 1 IBD Fecal San_Diego Califonia United States of America North America 2020 Human 0.436167927 5394365 148

363192527_S10_L001 HCBf014 112-3 1 Healthy Fecal San_Diego California United States of America North America 2020 Human 0.434902254 5685650 284

363192529_S12_L001 HCBf038 FIT073E3CAL B11 1 IBD Fecal San_Diego Califonia United States of America North America 2020 Human 0.435801279 5373160 108

363192533_S31_L001 HCBf076 RA24-trans B4 1 Healthy Colon San_Diego California United States of America North America 2020 Human 0.433842284 5221787 100

363192541_S20_L001 HCBf039 FIT073E3CAL B10 1 IBD Fecal San_Diego Califonia United States of America North America 2020 Human 0.434686437 6105470 282

363192548_S39_L001 HCBf077 RA28-trans GS4 1 Healthy Colon San_Diego California United States of America North America 2020 Human 0.435539167 5276297 63

363192555_S46_L001 HCBf066 FIT037SC1.C3 A3 1 IBD Fecal San_Diego Califonia United States of America North America 2020 Human 0.427964312 5913902 294

363192556_S47_L001 HCBf079 RA30-trans G8 1 Healthy Colon San_Diego California United States of America North America 2020 Human 0.434519744 5097193 38

363192557_S48_L001 HCBf090 12-PL 1 Infection Tissue San_Diego Califonia United States of America North America 2020 Human 0.437349244 5748111 56

363192558_S49_L001 HCBf007 110-29 1 Healthy Fecal San_Diego California United States of America North America 2020 Human 0.429790445 5347050 65

363192563_S54_L001 HCBf067 FIT037SC1.C3 C5 1 IBD Fecal San_Diego Califonia United States of America North America 2020 Human 0.431725755 4999799 84

363192564_S55_L001 HCBf078 RA30-trans G6 1 Healthy Colon San_Diego California United States of America North America 2020 Human 0.434534717 5098122 42

363192566_S57_L001 HCBf008 110-31 1 Healthy Fecal San_Diego California United States of America North America 2020 Human 0.42982641 5351244 68

363192571_S62_L001 HCBf069 FIT122SC1.E3 E1 1 IBD Fecal San_Diego Califonia United States of America North America 2020 Human 0.432807904 5101039 24

363192573_S64_L001 HCBf092 BAC06 1 Infection Bone San_Diego Califonia United States of America North America 2020 Human 0.430989429 5347630 28

363192577_S68_L001 HCBf045 FIT268E3CAL C1 1 IBD Fecal San_Diego Califonia United States of America North America 2020 Human 0.433865036 5332119 35

363192578_S69_L001 HCBf057 FIT134E3CAL P1-G2 1 IBD Fecal San_Diego Califonia United States of America North America 2020 Human 0.430974419 5241564 34

363192579_S70_L001 HCBf068 FIT122SC1.E3 D1 1 IBD Fecal San_Diego Califonia United States of America North America 2020 Human 0.432822559 5100425 29

363192581_S72_L001 HCBf093 BAC07 1 Infection Swab San_Diego Califonia United States of America North America 2020 Human 0.432146454 5233050 48

363192585_S76_L001 HCBf046 FIT268E3CAL C2 1 IBD Fecal San_Diego Califonia United States of America North America 2020 Human 0.433469104 5488435 48

363192587_S78_L001 HCBf070 FIT104SC1.E3 B10 1 IBD Fecal San_Diego Califonia United States of America North America 2020 Human 0.432496617 5291757 45

363192589_S80_L001 HCBf094 BAC09 1 Infection Drainage San_Diego Califonia United States of America North America 2020 Human 0.432604006 5266040 26

363192592_S83_L001 HCBf035 FIT073E3CAL B7 1 IBD Fecal San_Diego Califonia United States of America North America 2020 Human 0.435715161 5339906 60

363192594_S85_L001 HCBf060 Non-IBD Sample C2 C5 1 Healthy Fecal San_Diego Califonia United States of America North America 2020 Human 0.434028378 5217410 57

363192595_S86_L001 HCBf071 FIT104SC1.E3 C2 1 IBD Fecal San_Diego Califonia United States of America North America 2020 Human 0.432343791 5281836 47

363192597_S88_L001 HCBf095 S01-0001-1 1 Healthy Fecal San_Diego Califonia United States of America North America 2020 Human 0.432820276 5246263 70

363192598_S89_L001 HCBf012 111-20 1 Healthy Fecal San_Diego California United States of America North America 2020 Human 0.432800498 5058222 45

363192600_S91_L001 HCBf036 FIT073E3CAL B8 1 IBD Fecal San_Diego Califonia United States of America North America 2020 Human 0.435669449 5339819 68

363192601_S92_L001 HCBf048 FIT236E3CAL 1 IBD Fecal San_Diego Califonia United States of America North America 2020 Human 0.434894555 5292929 64

363192602_S93_L001 HCBf059 Non-IBD Sample C2 B6 1 Healthy Fecal San_Diego Califonia United States of America North America 2020 Human 0.434016842 5225341 54

363192603_S94_L001 HCBf072 FIT291SC1.E3 A3 1 IBD Fecal San_Diego Califonia United States of America North America 2020 Human 0.432778245 4837965 37

363192604_S95_L001 HCBf084 1-PL 1 Infection Drainage San_Diego Califonia United States of America North America 2020 Human 0.434849016 5161173 53

363192605_S96_L001 HCBf096 BAC14 1 Infection Swab San_Diego Califonia United States of America North America 2020 Human 0.433709946 5394663 27

363192793_S98_L001 HCBf109 BAC52 1 Infection Swab San_Diego Califonia United States of America North America 2020 Human 0.432727547 5511565 205

363192794_S99_L001 HCBf121 BAC89 1 Infection Aspirate San_Diego Califonia United States of America North America 2020 Human 0.434926395 5290778 69

363192998_S106_L001 HCBf110 BAC72 1 Infection Lesion San_Diego Califonia United States of America North America 2020 Human 0.435706994 5620137 162

363192999_S107_L001 HCBf122 BAC92 1 Infection Drainage San_Diego Califonia United States of America North America 2020 Human 0.433136523 5351696 204

363193002_S105_L001 HCBf098 BAC18 1 Infection Lesion San_Diego Califonia United States of America North America NA Human 0.430584706 5318194 123

363193004_S114_L001 HCBf111 BAC73 1 Infection Drainage San_Diego Califonia United States of America North America 2020 Human 0.430888477 6210918 706

363193027_S137_L001 HCBf102 BAC36 1 Infection NA San_Diego Califonia United States of America North America 2020 Human 0.435267647 5322663 36

363193028_S138_L001 HCBf114 BAC77 1 Infection Swab San_Diego Califonia United States of America North America 2020 Human 0.435407579 5409853 53

363193035_S145_L001 HCBf103 BAC37 1 Infection Swab San_Diego Califonia United States of America North America 2020 Human 0.434304869 5258822 40

363193036_S146_L001 HCBf115 BAC79 1 Infection Tissue San_Diego Califonia United States of America North America 2020 Human 0.43114502 5136680 30

363193043_S153_L001 HCBf104 BAC41 1 Infection Swab San_Diego Califonia United States of America North America 2020 Human 0.434768044 5310802 104

363193044_S154_L001 HCBf116 BAC80 1 Infection Swab San_Diego Califonia United States of America North America 2020 Human 0.432374097 5289519 42

363193053_S162_L001 HCBf117 BAC81 1 Infection Aspirate San_Diego Califonia United States of America North America 2020 Human 0.434927052 5292276 62

363193059_S169_L001 HCBf106 BAC44 1 Infection Tissue San_Diego Califonia United States of America North America 2020 Human 0.434427469 5301778 55

363193060_S170_L001 HCBf118 BAC83 1 Infection Bed Sore San_Diego Califonia United States of America North America 2020 Human 0.431808092 5182939 40

363193068_S178_L001 HCBf119 BAC86 1 Infection Aspirate San_Diego Califonia United States of America North America 2020 Human 0.435055339 5395355 66

363193076_S185_L001 HCBf108 BAC60 1 Infection Tissue San_Diego Califonia United States of America North America 2020 Human 0.438323971 5284468 64

363193756_S6_L001 HCBf061 Non-IBD Sample C6 D7 1 Healthy Fecal San_Diego Califonia United States of America North America 2020 Human 0.435818255 5365521 112

363193757_S8_L001 HCBf085 2-PL 1 Infection Foot San_Diego Califonia United States of America North America 2020 Human 0.432126286 5307828 74

363193763_S14_L001 HCBf062 Non-IBD Sample C6 E10 1 Healthy Fecal San_Diego Califonia United States of America North America 2020 Human 0.436317788 5891708 320

363197065_S315_L001 HCBf189 112-16 1 Healthy Fecal San_Diego California United States of America North America 2020 Human 0.436180631 5477522 447

363197827_S378_L001 HCBf194 111-9 1 Healthy Fecal San_Diego California United States of America North America 2020 Human 0.432817324 5056110 44

MCT420_10_S18_L002 HCBf213 J665_Rhesus 1 Animal Fecal San Diego California United States of America North America 2022 Rhesus 0.434345169 5129356 42

MCT420_18_S26_L002 HCBf159 YCH46 1 Infection Blood San Diego California United States of America North America 1960 Human 0.431517144 5261492 41

MCT420_2_S10_L002 HCBf209 infant_twin 1 1 Healthy Fecal San Diego California United States of America North America 2022 Human 0.431447295 5228138 183

MCT420_28_S36_L002 HCBf251 US398 1 Infection Blood San Diego California United States of America North America NA Human 0.433780644 5012326 90

MCT420_29_S37_L002 HCBf016 112-15 1 Healthy Fecal San Diego California United States of America North America 2020 Human 0.435876793 5220606 92

MCT420_32_S40_L002 HCBf013 111-28 1 Healthy Fecal San Diego California United States of America North America 2020 Human 0.432801971 5055617 53

MCT420_35_S43_L002 HCBf112 BAC74 1 Infection Abscess San Diego California United States of America North America 2020 Human 0.432676043 5238425 70

MCT420_36_S44_L002 HCBf074 RA44-A5 1 IBD Colon San Diego California United States of America North America 2020 Human 0.431970913 5359090 44

MCT420_41_S49_L002 HCBf087 6-PL 1 Infection Fluid San Diego California United States of America North America 2020 Human 0.430088484 5186363 63

MCT420_47_S55_L002 HCBf037 FIT073E3CAL B9 1 IBD Fecal San Diego California United States of America North America 2020 Human 0.436014688 5355315 104

MCT420_49_S57_L002 HCBf107 BAC45 1 Infection NA San Diego California United States of America North America 2020 Human 0.433603335 5151697 65

MCT420_50_S58_L002 HCBf132 VPI-BF8371 1 Unknown NA San Diego California United States of America North America 1960 Human 0.435636945 5252897 38

MCT420_52_S60_L002 HCBf216 RA 49 1 Healthy Colon San Diego California United States of America North America 2022 Human 0.434665587 5384008 107

MCT420_54_S62_L002 HCBf218 RA 87 1 IBD Colon San Diego California United States of America North America 2022 Human 0.433365899 5214072 37

MCT420_55_S63_L002 HCBf219 RA 53 1 IBD Colon San Diego California United States of America North America 2022 Human 0.433232555 5107594 31

MCT420_56_S64_L002 HCBf220 RA 56 1 Healthy Colon San Diego California United States of America North America 2022 Human 0.433405184 5329574 52

MCT420_58_S66_L002 HCBf222 RA 80 1 Healthy Colon San Diego California United States of America North America 2022 Human 0.433689137 5233034 328

MCT420_59_S67_L002 HCBf223 RA 81 1 Healthy Colon San Diego California United States of America North America 2022 Human 0.431718992 5276145 36

MCT420_6_S14_L002 HCBf252 B35 1 Infection Fecal San Diego California United States of America North America NA Human 0.431552188 5161867 36

MCT420_60_S68_L002 HCBf224 RA 52 1 Healthy Colon San Diego California United States of America North America 2022 Human 0.432416477 5301810 43

MCT420_63_S71_L002 HCBf227 RA56 1 Healthy Colon San Diego California United States of America North America NA Human 0.433451914 5335593 44

MCT420_7_S15_L002 HCBf253 12905-23V 1 Infection Abscess San Diego California United States of America North America NA Human 0.432083406 5405542 106

MCT420_75_S83_L002 HCBf239 PA5 1 Infection Fecal San Diego California United States of America North America NA Human 0.43371433 5042568 40

MCT420_76_S84_L002 HCBf240 CL10T01C02 1 Healthy Fecal San Diego California United States of America North America NA Human 0.434774916 5250894 57

MCT420_77_S85_L002 HCBf241 CM12 1 Infection Abscess San Diego California United States of America North America NA Human 0.431298338 5205580 133

MCT420_79_S87_L002 HCBf167 CL07T12C05 1 Healthy Fecal San Diego California United States of America North America NA Human 0.436048888 5431008 262

MCT420_8_S16_L002 HCBf 9343 1 Infection Abscess San Diego California United States of America North America NA Human 0.430839408 5221702 105

MCT420_80_S88_L002 HCBf244 CL13T01C12 1 Healthy Fecal San Diego California United States of America North America NA Human 0.432059052 5371525 235

MCT420_81_S89_L002 HCBf245 US390 1 Infection Abscess San Diego California United States of America North America NA Human 0.432739298 5248793 51

MCT420_86_S94_L002 HCBf250 CMR2896 1 Unknown NA San Diego California United States of America North America NA Human 0.431845916 5478153 279

363192520_S3_L001 HCBf025 S01-0228-1 (Alm) 1 Healthy Fecal Boston Massachusetts United States of America North America 2010 Human 0.432902757 5224857 41

363192528_S11_L001 HCBf026 S03-0090-12 (Alm) 1 Healthy Fecal Boston Massachusetts United States of America North America 2010 Human 0.431553967 5324516 29

363192539_S19_L001 HCBf027 S05-0002-1 (Alm) 1 Healthy Fecal Boston Massachusetts United States of America North America 2010 Human 0.439624494 6744606 392

363192544_S35_L001 HCBf029 S07-0068-16 (Alm) 1 Healthy Fecal Boston Massachusetts United States of America North America 2010 Human 0.435327988 6313040 503

363192552_S43_L001 HCBf030 S09-0001-16 (Alm) 1 Healthy Fecal Boston Massachusetts United States of America North America 2010 Human 0.435443415 5967478 369

363192559_S50_L001 HCBf019 S04-0107-6 (Alm) 1 Healthy Fecal Boston Massachusetts United States of America North America 2010 Human 0.439033321 5825100 394

363192560_S51_L001 HCBf031 S10-0039-19 (Alm) 1 Healthy Fecal Boston Massachusetts United States of America North America 2010 Human 0.434635426 5753866 246

363192568_S59_L001 HCBf032 S11-0001-18 (Alm) 1 Healthy Fecal Boston Massachusetts United States of America North America 2010 Human 0.431498806 5255215 62

363192583_S74_L001 HCBf022 S02-0024-14 (Alm) 1 Healthy Fecal Boston Massachusetts United States of America North America 2010 Human 0.432760753 5269073 43

363192591_S82_L001 HCBf023 S04-0006-7 (Alm) 1 Healthy Fecal Boston Massachusetts United States of America North America 2010 Human 0.432991006 5259391 52

363192797_S102_L001 HCBf157 WH718 1 Healthy Fecal Boston Massachusetts United States of America North America 1990 Human 0.433105043 6106417 226

363192805_S110_L001 HCBf158 CL03T00C08 1 Healthy Fecal Boston Massachusetts United States of America North America 2000 Human 0.432588101 6183263 283

363193049_S158_L001 HCBf164 WH605 1 Healthy Fecal Boston Massachusetts United States of America North America 1990 Human 0.434869484 5243410 48

363193055_S165_L001 HCBf153 WH705 1 Healthy Fecal Boston Massachusetts United States of America North America 1990 Human 0.43414955 5133397 45

363193056_S166_L001 HCBf165 CL05T00C42 1 Healthy Fecal Boston Massachusetts United States of America North America 2000 Human 0.434964415 5246151 38

363193071_S180_L001 HCBf143 WH706 1 Healthy Fecal Boston Massachusetts United States of America North America 1990 Human 0.434102557 5134395 42

363193073_S182_L001 HCBf167 CL07T12C05 1 Healthy Fecal Boston Massachusetts United States of America North America 2000 Human 0.436015795 5403818 46

363193078_S188_L001 HCBf144 WH707 1 Healthy Fecal Boston Massachusetts United States of America North America 1990 Human 0.433956261 5145113 48

363193080_S190_L001 HCBf168 CL03T12C07 1 Healthy Fecal Boston Massachusetts United States of America North America 2000 Human 0.434152783 5174243 38

363192795_S100_L001 HCBf133 VPI-3277 1 Infection Blood United States of America North America 1960 Human 0.431741987 5348405 84

363193001_S108_L001 HCBf134 VPI-2553 1 Infection Appendix abscess United States of America North America 1960 Human 0.432690297 5351331 104

363193013_S123_L001 HCBf124 VPI-4517 1 Unknown NA Virginia United States of America North America 1960 Human 0.429311771 5998962 333

363193021_S134_L001 HCBf161 3_2_5 1 Healthy Fecal Boston United States of America North America 1990 Human 0.431783013 5152419 44

363193029_S139_L001 HCBf126 VPI-BF7639 1 Unknown NA Virginia United States of America North America 1960 Human 0.431314564 5084171 79

363193032_S142_L001 HCBf162 VPI-29765 1 Unknown NA Virginia United States of America North America 1960 Human 0.430956948 5111376 58

363193037_S147_L001 HCBf127 58_3_6 1 Unknown NA United States of America North America 1960 Human 0.435262558 5589447 191

363193039_S149_L001 HCBf151 VPI-BF8223 1 Unknown NA Virginia United States of America North America 1960 Human 0.433401273 5385178 82

363193040_S150_L001 HCBf163 WAL8790 1 Infection NA United States of America North America 1960 Human 0.434099483 5329594 66

363193045_S155_L001 HCBf128 VPI-5383 / 23745 1 Infection Peritoneal Fluid United States of America North America 1960 Human 0.433391942 5230741 40

363193046_S156_L001 HCBf140 WAL8762 1 Infection NA United States of America North America 1983 Human 0.433955382 5255432 71

363193047_S157_L001 HCBf152 WAL8774 1 Infection NA United States of America North America 1960 Human 0.430663578 5216782 26

363193048_S163_L001 HCBf129 VPI-BF7397 1 Unknown NA Virginia United States of America North America 1960 Human 0.432403455 5097191 31

363193054_S164_L001 HCBf141 VPI-499 1 Unknown NA Virginia United States of America North America 1960 Human 0.42959517 5084154 32

363193061_S171_L001 HCBf130 VPI-BF119 1 Unknown NA Virginia United States of America North America 1960 Human 0.432877752 5281900 40

363193062_S172_L001 HCBf142 638R 1 Infection Intestine United States of America North America 1990 Human 0.433836739 5328569 46

363193064_S174_L001 HCBf166 VPI-2556I 1 Unknown NA Virginia United States of America North America 1960 Human 0.43365842 5113098 38

363193069_S179_L001 HCBf131 VPI-BF7567 1 Unknown NA Virginia United States of America North America 1960 Human 0.432902042 5069819 32

363193072_S181_L001 HCBf155 VPI-BF-AK87 1 Unknown NA Virginia United States of America North America 1960 Human 0.43102986 5273920 33

363193079_S189_L001 HCBf156 VPI-12256 1 Unknown NA Virginia United States of America North America 1960 Human 0.436036296 5342014 48

363192572_S63_L001 HCBf080 RA38-trans GS7 2 Healthy Colon San_Diego California United States of America North America 2020 Human 0.434155703 5001238 29

363192804_S111_L001 HCBf170 3_1_12 2 Healthy NA North America 2007 Human 0.437290836 5825560 449

363193009_S119_L001 HCBf171 VPI-2627-J2 2 Unknown NA Virginia United States of America North America 1960 Human 0.434315959 5131216 196

363193017_S127_L001 HCBf172 VPI-3392 2 Unknown NA Virginia United States of America North America 1960 Human 0.433131318 5427638 90

363193025_S135_L001 HCBf173 VPI-4076 2 Infection Blood NA North America 1960 Human 0.432039593 5497267 71

363193041_S151_L001 HCBf175 VPI-2393 2 Unknown NA Virginia United States of America North America 1960 Human 0.433322431 5484888 60

363193057_S167_L001 HCBf177 VPI-A11-24B 2 Unknown NA Virginia United States of America North America 1960 Human 0.433111405 5238038 62

363193065_S175_L001 HCBf178 VPI-4117 2 Unknown NA Virginia United States of America North America 1960 Human 0.433797345 5284569 53

MCT420_17_S25_L002 HCBf169 VPI-2552 2 Unknown NA United States of America North America 1980 Human 0.431155149 5348323 179

Table 1: Metadata for genomes which were isolated and sequenced including the sequence accession number, host isolation source, name of strain, metadata on assembly quality, and information on isolation source.
