# Supplementary material for: Pangenome comparison of Bacteroides fragilis genomospecies unveils genetic diversity and ecological insights
Source: mSystems. 2024 Jun 27;9(7):e00516-24. doi: 10.1128/msystems.00516-24 (PMC11265264; doi:10.1128/msystems.00516-24)
Supplement: Table S2 — Accession numbers and source for B. fragilis strains from public repositories. [file msystems.00516-24-s0003.docx]

Sample Source

GCA_000009925.1 NCBI Genome Browser

GCA_000025985.1 NCBI Genome Browser

GCA_000157015.1 NCBI Genome Browser

GCA_000162135.1 NCBI Genome Browser

GCA_000210835.1 NCBI Genome Browser

GCA_000218345.1 NCBI Genome Browser

GCA_000263115.1 NCBI Genome Browser

GCA_000269525.1 NCBI Genome Browser

GCA_000273095.1 NCBI Genome Browser

GCA_000273115.1 NCBI Genome Browser

GCA_000273135.1 NCBI Genome Browser

GCA_000273155.1 NCBI Genome Browser

GCA_000273765.1 NCBI Genome Browser

GCA_000297695.1 NCBI Genome Browser

GCA_000297735.1 NCBI Genome Browser

GCA_000297755.1 NCBI Genome Browser

GCA_000598145.1 NCBI Genome Browser

GCA_000598165.1 NCBI Genome Browser

GCA_000598185.2 NCBI Genome Browser

GCA_000598205.1 NCBI Genome Browser

GCA_000598225.1 NCBI Genome Browser

GCA_000598245.1 NCBI Genome Browser

GCA_000598265.1 NCBI Genome Browser

GCA_000598285.1 NCBI Genome Browser

GCA_000598305.1 NCBI Genome Browser

GCA_000598325.1 NCBI Genome Browser

GCA_000598345.1 NCBI Genome Browser

GCA_000598365.1 NCBI Genome Browser

GCA_000598385.1 NCBI Genome Browser

GCA_000598425.1 NCBI Genome Browser

GCA_000598445.1 NCBI Genome Browser

GCA_000598505.1 NCBI Genome Browser

GCA_000598525.1 NCBI Genome Browser

GCA_000598545.2 NCBI Genome Browser

GCA_000598565.1 NCBI Genome Browser

GCA_000598585.1 NCBI Genome Browser

GCA_000598645.1 NCBI Genome Browser

GCA_000598665.1 NCBI Genome Browser

GCA_000598685.2 NCBI Genome Browser

GCA_000598705.1 NCBI Genome Browser

GCA_000598725.1 NCBI Genome Browser

GCA_000598745.1 NCBI Genome Browser

GCA_000598765.2 NCBI Genome Browser

GCA_000598785.2 NCBI Genome Browser

GCA_000598805.1 NCBI Genome Browser

GCA_000598825.1 NCBI Genome Browser

GCA_000598845.1 NCBI Genome Browser

GCA_000598865.1 NCBI Genome Browser

GCA_000598885.1 NCBI Genome Browser

GCA_000598905.1 NCBI Genome Browser

GCA_000598925.1 NCBI Genome Browser

GCA_000598945.1 NCBI Genome Browser

GCA_000598965.1 NCBI Genome Browser

GCA_000598985.1 NCBI Genome Browser

GCA_000599005.1 NCBI Genome Browser

GCA_000599025.1 NCBI Genome Browser

GCA_000599045.1 NCBI Genome Browser

GCA_000599065.2 NCBI Genome Browser

GCA_000599085.1 NCBI Genome Browser

GCA_000599125.1 NCBI Genome Browser

GCA_000599145.2 NCBI Genome Browser

GCA_000599165.2 NCBI Genome Browser

GCA_000599185.2 NCBI Genome Browser

GCA_000599225.1 NCBI Genome Browser

GCA_000599245.1 NCBI Genome Browser

GCA_000599305.1 NCBI Genome Browser

GCA_000599325.1 NCBI Genome Browser

GCA_000599365.1 NCBI Genome Browser

GCA_000599385.1 NCBI Genome Browser

GCA_000601035.1 NCBI Genome Browser

GCA_000601055.1 NCBI Genome Browser

GCA_000601075.2 NCBI Genome Browser

GCA_000601095.1 NCBI Genome Browser

GCA_000601115.1 NCBI Genome Browser

GCA_000710365.3 NCBI Genome Browser

GCA_000710375.3 NCBI Genome Browser

GCA_000724665.3 NCBI Genome Browser

GCA_000724795.2 NCBI Genome Browser

GCA_000724805.2 NCBI Genome Browser

GCA_000724815.2 NCBI Genome Browser

GCA_000965785.1 NCBI Genome Browser

GCA_001054865.1 NCBI Genome Browser

GCA_001054895.1 NCBI Genome Browser

GCA_001056335.1 NCBI Genome Browser

GCA_001058755.1 NCBI Genome Browser

GCA_001058775.1 NCBI Genome Browser

GCA_001077245.1 NCBI Genome Browser

GCA_001286525.1 NCBI Genome Browser

GCA_001580095.1 NCBI Genome Browser

GCA_001580105.1 NCBI Genome Browser

GCA_001682215.1 NCBI Genome Browser

GCA_001692695.1 NCBI Genome Browser

GCA_001693695.1 NCBI Genome Browser

GCA_001695355.1 NCBI Genome Browser

GCA_001699855.1 NCBI Genome Browser

GCA_001699865.1 NCBI Genome Browser

GCA_001699875.1 NCBI Genome Browser

GCA_001699885.1 NCBI Genome Browser

GCA_001816225.2 NCBI Genome Browser

GCA_001997325.1 NCBI Genome Browser

GCA_002810985.1 NCBI Genome Browser

GCA_002810995.1 NCBI Genome Browser

GCA_002811025.1 NCBI Genome Browser

GCA_002811035.1 NCBI Genome Browser

GCA_002811065.1 NCBI Genome Browser

GCA_002811085.1 NCBI Genome Browser

GCA_002849695.1 NCBI Genome Browser

GCA_003363115.1 NCBI Genome Browser

GCA_003436285.1 NCBI Genome Browser

GCA_003436935.1 NCBI Genome Browser

GCA_003437815.1 NCBI Genome Browser

GCA_003438775.1 NCBI Genome Browser

GCA_003438895.1 NCBI Genome Browser

GCA_003439045.1 NCBI Genome Browser

GCA_003439065.1 NCBI Genome Browser

GCA_003439285.1 NCBI Genome Browser

GCA_003439505.1 NCBI Genome Browser

GCA_003439635.1 NCBI Genome Browser

GCA_003439675.1 NCBI Genome Browser

GCA_003458215.1 NCBI Genome Browser

GCA_003458955.1 NCBI Genome Browser

GCA_003462955.1 NCBI Genome Browser

GCA_003463545.1 NCBI Genome Browser

GCA_003463555.1 NCBI Genome Browser

GCA_003464385.1 NCBI Genome Browser

GCA_003464635.1 NCBI Genome Browser

GCA_003465265.1 NCBI Genome Browser

GCA_003468145.1 NCBI Genome Browser

GCA_003469305.1 NCBI Genome Browser

GCA_003469705.1 NCBI Genome Browser

GCA_003471465.1 NCBI Genome Browser

GCA_003471745.1 NCBI Genome Browser

GCA_003471925.1 NCBI Genome Browser

GCA_003472025.1 NCBI Genome Browser

GCA_003472195.1 NCBI Genome Browser

GCA_003473955.1 NCBI Genome Browser

GCA_003475385.1 NCBI Genome Browser

GCA_003475745.1 NCBI Genome Browser

GCA_004798445.1 NCBI Genome Browser

GCA_004798525.1 NCBI Genome Browser

GCA_005706655.1 NCBI Genome Browser

GCA_005844845.1 NCBI Genome Browser

GCA_007896575.1 NCBI Genome Browser

GCA_007896595.1 NCBI Genome Browser

GCA_007896605.1 NCBI Genome Browser

GCA_007896675.1 NCBI Genome Browser

GCA_007896685.1 NCBI Genome Browser

GCA_007896745.1 NCBI Genome Browser

GCA_007896795.1 NCBI Genome Browser

GCA_008086695.1 NCBI Genome Browser

GCA_008369705.1 NCBI Genome Browser

GCA_008569055.1 NCBI Genome Browser

GCA_008569225.1 NCBI Genome Browser

GCA_008569265.1 NCBI Genome Browser

GCA_008569305.1 NCBI Genome Browser

GCA_008569335.1 NCBI Genome Browser

GCA_008569345.1 NCBI Genome Browser

GCA_008569365.1 NCBI Genome Browser

GCA_008569395.1 NCBI Genome Browser

GCA_008569415.1 NCBI Genome Browser

GCA_008569445.1 NCBI Genome Browser

GCA_008829725.1 NCBI Genome Browser

GCA_009024655.1 NCBI Genome Browser

GCA_009025495.1 NCBI Genome Browser

GCA_009025695.1 NCBI Genome Browser

GCA_009025705.1 NCBI Genome Browser

GCA_012844105.1 NCBI Genome Browser

GCA_013267555.1 NCBI Genome Browser

GCA_013327815.1 NCBI Genome Browser

GCA_013327835.1 NCBI Genome Browser

GCA_013694025.1 NCBI Genome Browser

GCA_013867505.1 NCBI Genome Browser

GCA_014042405.1 NCBI Genome Browser

GCA_014042415.1 NCBI Genome Browser

GCA_014042445.1 NCBI Genome Browser

GCA_014042455.1 NCBI Genome Browser

GCA_014042465.1 NCBI Genome Browser

GCA_014042505.1 NCBI Genome Browser

GCA_014042525.1 NCBI Genome Browser

GCA_014639005.1 NCBI Genome Browser

GCA_014893755.1 NCBI Genome Browser

GCA_015070945.1 NCBI Genome Browser

GCA_015547555.1 NCBI Genome Browser

GCA_015548495.1 NCBI Genome Browser

GCA_015548595.1 NCBI Genome Browser

GCA_015548825.1 NCBI Genome Browser

GCA_015550765.1 NCBI Genome Browser

GCA_015551365.1 NCBI Genome Browser

GCA_015553295.1 NCBI Genome Browser

GCA_015553495.1 NCBI Genome Browser

GCA_015553755.1 NCBI Genome Browser

GCA_015556305.1 NCBI Genome Browser

GCA_015558985.1 NCBI Genome Browser

GCA_015560515.1 NCBI Genome Browser

GCA_015669295.1 NCBI Genome Browser

GCA_015669435.1 NCBI Genome Browser

GCA_015831745.1 NCBI Genome Browser

GCA_015831765.1 NCBI Genome Browser

GCA_016623685.1 NCBI Genome Browser

GCA_016864615.1 NCBI Genome Browser

GCA_016888645.1 NCBI Genome Browser

GCA_016889925.1 NCBI Genome Browser

GCA_018292185.1 NCBI Genome Browser

GCA_018369215.1 NCBI Genome Browser

GCA_018786025.1 NCBI Genome Browser

GCA_019013355.1 NCBI Genome Browser

GCA_019041735.1 NCBI Genome Browser

GCA_019041775.1 NCBI Genome Browser

GCA_019041875.1 NCBI Genome Browser

GCA_019130335.1 NCBI Genome Browser

GCA_019130425.1 NCBI Genome Browser

GCA_019131615.1 NCBI Genome Browser

GCA_019131915.1 NCBI Genome Browser

GCA_019633805.1 NCBI Genome Browser

GCA_019633825.1 NCBI Genome Browser

GCA_019633845.1 NCBI Genome Browser

GCA_019633885.1 NCBI Genome Browser

GCA_019633905.1 NCBI Genome Browser

GCA_019633925.1 NCBI Genome Browser

GCA_019633945.1 NCBI Genome Browser

GCA_019633955.1 NCBI Genome Browser

GCA_019733435.1 NCBI Genome Browser

GCA_019915805.1 NCBI Genome Browser

GCA_019915815.1 NCBI Genome Browser

GCA_019915825.1 NCBI Genome Browser

GCA_019915865.1 NCBI Genome Browser

GCA_020026255.1 NCBI Genome Browser

GCA_020091565.1 NCBI Genome Browser

GCA_020097275.1 NCBI Genome Browser

GCA_020256775.1 NCBI Genome Browser

GCA_020256835.1 NCBI Genome Browser

GCA_020256925.1 NCBI Genome Browser

GCA_020257085.1 NCBI Genome Browser

GCA_020257125.1 NCBI Genome Browser

GCA_020297425.1 NCBI Genome Browser

GCA_020297435.1 NCBI Genome Browser

GCA_020297545.1 NCBI Genome Browser

GCA_020297585.1 NCBI Genome Browser

GCA_020538785.1 NCBI Genome Browser

GCA_020539125.1 NCBI Genome Browser

GCA_020558655.1 NCBI Genome Browser

GCA_020558865.1 NCBI Genome Browser

GCA_020809855.1 NCBI Genome Browser

GCA_020859785.1 NCBI Genome Browser

GCA_021202455.1 NCBI Genome Browser

GCA_021359565.1 NCBI Genome Browser

GCA_021359585.1 NCBI Genome Browser

GCA_021532015.1 NCBI Genome Browser

GCA_021619085.1 NCBI Genome Browser

GCA_021633405.1 NCBI Genome Browser

GCA_022713585.1 NCBI Genome Browser

GCA_022714035.1 NCBI Genome Browser

GCA_022714225.1 NCBI Genome Browser

GCA_022714305.1 NCBI Genome Browser

GCA_022716295.1 NCBI Genome Browser

GCA_022716895.1 NCBI Genome Browser

GCA_022717255.1 NCBI Genome Browser

GCA_022718435.1 NCBI Genome Browser

GCA_022719595.1 NCBI Genome Browser

GCA_022719775.1 NCBI Genome Browser

GCA_022720585.1 NCBI Genome Browser

GCA_022721415.1 NCBI Genome Browser

GCA_022722555.1 NCBI Genome Browser

GCA_022723375.1 NCBI Genome Browser

GCA_022724385.1 NCBI Genome Browser

GCA_022724695.1 NCBI Genome Browser

GCA_022725025.1 NCBI Genome Browser

GCA_022725225.1 NCBI Genome Browser

GCA_022726355.1 NCBI Genome Browser

GCA_022727455.1 NCBI Genome Browser

GCA_022727875.1 NCBI Genome Browser

GCA_022728755.1 NCBI Genome Browser

GCA_022731325.1 NCBI Genome Browser

GCA_022732465.1 NCBI Genome Browser

GCA_022733095.1 NCBI Genome Browser

GCA_022733395.1 NCBI Genome Browser

GCA_022733815.1 NCBI Genome Browser

GCA_022734035.1 NCBI Genome Browser

GCA_022734285.1 NCBI Genome Browser

GCA_022734965.1 NCBI Genome Browser

GCA_022735135.1 NCBI Genome Browser

GCA_022737435.1 NCBI Genome Browser

GCA_022737795.1 NCBI Genome Browser

GCA_022737955.1 NCBI Genome Browser

GCA_022738395.1 NCBI Genome Browser

GCA_022738585.1 NCBI Genome Browser

GCA_022739935.1 NCBI Genome Browser

GCA_022741085.1 NCBI Genome Browser

GCA_022741265.1 NCBI Genome Browser

GCA_022741435.1 NCBI Genome Browser

GCA_022741515.1 NCBI Genome Browser

GCA_022741665.1 NCBI Genome Browser

GCA_022742205.1 NCBI Genome Browser

GCA_022743465.1 NCBI Genome Browser

GCA_022744835.1 NCBI Genome Browser

GCA_022744925.1 NCBI Genome Browser

GCA_022745295.1 NCBI Genome Browser

GCA_022745625.1 NCBI Genome Browser

GCA_022746345.1 NCBI Genome Browser

GCA_022746405.1 NCBI Genome Browser

GCA_022746755.1 NCBI Genome Browser

GCA_022747335.1 NCBI Genome Browser

GCA_022780605.1 NCBI Genome Browser

GCA_022782465.1 NCBI Genome Browser

GCA_022832315.1 NCBI Genome Browser

GCA_022832325.1 NCBI Genome Browser

GCA_022832355.1 NCBI Genome Browser

GCA_023274245.1 NCBI Genome Browser

GCA_023274315.1 NCBI Genome Browser

GCA_023274495.1 NCBI Genome Browser

GCA_023274715.1 NCBI Genome Browser

GCA_023274755.1 NCBI Genome Browser

GCA_023274775.1 NCBI Genome Browser

GCA_023274895.1 NCBI Genome Browser

GCA_023275035.1 NCBI Genome Browser

GCA_023276265.1 NCBI Genome Browser

GCA_023276305.1 NCBI Genome Browser

GCA_023276335.1 NCBI Genome Browser

GCA_023653985.1 NCBI Genome Browser

GCA_023654005.1 NCBI Genome Browser

GCA_023654015.1 NCBI Genome Browser

GCA_023654025.1 NCBI Genome Browser

GCA_023654085.1 NCBI Genome Browser

GCA_023654095.1 NCBI Genome Browser

GCA_023654105.1 NCBI Genome Browser

GCA_023654145.1 NCBI Genome Browser

GCA_023654165.1 NCBI Genome Browser

GCA_023654185.1 NCBI Genome Browser

GCA_023654195.1 NCBI Genome Browser

GCA_023654205.1 NCBI Genome Browser

GCA_023654225.1 NCBI Genome Browser

GCA_023654265.1 NCBI Genome Browser

GCA_023654285.1 NCBI Genome Browser

GCA_023654295.1 NCBI Genome Browser

GCA_023654305.1 NCBI Genome Browser

GCA_023654345.1 NCBI Genome Browser

GCA_023654365.1 NCBI Genome Browser

GCA_023654395.1 NCBI Genome Browser

GCA_023654425.1 NCBI Genome Browser

GCA_023654445.1 NCBI Genome Browser

GCA_023654465.1 NCBI Genome Browser

GCA_023654475.1 NCBI Genome Browser

GCA_023654505.1 NCBI Genome Browser

GCA_023654525.1 NCBI Genome Browser

GCA_023654535.1 NCBI Genome Browser

GCA_023654565.1 NCBI Genome Browser

GCA_023654575.1 NCBI Genome Browser

GCA_023654605.1 NCBI Genome Browser

GCA_023654625.1 NCBI Genome Browser

GCA_023654635.1 NCBI Genome Browser

GCA_023654665.1 NCBI Genome Browser

GCA_023654685.1 NCBI Genome Browser

GCA_023654705.1 NCBI Genome Browser

GCA_023654725.1 NCBI Genome Browser

GCA_023654745.1 NCBI Genome Browser

GCA_023654765.1 NCBI Genome Browser

GCA_023654775.1 NCBI Genome Browser

GCA_023702735.1 NCBI Genome Browser

GCA_023894135.1 NCBI Genome Browser

GCA_023958435.1 NCBI Genome Browser

GCA_024007805.1 NCBI Genome Browser

GCA_900445515.1 NCBI Genome Browser

GCA_900624785.1 NCBI Genome Browser

GCA_901212445.1 NCBI Genome Browser

GCA_902364655.1 NCBI Genome Browser

GCA_902374115.1 NCBI Genome Browser

GCA_903181445.1 NCBI Genome Browser

GCA_905188165.1 NCBI Genome Browser

GCA_905207945.1 NCBI Genome Browser

SRR12280270 PRJNA646575

SRR12280314 PRJNA646575

SRR12280315 PRJNA646575

SRR12280316 PRJNA646575

SRR12280336 PRJNA646575

SRR12280416 PRJNA646575

SRR12280417 PRJNA646575

SRR12280492 PRJNA646575

SRR12280493 PRJNA646575

SRR12280496 PRJNA646575

SRR12280497 PRJNA646575

SRR12280498 PRJNA646575

SRR12280499 PRJNA646575

SRR12280500 PRJNA646575

SRR12280501 PRJNA646575

SRR12280503 PRJNA646575

SRR12280504 PRJNA646575

SRR12280511 PRJNA646575

SRR12280522 PRJNA646575

SRR12280549 PRJNA646575

SRR12280561 PRJNA646575

SRR12280572 PRJNA646575

SRR12280583 PRJNA646575

SRR12280589 PRJNA646575

SRR12280591 PRJNA646575

SRR12280608 PRJNA646575

SRR12280619 PRJNA646575

SRR12280632 PRJNA646575

SRR12280635 PRJNA646575

SRR12280637 PRJNA646575

SRR12280639 PRJNA646575

SRR12280641 PRJNA646575

SRR12280642 PRJNA646575

SRR12280643 PRJNA646575

SRR12280645 PRJNA646575

SRR12280646 PRJNA646575

SRR12280647 PRJNA646575

SRR12280648 PRJNA646575

SRR12280649 PRJNA646575

SRR12280650 PRJNA646575

SRR12280651 PRJNA646575

SRR12280658 PRJNA646575

SRR13269111 PRJNA646575

SRR13269122 PRJNA646575

SRR13269147 PRJNA646575

SRR13269148 PRJNA646575

SRR13269149 PRJNA646575

SRR13269150 PRJNA646575

SRR13269152 PRJNA646575

SRR13269154 PRJNA646575

SRR13269155 PRJNA646575

SRR13269156 PRJNA646575

SRR13269157 PRJNA646575

SRR13269158 PRJNA646575

SRR13269159 PRJNA646575

SRR13269161 PRJNA646575

SRR13269162 PRJNA646575

SRR13269163 PRJNA646575

SRR13269164 PRJNA646575

SRR13269165 PRJNA646575

SRR13269166 PRJNA646575

SRR13269167 PRJNA646575

SRR13269178 PRJNA646575

SRR13269194 PRJNA646575

SRR13269204 PRJNA646575

SRR13269207 PRJNA646575

SRR13269209 PRJNA646575

SRR13269219 PRJNA646575

SRR13269220 PRJNA646575

SRR13269221 PRJNA646575

SRR13269222 PRJNA646575

SRR13269223 PRJNA646575

SRR13269224 PRJNA646575

SRR13269245 PRJNA646575

SRR15283685 PRJNA745162

SRR15283687 PRJNA745162

SRR15283688 PRJNA745162

SRR15283691 PRJNA745162

SRR15283695 PRJNA745162

SRR15283697 PRJNA745162

SRR15283699 PRJNA745162

SRR15283700 PRJNA745162

SRR15283701 PRJNA745162

SRR15283704 PRJNA745162

SRR15283705 PRJNA745162

SRR15283706 PRJNA745162

SRR15283707 PRJNA745162

SRR15283708 PRJNA745162

SRR15283713 PRJNA745162

SRR15283714 PRJNA745162

SRR15283717 PRJNA745162

SRR15283720 PRJNA745162

SRR15283722 PRJNA745162

SRR15283724 PRJNA745162

SRR15283728 PRJNA745162

SRR15283731 PRJNA745162

SRR15283737 PRJNA745162

SRR15283738 PRJNA745162

SRR15283742 PRJNA745162

SRR15283745 PRJNA745162

SRR15283746 PRJNA745162

SRR15283747 PRJNA745162

SRR15283748 PRJNA745162

SRR15283749 PRJNA745162

SRR15283750 PRJNA745162

SRR15283752 PRJNA745162

SRR15283753 PRJNA745162

SRR15283754 PRJNA745162

SRR15283755 PRJNA745162

SRR15283756 PRJNA745162

SRR15283757 PRJNA745162

SRR15283758 PRJNA745162

SRR15283762 PRJNA745162

SRR15283763 PRJNA745162

SRR15283765 PRJNA745162

SRR15283766 PRJNA745162

SRR15283767 PRJNA745162

SRR15283770 PRJNA745162

SRR15283771 PRJNA745162

SRR15283773 PRJNA745162

SRR15283774 PRJNA745162

SRR15283775 PRJNA745162

SRR15283776 PRJNA745162

SRR15283777 PRJNA745162

SRR15283778 PRJNA745162

SRR15283779 PRJNA745162

SRR15283780 PRJNA745162

SRR15283781 PRJNA745162

SRR15283782 PRJNA745162

SRR15283784 PRJNA745162

SRR15283785 PRJNA745162

SRR15283787 PRJNA745162

SRR15283788 PRJNA745162

SRR15283789 PRJNA745162

SRR15283790 PRJNA745162

SRR15283791 PRJNA745162

SRR15283795 PRJNA745162

SRR15283815 PRJNA745162

SRR15283820 PRJNA745162

SRR15283822 PRJNA745162

SRR15283825 PRJNA745162

SRR15283826 PRJNA745162

SRR15283828 PRJNA745162

SRR15283834 PRJNA745162

SRR15283837 PRJNA745162

SRR15283838 PRJNA745162

SRR15283839 PRJNA745162

SRR15283841 PRJNA745162

SRR15283842 PRJNA745162

SRR15283844 PRJNA745162

SRR15283848 PRJNA745162

SRR15283850 PRJNA745162

SRR15283852 PRJNA745162

SRR15283853 PRJNA745162

SRR15283854 PRJNA745162

SRR15283861 PRJNA745162

SRR15283864 PRJNA745162

SRR15283865 PRJNA745162

SRR15283867 PRJNA745162

SRR15283868 PRJNA745162

SRR15283869 PRJNA745162

SRR15283871 PRJNA745162

SRR15283873 PRJNA745162

SRR15283876 PRJNA745162

SRR15283885 PRJNA745162

SRR15283887 PRJNA745162

SRR15283888 PRJNA745162

SRR15283890 PRJNA745162

SRR15283891 PRJNA745162

SRR15283892 PRJNA745162

SRR15283894 PRJNA745162

SRR15283896 PRJNA745162

SRR9205642 PRJNA544527

SRR9205647 PRJNA544527

SRR9205878 PRJNA544527

SRR9206013 PRJNA544527

SRR9206014 PRJNA544527

SRR9206102 PRJNA544527

SRR9206103 PRJNA544527

SRR9206104 PRJNA544527

SRR9206108 PRJNA544527

SRR9206109 PRJNA544527

SRR9206239 PRJNA544527

SRR9206389 PRJNA544527

SRR9206390 PRJNA544527

SRR9206395 PRJNA544527

SRR9206396 PRJNA544527

Table 2: Accession numbers for all public isolate whole genome sequences used in this study.
