# Supplementary material for: Pangenome comparison of Bacteroides fragilis genomospecies unveils genetic diversity and ecological insights
Source: mSystems. 2024 Jun 27;9(7):e00516-24. doi: 10.1128/msystems.00516-24 (PMC11265264; doi:10.1128/msystems.00516-24)
Supplement: Table S3 — Genes differentially prevalent between division I and II strains. [file msystems.00516-24-s0004.docx]

Gene D1_Percentage D2_Percentage Percentage_Difference D2_lg D1_lg lfc Annotation No..isolates Product RefSeq UniParc UniRef

group_15977 100.8194946 13.14285714 87.67663744 2.575878428 4.613331736 2.037453308 ABC transporter permease;FtsX-like permease family protein 576 ABC transporter permease WP_005795278.1 UPI0002132C52 UniRef100_A0A1C0WW19,UniRef50_A0A1C0WW19,UniRef90_A0A1C0WW19

group_15170 100.6389892 13.14285714 87.49613203 2.575878428 4.611539749 2.035661321 hypothetical protein;ABC transporter permease 575 ABC transporter permease WP_005800748.1 UPI0001DDE1ED UniRef100_F7LNB2,UniRef50_Q64W68,UniRef90_Q64W68

group_9995 101 9.571428571 91.42857143 2.25878247 4.615120517 2.356338047 hypothetical protein;succinate--CoA ligase subunit alpha 572 succinate--CoA ligase subunit alpha WP_005777698.1 UPI0001B4AE90 UniRef100_A0A2K9GXE9,UniRef50_A0A0N4V8W0,UniRef90_A0A0K6BU24

group_8613 101 9.571428571 91.42857143 2.25878247 4.615120517 2.356338047 DNA mismatch repair protein MutT 572 DNA mismatch repair protein MutT WP_005787639.1 UPI000043E84C UniRef100_A0A149NKK0,UniRef50_K9E9Y7,UniRef90_A0A149NKK0

group_6934 101 9.571428571 91.42857143 2.25878247 4.615120517 2.356338047 ADP-forming succinate--CoA ligase subunit beta;Succinate--CoA ligase subunit beta;hypothetical protein 572 ADP-forming succinate--CoA ligase subunit beta UniRef50_O28097,UniRef90_Q5LCW4

group_2418 101 9.571428571 91.42857143 2.25878247 4.615120517 2.356338047 bifunctional hydroxymethylpyrimidine kinase/phosphomethylpyrimidine kinase;hypothetical protein 572 bifunctional hydroxymethylpyrimidine kinase/phosphomethylpyrimidine kinase WP_005777705.1 UPI0001B4AE92 UniRef100_A0A2K9H5T9,UniRef50_A0A0N7IA57,UniRef90_A0A149NKH2

group_5143 100.8194946 9.571428571 91.24806601 2.25878247 4.613331736 2.354549265 FGGY-C domain-containing protein;hypothetical protein;carbohydrate kinase;Xylulokinase;FGGY-N domain-containing protein 571 carbohydrate kinase UniRef50_A0A2X2JKC4,UniRef90_Q5LCW0

group_1992 100.8194946 7.428571429 93.39092316 2.00533357 4.613331736 2.607998166 hypothetical protein 568 hypothetical protein UniRef50_Q5LHH7,UniRef90_Q5LHH7

tpiA 100.0974729 10.28571429 89.81175864 2.33075597 4.60614444 2.275388471 triose-phosphate isomerase 568 triose-phosphate isomerase WP_005791115.1 UPI000043EED5 UniRef100_Q5L923,UniRef50_Q8A0U2,UniRef90_Q8A0U2

group_1967 101 6 95 1.791759469 4.615120517 2.823361048 SAM-dependent methyltransferase 567 SAM-dependent methyltransferase UniRef50_K0XGK5,UniRef90_A0A0K6BQH6

group_1604 99.19494585 13.14285714 86.05208871 2.575878428 4.597087064 2.021208636 hypothetical protein 567 hypothetical protein UniRef50_A0A016E273,UniRef90_A0A016E273

group_7441 101 4.571428571 96.42857143 1.519825754 4.615120517 3.095294763 glycosyl hydrolase family 43;Extracellular exo-alpha-(1->5)-L-arabinofuranosidase;hypothetical protein;family 43 glycosylhydrolase 565 glycosyl hydrolase family 43 WP_195316993.1 UPI00189A57C5 UniRef100_UPI00189A57C5,UniRef50_A0A1V5YTH8,UniRef90_A0A0K6BNV7

group_6568 101 4.571428571 96.42857143 1.519825754 4.615120517 3.095294763 DUF3256 domain-containing protein 565 DUF3256 domain-containing protein WP_230583504.1 UPI001E50EA17 UniRef100_UPI001E50EA17,UniRef50_A0A0N7IAN6,UniRef90_Q64XU8

group_4387 101 4.571428571 96.42857143 1.519825754 4.615120517 3.095294763 hypothetical protein 565 hypothetical protein WP_005788103.1 UPI00004E2575 UniRef100_A0A396ELV6,UniRef50_I9S274,UniRef90_A0A017N8S1

group_2977 101 4.571428571 96.42857143 1.519825754 4.615120517 3.095294763 TSCPD domain-containing protein;hypothetical protein 565 TSCPD domain-containing protein WP_195595310.1 UPI00189D45D0 UniRef100_UPI00189D45D0,UniRef50_B8I191,UniRef90_A0A0E2B0A0

group_15571 100.4584838 5.285714286 95.17276947 1.665007764 4.609744545 2.944736782 hypothetical protein;sulfatase 563 sulfatase WP_032557423.1 UPI000453145A UniRef100_A0A3E5I2C3,UniRef50_A0A517M895,UniRef90_Q5L931

pheT 100.8194946 3.857142857 96.96235173 1.349926717 4.613331736 3.263405019 Phenylalanine--tRNA ligase beta subunit 563 Phenylalanine--tRNA ligase beta subunit WP_005788108.1 UPI00004E2576 UniRef100_Q5LC76,UniRef50_Q5LC76,UniRef90_Q5LC76

group_2417 99.55595668 8.857142857 90.69881382 2.181224236 4.600719865 2.419495629 hypothetical protein 563 hypothetical protein

group_1646 101 3.142857143 97.85714286 1.145132304 4.615120517 3.469988213 hypothetical protein;Tryptophan--tRNA ligase 563 Tryptophan--tRNA ligase WP_130071278.1 UPI0010201924 UniRef100_A0A642F087,UniRef90_J9DD46

group_14508 100.6389892 3.857142857 96.78184631 1.349926717 4.611539749 3.261613032 hypothetical protein 562 hypothetical protein WP_005786243.1 UPI000043E561 UniRef100_D1JKE9,UniRef50_D1JKE9,UniRef90_D1JKE9

group_11812 101 2.428571429 98.57142857 0.887303195 4.615120517 3.727817322 alpha/beta fold hydrolase;Hydrolase-4 domain-containing protein;Serine aminopeptidase S33 562 Serine aminopeptidase, S33 UniRef50_Q5LH43,UniRef90_Q5LH43

group_7437 101 2.428571429 98.57142857 0.887303195 4.615120517 3.727817322 hypothetical protein 562 hypothetical protein

group_4759 101 2.428571429 98.57142857 0.887303195 4.615120517 3.727817322 pyridine nucleotide-disulfide oxidoreductase;hypothetical protein;Pyridine nucleotide-disulfide oxidoreductase;NAD(FAD)-dependent dehydrogenase 562 pyridine nucleotide-disulfide oxidoreductase WP_005784478.1 UPI000043E1EF UniRef100_D1JRI6,UniRef50_Q5LHV0,UniRef90_Q5LHV0

cobS 101 2.428571429 98.57142857 0.887303195 4.615120517 3.727817322 adenosylcobinamide-GDP ribazoletransferase;hypothetical protein 562 adenosylcobinamide-GDP ribazoletransferase UniRef50_F3PFA5,UniRef90_Q5LCE5

group_991 101 2.428571429 98.57142857 0.887303195 4.615120517 3.727817322 MarR family transcriptional regulator 562 MarR family transcriptional regulator UniRef50_I8XS19,UniRef90_A0A0K6BQN7

group_16039 101 1.714285714 99.28571429 0.538996501 4.615120517 4.076124016 hypothetical protein 561 hypothetical protein UPI0002133312 UniRef100_A0A2M9UTH9,UniRef50_A0A2M9UTH9,UniRef90_A0A2M9UTH9

group_14627 100.8194946 2.428571429 98.39092316 0.887303195 4.613331736 3.726028541 SNARE associated Golgi protein 561 SNARE associated Golgi protein WP_005783683.1 UPI000043E005 UniRef100_A0A0K6BND2,UniRef50_A0A0P0FWC7,UniRef90_A0A0K6BND2

group_14578 101 1.714285714 99.28571429 0.538996501 4.615120517 4.076124016 glycosyl hydrolase;hypothetical protein 561 glycosyl hydrolase WP_005797938.1 UPI000043EDEE UniRef100_A0A2M9UWX9,UniRef50_A0A1H7T6E1,UniRef90_A0A2M9UWX9

group_14512 101 1.714285714 99.28571429 0.538996501 4.615120517 4.076124016 ABC transporter permease;hypothetical protein 561 ABC transporter permease WP_005786264.1 UPI0001DDE1E9 UniRef100_F7LNA3,UniRef50_Q5LFF4,UniRef90_Q5LFF4

group_10954 101 1.714285714 99.28571429 0.538996501 4.615120517 4.076124016 hypothetical protein;Outer membrane efflux protein;TolC family protein 561 Outer membrane efflux protein WP_005798684.1 UPI000043EB89 UniRef100_A0A0K6BWY4,UniRef50_R5X1U2,UniRef90_A0A0K6BWY4

group_9763 101 1.714285714 99.28571429 0.538996501 4.615120517 4.076124016 phosphatidylserine decarboxylase family protein 561 phosphatidylserine decarboxylase family protein WP_005784739.1 UPI000043E272 UniRef100_Q5LHJ3,UniRef50_Q8A5K8,UniRef90_Q5LHJ3

group_7387 101 1.714285714 99.28571429 0.538996501 4.615120517 4.076124016 hypothetical protein 561 hypothetical protein UniRef50_D1JQU7,UniRef90_D1JQU7

group_7094 101 1.714285714 99.28571429 0.538996501 4.615120517 4.076124016 hypothetical protein;two-component system response regulator 561 hypothetical protein

group_6009 101 1.714285714 99.28571429 0.538996501 4.615120517 4.076124016 Sensor histidine kinase;Histidine kinase domain-containing protein;histidine kinase;ATP-binding protein;hypothetical protein;His Kinase A domain protein;GAF domain protein 561 histidine kinase WP_130070869.1 UPI0010202D3D UniRef100_A0A642F9D3,UniRef50_A0A2M9V7C3,UniRef90_A0A2M9V7C3

group_5914 101 1.714285714 99.28571429 0.538996501 4.615120517 4.076124016 hypothetical protein;beta-N-acetylglucosaminidase;serine hydrolase;Beta-N-acetylhexosaminidase domain protein 561 beta-N-acetylglucosaminidase UniRef50_F3ZSA4,UniRef90_A0A7U8QCM5

group_5181 101 1.714285714 99.28571429 0.538996501 4.615120517 4.076124016 AsmA-like C-terminal region;outer membrane assembly protein;hypothetical protein;AsmA domain-containing protein 561 outer membrane assembly protein WP_130070925.1 UPI001021907D UniRef100_A0A642HWN6,UniRef50_I9R8B7,UniRef90_Q5LIK6

group_4359 101 1.714285714 99.28571429 0.538996501 4.615120517 4.076124016 hypothetical protein 561 hypothetical protein UniRef50_A0A0K6BY82,UniRef90_A0A0K6BY82

lpxA 101 1.714285714 99.28571429 0.538996501 4.615120517 4.076124016 hypothetical protein;Acyl-(Acyl-carrier-protein)--UDP-N-acetylglucosamine O-acyltransferase;acyl-ACP--UDP-N-acetylglucosamine O-acyltransferase 561 acyl-ACP--UDP-N-acetylglucosamine O-acyltransferase WP_005783787.1 UPI000043E06D UniRef100_A0A0K6BNF0,UniRef50_A0A0P0F0Z8,UniRef90_A0A0K6BNF0

group_4100 101 1.714285714 99.28571429 0.538996501 4.615120517 4.076124016 NAD-dependent protein deacylase 561 NAD-dependent protein deacylase UniRef50_Q8A3H9,UniRef90_A0A2M9V2R3

group_4029 101 1.714285714 99.28571429 0.538996501 4.615120517 4.076124016 hypothetical protein;UDP-N-acetylmuramate dehydrogenase;UDP-N-acetylenolpyruvoylglucosamine reductase 561 UDP-N-acetylmuramate dehydrogenase WP_032580148.1 UPI00044B561A UniRef100_A0A2M9UPH1,UniRef50_Q5LBG5,UniRef90_Q5LBG5

group_3997 101 1.714285714 99.28571429 0.538996501 4.615120517 4.076124016 hypothetical protein;Isopentenyl-diphosphate delta-isomerase 561 Isopentenyl-diphosphate delta-isomerase WP_005785267.1 UPI000043E37D UniRef100_A0A0K6BQZ1,UniRef50_W4PHR9,UniRef90_A0A0K6BQZ1

group_3726 101 1.714285714 99.28571429 0.538996501 4.615120517 4.076124016 Zeta-toxin domain-containing protein 561 Zeta-toxin domain-containing protein WP_130070848.1 UPI00101F1DF2 UniRef100_A0A642F880,UniRef50_Q64U60,UniRef90_Q64U60

group_2598 101 1.714285714 99.28571429 0.538996501 4.615120517 4.076124016 hypothetical protein;ABC transporter permease 561 ABC transporter permease UniRef50_A0A396MEZ2,UniRef90_F7LR61

group_2450 101 1.714285714 99.28571429 0.538996501 4.615120517 4.076124016 hypothetical protein;glycosyl transferase 561 glycosyl transferase WP_032562005.1 UPI0005173B84 UniRef100_UPI0005173B84,UniRef50_A0A5J4Q1H8,UniRef90_Q5L7H9

group_2348 101 1.714285714 99.28571429 0.538996501 4.615120517 4.076124016 hypothetical protein;Carbohydrate-binding family 9 561 Carbohydrate-binding family 9 WP_009291346.1 UPI00021329F2 UniRef100_F7LJN1,UniRef50_E5WW92,UniRef90_D1JQI1

group_1997 101 1.714285714 99.28571429 0.538996501 4.615120517 4.076124016 Metal-dependent hydrolases;MBL fold metallo-hydrolase;hypothetical protein;Lactamase-B domain-containing protein 561 MBL fold metallo-hydrolase WP_005788758.1 UPI00004E261E UniRef100_D1JTH4,UniRef50_A0A120A243,UniRef90_D1JTH4

group_1985 100.8194946 2.428571429 98.39092316 0.887303195 4.613331736 3.726028541 hypothetical protein 561 hypothetical protein UniRef50_U6R7Q4,UniRef90_A0A3E5ICM4

group_1722 101 1.714285714 99.28571429 0.538996501 4.615120517 4.076124016 hypothetical protein;RNA polymerase sigma-70 factor 561 RNA polymerase sigma-70 factor WP_005798225.1 UPI000043EE7F UniRef100_A0A0E2AM90,UniRef50_A0A0E2AM90,UniRef90_A0A0E2AM90

group_1169 101 1.714285714 99.28571429 0.538996501 4.615120517 4.076124016 hypothetical protein 561 hypothetical protein WP_005788365.1 UPI00004E25C1 UniRef100_A0A149N4W1,UniRef50_E6SRR2,UniRef90_Q64ST9

group_1013 101 1.714285714 99.28571429 0.538996501 4.615120517 4.076124016 hypothetical protein 561 hypothetical protein UniRef50_A0A0N7IAW9,UniRef90_A0A1C0X304

group_928 101 1.714285714 99.28571429 0.538996501 4.615120517 4.076124016 metallophosphatase 561 metallophosphatase WP_005801566.1 UPI000043E1E5 UniRef100_A0A149N0U7,UniRef50_K9EMP0,UniRef90_K1FZ83

group_16600 101 1 100 0 4.615120517 4.615120517 DNA-binding protein 560 DNA-binding protein WP_005795912.1 UPI000043E3CA UniRef100_A0A0K6BQQ1,UniRef50_K1GVA4,UniRef90_K1GVA4

group_14773 101 1 100 0 4.615120517 4.615120517 hypothetical protein 560 hypothetical protein WP_005786021.1 UPI0002132414 UniRef100_E1WRT9,UniRef50_Q5LFS8,UniRef90_Q5LFS8

yidD 101 1 100 0 4.615120517 4.615120517 membrane protein insertion efficiency factor YidD 560 membrane protein insertion efficiency factor YidD WP_005779696.1 UPI000043DFF5 UniRef100_Q5LJ21,UniRef50_B7GK26,UniRef90_Q5LJ21

group_14629 100.8194946 1.714285714 99.10520887 0.538996501 4.613331736 4.074335235 Periplasmic solute binding family protein;hypothetical protein 560 Periplasmic solute binding family protein UniRef50_R6JBJ1,UniRef90_A0A015W6E7

group_14534 101 1 100 0 4.615120517 4.615120517 hypothetical protein 560 hypothetical protein WP_005796358.1 UPI000043E222 UniRef100_A0A0K6BPZ7,UniRef50_A0A0K6BPZ7,UniRef90_A0A0K6BPZ7

group_12519 101 1 100 0 4.615120517 4.615120517 hypothetical protein 560 hypothetical protein UniRef50_I3YI70,UniRef90_F7LKW7

group_7443 101 1 100 0 4.615120517 4.615120517 hypothetical protein 560 hypothetical protein

group_7429 101 1 100 0 4.615120517 4.615120517 Putative exported protein;hypothetical protein 560 hypothetical protein WP_005791934.1 UPI000043F0D9 UniRef100_A0A0K6C0R4,UniRef50_A0A173TEE2,UniRef90_A0A0K6C0R4

group_7312 100.8194946 1.714285714 99.10520887 0.538996501 4.613331736 4.074335235 hypothetical protein;Precorrin-4 C11-methyltransferase;precorrin-4 C(11)-methyltransferase;Cobalt-precorrin-4 C(11)-methyltransferase 560 precorrin-4 C(11)-methyltransferase UniRef50_A0A0P0M3U2,UniRef90_A0A1C0WHU7

group_7107 101 1 100 0 4.615120517 4.615120517 hypothetical protein 560 hypothetical protein WP_008769028.1 UPI000043E9E5 UniRef100_A0A414ZYR5,UniRef50_A0A069CXQ2,UniRef90_A0A414ZYR5

group_6004 101 1 100 0 4.615120517 4.615120517 hypothetical protein;sensor histidine kinase 560 sensor histidine kinase WP_130070924.1 UPI001022687A UniRef100_A0A642FKT6,UniRef50_A0A0P0GHZ8,UniRef90_K1G5F3

mgtA 101 1 100 0 4.615120517 4.615120517 Magnesium transporting ATPase P-type 1;hypothetical protein;Magnesium-transporting ATPase MgtA;cation transporting ATPase C-terminal domain-containing protein;HAD-IC family P-type ATPase;magnesium-translocating P-type ATPase;Magnesium-transporting ATPase P-type 1 560 magnesium-translocating P-type ATPase WP_130070918.1 UPI00101EA3A0 UniRef100_A0A642HC61,UniRef50_A0A445NJV8,UniRef90_E1WK47

group_4873 101 1 100 0 4.615120517 4.615120517 exo-alpha-sialidase;hypothetical protein;glycoside hydrolase 560 glycoside hydrolase WP_182115760.1 UPI0015F39723 UniRef100_UPI001D020D8C,UniRef50_Q64U61,UniRef90_Q64U61

group_4861 101 1 100 0 4.615120517 4.615120517 hypothetical protein;lipocalin family protein;Lipocalin-like domain-containing protein;Lipocalin-like family protein 560 Lipocalin-like domain-containing protein UniRef50_R7DIB9,UniRef90_D1JRK2

group_4831 101 1 100 0 4.615120517 4.615120517 PA14 domain-containing protein;hypothetical protein;beta-glucosidase;glycoside hydrolase family 3 C-terminal domain-containing protein 560 beta-glucosidase UniRef50_K0WU03,UniRef90_Q5LIH3

group_4772 101 1 100 0 4.615120517 4.615120517 hypothetical protein 560 hypothetical protein UniRef50_A0A7T4QP59,UniRef90_Q64Z01

group_4752 101 1 100 0 4.615120517 4.615120517 hypothetical protein;Ribonucleoside-diphosphate reductase;Ribonucleoside-diphosphate reductase subunit alpha;ribonucleoside-diphosphate reductase subunit alpha 560 ribonucleoside-diphosphate reductase subunit alpha WP_005788368.1 UPI000043E9F6 UniRef100_A0A0K6BVY6,UniRef50_O83972,UniRef90_A0A0K6BVY6

group_4714 101 1 100 0 4.615120517 4.615120517 hypothetical protein;Ligand-binding sensor domain-containing protein;DNA-binding response regulator;hybrid sensor histidine kinase/response regulator;Sensor histidine kinase TodS 560 hybrid sensor histidine kinase/response regulator WP_032568842.1 UPI00044CF49A UniRef100_A0A642EXY2,UniRef50_A0A853PN24,UniRef90_A0A853PN24

group_4687 101 1 100 0 4.615120517 4.615120517 hypothetical protein;beta-glucanase 560 beta-glucanase UniRef50_I9VYL0,UniRef90_A0A0E2ATR9

group_4670 101 1 100 0 4.615120517 4.615120517 Histidine kinase;two-component sensor histidine kinase 560 two-component sensor histidine kinase WP_008657969.1 UPI000043E083 UniRef100_A0A1C0WN53,UniRef50_A0A1C0WN53,UniRef90_A0A1C0WN53

group_4603 101 1 100 0 4.615120517 4.615120517 potassium transporter;hypothetical protein;Cation transport protein;Trk system potassium uptake protein TrkH domain protein 560 potassium transporter WP_115472484.1 UPI000E1E12FB UniRef100_A0A5M5P6U5,UniRef50_A0A0P0FUG7,UniRef90_A0A380YNH1

group_4525 101 1 100 0 4.615120517 4.615120517 HGSNAT-cat domain-containing protein;transporter 560 transporter WP_005813741.1 UPI0002808933 UniRef100_K1G529,UniRef50_F5IST8,UniRef90_K1G529

group_4265 101 1 100 0 4.615120517 4.615120517 hypothetical protein;Bacteriocin-protection YdeI or OmpD-Associated 560 Bacteriocin-protection, YdeI or OmpD-Associated WP_005788988.1 UPI000043EBA8 UniRef100_A0A0K6BWQ6,UniRef50_A0A150ACC8,UniRef90_A0A0K6BWQ6

group_3854 101 1 100 0 4.615120517 4.615120517 hypothetical protein;6-bladed beta-propeller 560 6-bladed beta-propeller WP_009291560.1 UPI00021322E6 UniRef100_F7LKR6,UniRef50_E1WL90,UniRef90_E1WL90

group_3772 101 1 100 0 4.615120517 4.615120517 hypothetical protein 560 hypothetical protein WP_022347732.1 UPI0003351AAA UniRef100_R6YYG0,UniRef50_Q5LAS5,UniRef90_Q5LAS5

group_3767 101 1 100 0 4.615120517 4.615120517 urea transporter;hypothetical protein;Urea transporter 560 urea transporter WP_032529538.1 UPI000446BF90 UniRef100_A0A5C6HEY7,UniRef50_A0A108T381,UniRef90_Q5LE30

group_3398 101 1 100 0 4.615120517 4.615120517 hypothetical protein;Alpha-L-rhamnosidase;alpha-rhamnosidase 560 alpha-rhamnosidase WP_130070969.1 UPI001020C166 UniRef100_A0A642F738,UniRef50_A0A355FJY7,UniRef90_A0A380YU69

group_3236 101 1 100 0 4.615120517 4.615120517 ribonucleotide-diphosphate reductase subunit beta;hypothetical protein 560 ribonucleotide-diphosphate reductase subunit beta WP_005788370.1 UPI00000B9D68 UniRef100_Q93LD8,UniRef50_O83092,UniRef90_Q5LBV8

group_3203 101 1 100 0 4.615120517 4.615120517 AraC family transcriptional regulator;GyrI-like domain-containing protein 560 AraC family transcriptional regulator UniRef50_A0A2M9V9W6,UniRef90_A0A2M9V9W6

ftcD 100.8194946 1.714285714 99.10520887 0.538996501 4.613331736 4.074335235 Glutamate formiminotransferase;hypothetical protein;glutamate formimidoyltransferase 560 glutamate formimidoyltransferase WP_065763334.1 UPI0008119BA5 UniRef100_A0A642HEJ0,UniRef50_A6TVD7,UniRef90_Q5L8E3

group_2308 101 1 100 0 4.615120517 4.615120517 Acyl-transf-3 domain-containing protein;hypothetical protein;acyltransferase 560 acyltransferase UniRef50_K9E9U8,UniRef90_A0A0E2AUB8

holB 100.8194946 1.714285714 99.10520887 0.538996501 4.613331736 4.074335235 hypothetical protein;DNA polymerase III delta prime subunit;DNA polymerase III subunit delta 560 DNA polymerase III subunit delta WP_009293331.1 UPI0002133336 UniRef100_F7LWY3,UniRef50_A0A174H5G4,UniRef90_A0A2M9V3D5

group_1756 101 1 100 0 4.615120517 4.615120517 hypothetical protein 560 hypothetical protein UniRef50_F7LVR7,UniRef90_F7LVR7

group_1611 101 1 100 0 4.615120517 4.615120517 hypothetical protein 560 hypothetical protein UniRef50_Q64ZU7,UniRef90_Q64ZU7

group_1391 101 1 100 0 4.615120517 4.615120517 four helix bundle protein;Four helix bundle family protein 560 four helix bundle protein UniRef50_Q5LB61,UniRef90_Q5LB61

group_1154 101 1 100 0 4.615120517 4.615120517 hypothetical protein 560 hypothetical protein UPI0002690548 UniRef100_I9VJM1,UniRef50_A0A0E2AMR3,UniRef90_A0A0E2AMR3

group_1120 101 1 100 0 4.615120517 4.615120517 two-component sensor histidine kinase;Non-motile and phage-resistance protein 560 two-component sensor histidine kinase WP_130070923.1 UPI0010212BC7 UniRef100_A0A642HIA2,UniRef50_K9EM75,UniRef90_A0A2M9V9J8

group_15982 100.8194946 1 99.81949458 0 4.613331736 4.613331736 hypothetical protein 559 hypothetical protein UPI0002133033 UniRef100_A0A0E2AW44,UniRef50_A0A0E2AW44,UniRef90_A0A0E2AW44

group_14736 100.8194946 1 99.81949458 0 4.613331736 4.613331736 DUF2807 domain-containing protein;hypothetical protein 559 DUF2807 domain-containing protein UPI001230C704 UniRef100_A0A642L8E2,UniRef50_I9BKV9,UniRef90_I9BKV9

group_14658 100.8194946 1 99.81949458 0 4.613331736 4.613331736 hypothetical protein 559 hypothetical protein UPI00021322C8 UniRef100_A0A0E2AWA2,UniRef50_A0A0E2AWA2,UniRef90_A0A0E2AWA2

group_14552 100.8194946 1 99.81949458 0 4.613331736 4.613331736 hypothetical protein 559 hypothetical protein UPI0002ED4EF7 UniRef100_A0A015Y4X7,UniRef50_A0A015Y4X7,UniRef90_A0A015Y4X7

group_14505 100.8194946 1 99.81949458 0 4.613331736 4.613331736 hypothetical protein 559 hypothetical protein UPI00004E221A UniRef100_F7LML5,UniRef50_F7LML5,UniRef90_F7LML5

group_7467 100.8194946 1 99.81949458 0 4.613331736 4.613331736 glucosamine-fructose-6-phosphate aminotransferase 559 glucosamine-fructose-6-phosphate aminotransferase UniRef50_A0A2M9V678,UniRef90_A0A2M9V678

group_7015 100.8194946 1 99.81949458 0 4.613331736 4.613331736 hypothetical protein 559 hypothetical protein WP_005800608.1 UPI000268F881 UniRef100_I9BLB7,UniRef50_A0A0E2AS50,UniRef90_A0A0E2AS50

group_5183 100.8194946 1 99.81949458 0 4.613331736 4.613331736 hypothetical protein;Bacterial alpha-L-rhamnosidase family protein;family 78 glycoside hydrolase catalytic domain;alpha-rhamnosidase 559 alpha-rhamnosidase WP_130070940.1 UPI001022301E UniRef100_A0A642F6C3,UniRef50_A0A396MCA9,UniRef90_Q5LID7

group_5072 100.8194946 1 99.81949458 0 4.613331736 4.613331736 Crp/Fnr family transcriptional regulator;Cyclic nucleotide-binding domain 559 Crp/Fnr family transcriptional regulator UniRef50_K9ETP4,UniRef90_R6E3D4

group_4735 100.8194946 1 99.81949458 0 4.613331736 4.613331736 hypothetical protein;Carbohydrate-selective porin OprB 559 Carbohydrate-selective porin OprB UniRef50_Q5LIS8,UniRef90_Q5LIS8

group_4676 100.8194946 1 99.81949458 0 4.613331736 4.613331736 HU family DNA-binding protein;DNA-binding protein 559 DNA-binding protein UniRef50_I9V6A7,UniRef90_I9V6A7

group_4475 100.4584838 2.428571429 98.02991233 0.887303195 4.609744545 3.72244135 hypothetical protein 559 hypothetical protein UniRef50_F7LNJ2,UniRef90_F7LNJ2

group_4023 100.8194946 1 99.81949458 0 4.613331736 4.613331736 hypothetical protein 559 hypothetical protein UniRef50_A0A0E2AWR3,UniRef90_A0A0E2AWR3

group_3860 100.8194946 1 99.81949458 0 4.613331736 4.613331736 acyltransferase 559 acyltransferase WP_130070936.1 UPI0010213968 UniRef100_A0A642HIU7,UniRef50_K5ZM26,UniRef90_I9VZ87

group_3821 100.8194946 1 99.81949458 0 4.613331736 4.613331736 SusC/RagA family protein;hypothetical protein 559 SusC/RagA family protein WP_130070953.1 UPI0010208923 UniRef100_A0A642HQF7,UniRef50_A0A353TCS5,UniRef90_E1WKN1

group_3449 100.8194946 1 99.81949458 0 4.613331736 4.613331736 hypothetical protein 559 hypothetical protein UniRef50_A0A0E2AKB0,UniRef90_A0A0E2AKB0

group_1937 100.6389892 1.714285714 98.92470346 0.538996501 4.611539749 4.072543248 DUF3316 domain-containing protein;hypothetical protein 559 DUF3316 domain-containing protein WP_005801667.1 UPI0001AFB719 UniRef100_A0A1C0WWH7,UniRef50_A0A0P0FK26,UniRef90_Q64ZB7

group_1616 100.8194946 1 99.81949458 0 4.613331736 4.613331736 RagB/SusD family nutrient uptake outer membrane protein 559 RagB/SusD family nutrient uptake outer membrane protein WP_005796584.1 UPI0002132520 UniRef100_F7LJH1,UniRef50_A0A108TA94,UniRef90_A0A015XEY0

group_1499 100.8194946 1 99.81949458 0 4.613331736 4.613331736 DNA-binding response regulator 559 DNA-binding response regulator WP_005783816.1 UPI000043E082 UniRef100_A0A4S3HEL8,UniRef50_G2EGV3,UniRef90_A0A2M9UPC3

kdsB 100.6389892 1.714285714 98.92470346 0.538996501 4.611539749 4.072543248 3-deoxy-manno-octulosonate cytidylyltransferase 559 3-deoxy-manno-octulosonate cytidylyltransferase WP_005787574.1 UPI000043E827 UniRef100_A0A0K6BU34,UniRef50_A0A0K6BU34,UniRef90_A0A0K6BU34

group_1340 100.8194946 1 99.81949458 0 4.613331736 4.613331736 hypothetical protein 559 hypothetical protein UPI0009BC99B2 UniRef100_A0A2M9V5M7,UniRef50_A0A149N182,UniRef90_A0A149N182

cobI 100.6389892 1.714285714 98.92470346 0.538996501 4.611539749 4.072543248 SAM-dependent methyltransferase;precorrin-2 C(20)-methyltransferase 559 precorrin-2 C(20)-methyltransferase WP_005787514.1 UPI000043E807 UniRef100_A0A0K6BV24,UniRef50_A0A415IDP8,UniRef90_A0A0K6BV24

group_1033 100.8194946 1 99.81949458 0 4.613331736 4.613331736 hypothetical protein 559 hypothetical protein UniRef50_A0A015YKB1,UniRef90_A0A015YKB1

group_19397 100.6389892 1 99.63898917 0 4.611539749 4.611539749 3-isopropylmalate dehydratase 558 3-isopropylmalate dehydratase WP_005789847.1 UPI0001AFBAE6 UniRef100_D1JS96,UniRef50_D1JS96,UniRef90_D1JS96

group_14697 100.6389892 1 99.63898917 0 4.611539749 4.611539749 hypothetical protein 558 hypothetical protein UniRef50_E1WVP2,UniRef90_E1WVP2

group_14687 100.6389892 1 99.63898917 0 4.611539749 4.611539749 hypothetical protein 558 hypothetical protein UPI0001AFB85B UniRef100_A0A7U8L0Z6,UniRef50_R5RM90,UniRef90_R5RM90

group_9824 100.6389892 1 99.63898917 0 4.611539749 4.611539749 Erythromycin esterase;hypothetical protein;ABC-2 family transporter protein 558 Erythromycin esterase UPI00123090F2 UniRef100_A0A5M5PQ81,UniRef50_I9AMJ8,UniRef90_I9AMJ8

group_7204 100.6389892 1 99.63898917 0 4.611539749 4.611539749 hypothetical protein;cell surface protein 558 cell surface protein WP_217723437.1 UPI001C379041 UniRef100_UPI001C379041,UniRef50_Q5LEP5,UniRef90_Q5LEP5

group_6552 100.6389892 1 99.63898917 0 4.611539749 4.611539749 Putative cobalt-precorrin-6A synthase [deacetylating];hypothetical protein;cobalt-precorrin-5B (C(1))-methyltransferase CbiD 558 cobalt-precorrin-5B (C(1))-methyltransferase CbiD WP_005793561.1 UPI0001BD943C UniRef100_D1JNV4,UniRef50_D1JNV4,UniRef90_D1JNV4

asnB 100.4584838 1.714285714 98.74419804 0.538996501 4.609744545 4.070748044 Asparagine synthetase B [glutamine-hydrolyzing];asparagine synthase B;Asparagine synthetase B;Asparagine synthetase B (Glutamine-hydrolyzing);hypothetical protein 558 asparagine synthase B UniRef50_Q54MB4,UniRef90_E1WQD6

nspC 100.4584838 1.714285714 98.74419804 0.538996501 4.609744545 4.070748044 Carboxynorspermidine decarboxylase;Carboxynorspermidine/carboxyspermidine decarboxylase;Type I restriction-modification system;hypothetical protein;carboxynorspermidine decarboxylase 558 carboxynorspermidine decarboxylase UniRef50_A0A6F9ZM47,UniRef90_A0A174KNP3

group_4487 100.6389892 1 99.63898917 0 4.611539749 4.611539749 putative aspartyl protease;hypothetical protein 558 putative aspartyl protease WP_130070892.1 UPI00101FEF56 UniRef100_A0A642HFW3,UniRef50_A0A174U162,UniRef90_Q5LC06

group_3670 100.6389892 1 99.63898917 0 4.611539749 4.611539749 ABC-type multidrug transport system ATPase and permease component subunits putative;hypothetical protein;ABC transporter ATP-binding protein 558 ABC transporter ATP-binding protein WP_130070836.1 UPI00101F6E6F UniRef100_A0A642F7X1,UniRef50_A0A174SKA1,UniRef90_D1JMN4

group_1383 100.6389892 1 99.63898917 0 4.611539749 4.611539749 DUF3795 domain-containing protein 558 DUF3795 domain-containing protein UniRef50_Q64V19,UniRef90_Q64V19

group_974 100.6389892 1 99.63898917 0 4.611539749 4.611539749 Glycosyltransferase family 1 protein;hypothetical protein 558 Glycosyltransferase family 1 protein WP_011202400.1 UPI000043E506 UniRef100_A0A642KX12,UniRef50_A0A7L5EB47,UniRef90_K1FUC9

group_937 100.6389892 1 99.63898917 0 4.611539749 4.611539749 hypothetical protein 558 hypothetical protein UniRef50_A0A015X2Q8,UniRef90_A0A015X2Q8

group_15985 100.4584838 1 99.45848375 0 4.609744545 4.609744545 sulfatase;Sulfatase domain-containing protein;DUF4976 domain-containing protein;hypothetical protein 557 sulfatase WP_032563540.1 UPI00044FFB4F UniRef100_A0A017N4I2,UniRef50_A0A2M9V2U9,UniRef90_A0A2M9V2U9

group_15212 100.4584838 1 99.45848375 0 4.609744545 4.609744545 hypothetical protein 557 hypothetical protein

group_15121 100.2779783 1.714285714 98.56369263 0.538996501 4.607946113 4.068949612 DUF3575 domain-containing protein 557 DUF3575 domain-containing protein UniRef50_A0A3E5CLU1,UniRef90_Q64MJ0

group_15048 100.4584838 1 99.45848375 0 4.609744545 4.609744545 Formylglycine-generating enzyme family protein;hypothetical protein;formylglycine-generating enzyme family protein 557 formylglycine-generating enzyme family protein WP_005793740.1 UPI000043E8DA UniRef100_A0A5C6JBA5,UniRef50_F3PQS3,UniRef90_A0A2M9V7E6

group_14721 100.4584838 1 99.45848375 0 4.609744545 4.609744545 DUF6078 domain-containing protein;hypothetical protein 557 hypothetical protein

group_14532 100.4584838 1 99.45848375 0 4.609744545 4.609744545 hypothetical protein 557 hypothetical protein WP_005786652.1 UPI0001AFBFF2 UniRef100_A0A149NA50,UniRef50_Q64VK1,UniRef90_Q64VK1

group_11788 100.0974729 2.428571429 97.6689015 0.887303195 4.60614444 3.718841245 hypothetical protein;beta-mannosidase 557 beta-mannosidase UniRef50_A0A098C1Z4,UniRef90_E1WLN8

group_10718 100.2779783 1.714285714 98.56369263 0.538996501 4.607946113 4.068949612 hypothetical protein 557 hypothetical protein

group_7852 100.4584838 1 99.45848375 0 4.609744545 4.609744545 FtsX domain-containing protein;hypothetical protein;ABC transporter permease 557 ABC transporter permease UPI001230EAEF UniRef100_A0A642KT05,UniRef50_Q5LDA2,UniRef90_A0A0E2APV0

group_7503 100.4584838 1 99.45848375 0 4.609744545 4.609744545 AraC-like ligand binding domain protein;AraC family transcriptional regulator;hypothetical protein 557 AraC family transcriptional regulator WP_032589729.1 UPI00044E8EBE UniRef100_UPI00044E8EBE,UniRef50_U6RFB5,UniRef90_R6YQ47

group_5163 100.2779783 1.714285714 98.56369263 0.538996501 4.607946113 4.068949612 hypothetical protein;SusC/RagA family TonB-linked outer membrane protein;TonB-linked outer membrane protein SusC/RagA family 557 SusC/RagA family TonB-linked outer membrane protein UniRef50_Q8A9E8,UniRef90_A0A0E2ALZ0

group_4698 100.4584838 1 99.45848375 0 4.609744545 4.609744545 hypothetical protein 557 hypothetical protein WP_009293017.1 UPI00021330E6 UniRef100_A0A016HU75,UniRef50_A0A016HU75,UniRef90_A0A016HU75

group_4618 100.4584838 1 99.45848375 0 4.609744545 4.609744545 integration host factor subunit alpha;hypothetical protein;Putative dNA-binding protein HU 557 integration host factor subunit alpha UniRef50_K1G5K2,UniRef90_K1G5K2

group_4214 100.4584838 1 99.45848375 0 4.609744545 4.609744545 TonB-dep-Rec domain-containing protein;hypothetical protein;TonB dependent receptor;SusC/RagA family TonB-linked outer membrane protein 557 SusC/RagA family TonB-linked outer membrane protein WP_130071210.1 UPI001021ED71 UniRef100_A0A642EXA3,UniRef50_Q8AAI1,UniRef90_Q5LED6

group_4204 100.4584838 1 99.45848375 0 4.609744545 4.609744545 MmcQ/YjbR family DNA-binding protein;YjbR 557 YjbR UniRef50_A0A174V6V3,UniRef90_A0A0K6BVA5

group_4164 100.2779783 1.714285714 98.56369263 0.538996501 4.607946113 4.068949612 hypothetical protein;SAM-dependent methyltransferase;Methyltransf-11 domain-containing protein 557 SAM-dependent methyltransferase UniRef50_A0A644YEM7,UniRef90_A0A380Z3M9

group_2476 100.4584838 1 99.45848375 0 4.609744545 4.609744545 hypothetical protein;Putative lipoprotein 557 hypothetical protein WP_008768924.1 UPI0001AFBA4F UniRef100_D1JNV5,UniRef50_Q64TA8,UniRef90_Q64TA8

group_1883 100.4584838 1 99.45848375 0 4.609744545 4.609744545 Putative transmembrane protein 557 Putative transmembrane protein UniRef50_E1WUB9,UniRef90_E1WUB9

cepA 100.2779783 1 99.27797834 0 4.607946113 4.607946113 Beta-lactamase2 domain-containing protein;CepA family extended-spectrum class A beta-lactamase;hypothetical protein;extended-spectrum class A beta-lactamase CepA-49 556 CepA family extended-spectrum class A beta-lactamase UniRef50_A0A0P0EXG0

group_15067 100.2779783 1 99.27797834 0 4.607946113 4.607946113 hypothetical protein 556 hypothetical protein UniRef50_R6ZRJ3,UniRef90_R6ZRJ3

group_7348 100.2779783 1 99.27797834 0 4.607946113 4.607946113 hypothetical protein 556 hypothetical protein UniRef50_E1WN76,UniRef90_E1WN76

group_4623 100.2779783 1 99.27797834 0 4.607946113 4.607946113 hypothetical protein;TonB-dependent receptor plug domain-containing protein;TonB-dependent receptor;Sulfate/thiosulfate import ATP-binding protein CysA 556 TonB-dependent receptor WP_130070833.1 UPI00101E9238 UniRef100_A0A642F835,UniRef50_A0A7Z8YCW7,UniRef90_A0A2M9VCG0

group_4250 100.2779783 1 99.27797834 0 4.607946113 4.607946113 TetR/AcrR family transcriptional regulator 556 TetR/AcrR family transcriptional regulator WP_182114751.1 UPI0015F40680 UniRef100_UPI0015F40680,UniRef50_K9EJX7,UniRef90_A0A0E2B1B1

group_4052 100.2779783 1 99.27797834 0 4.607946113 4.607946113 hypothetical protein 556 hypothetical protein UPI00033C5C02 UniRef100_R6ZH37,UniRef50_R6ZH37,UniRef90_R6ZH37

group_3669 100.2779783 1 99.27797834 0 4.607946113 4.607946113 Putative multidrug export ATP-binding/permease protein;hypothetical protein 556 Putative multidrug export ATP-binding/permease protein UniRef50_A0A174SKS5,UniRef90_A0A2M9VCG2

group_2640 100.2779783 1 99.27797834 0 4.607946113 4.607946113 hypothetical protein 556 hypothetical protein UniRef50_A0A413VCC7,UniRef90_A0A0E2APG0

group_2105 100.2779783 1 99.27797834 0 4.607946113 4.607946113 hypothetical protein;HmuY protein;HmuY family protein 556 HmuY protein UniRef50_G5GAZ1,UniRef90_F7LQM0

group_1159 100.2779783 1 99.27797834 0 4.607946113 4.607946113 class I SAM-dependent methyltransferase;hypothetical protein 556 class I SAM-dependent methyltransferase WP_032564820.1 UPI00044622C5 UniRef100_A0A642HUI7,UniRef50_E7RNU3,UniRef90_Q64UH2

group_1018 100.2779783 1 99.27797834 0 4.607946113 4.607946113 hypothetical protein;DUF4903 domain-containing protein 556 DUF4903 domain-containing protein WP_005803364.1 UPI00004E245B UniRef100_A0A7J4Y9P6,UniRef50_W0EQR6,UniRef90_A0A1C0WRU9

group_14850 98.83393502 6 92.83393502 1.791759469 4.593441018 2.801681548 5-amino-6-(5-phosphoribosylamino)uracil reductase;RibD-C domain-containing protein;hypothetical protein 555 RibD-C domain-containing protein UniRef50_I9VG83

group_14483 100.0974729 1 99.09747292 0 4.60614444 4.60614444 N-acetylgalactosamine-6-sulfatase;Sulfatase domain-containing protein;Arylsulfatase;hypothetical protein 555 N-acetylgalactosamine-6-sulfatase WP_130071193.1 UPI00101F738F UniRef100_A0A642F1G8,UniRef50_Q5LFU6,UniRef90_Q5LFU6

group_14482 100.0974729 1 99.09747292 0 4.60614444 4.60614444 thioredoxin;hypothetical protein;Thioredoxin 555 Thioredoxin UniRef50_A0A173V9J6

group_14469 100.0974729 1 99.09747292 0 4.60614444 4.60614444 hypothetical protein 555 hypothetical protein UPI000257A459 UniRef100_A0A0E2ASG3,UniRef50_A0A0E2ASG3,UniRef90_A0A0E2ASG3

group_14241 100.0974729 1 99.09747292 0 4.60614444 4.60614444 EamA family transporter;Membrane protein;hypothetical protein 555 EamA family transporter WP_005785992.1 UPI000043E4A0 UniRef100_A0A0K6BRS1,UniRef50_E1WRR6,UniRef90_E1WRR6

group_12042 99.73646209 2.428571429 97.30789067 0.887303195 4.602531328 3.715228133 hypothetical protein;phosphoesterase 555 phosphoesterase UniRef50_R5R863,UniRef90_Q5L9H2

group_7602 100.0974729 1 99.09747292 0 4.60614444 4.60614444 hypothetical protein;Peptidase family S41;TSPc domain-containing protein;C-terminal processing peptidase 555 Peptidase family S41 WP_130070832.1 UPI001020212B UniRef100_A0A642HUC1,UniRef50_F7LQL2,UniRef90_F7LQL2

group_7560 100.0974729 1 99.09747292 0 4.60614444 4.60614444 hypothetical protein 555 hypothetical protein UniRef50_A0A0K6BUG3,UniRef90_A0A0K6BUG3

group_5095 100.0974729 1 99.09747292 0 4.60614444 4.60614444 transporter;hypothetical protein 555 transporter UniRef50_E6SPZ2,UniRef90_F7LQL0

group_4857 100.0974729 1 99.09747292 0 4.60614444 4.60614444 hypothetical protein;6-bladed beta-propeller 555 6-bladed beta-propeller WP_005803746.1 UPI000043E990 UniRef100_A0A1C0X608,UniRef50_A0A0E2APH9,UniRef90_A0A0E2APH9

group_4745 100.0974729 1 99.09747292 0 4.60614444 4.60614444 phage tail protein;carboxypeptidase-like regulatory domain-containing protein;hypothetical protein 555 phage tail protein WP_032529446.1 UPI0004D771FD UniRef100_A0A5C6HIP1,UniRef50_R6Z9S9,UniRef90_R6Z9S9

group_3927 100.0974729 1 99.09747292 0 4.60614444 4.60614444 Putative exported protein 555 Putative exported protein WP_032561634.1 UPI00044EFF7C UniRef100_UPI00044EFF7C,UniRef50_Q64QT7,UniRef90_E1WUG9

group_2267 99.73646209 2.428571429 97.30789067 0.887303195 4.602531328 3.715228133 hypothetical protein;EamA family transporter;Putative membrane protein;Membrane protein 555 Membrane protein WP_005784893.1 UPI000043E2C5 UniRef100_A0A853PXA6,UniRef50_A0A415J3A0,UniRef90_A0A0E2AU00

group_15439 98.6534296 6 92.6534296 1.791759469 4.591612997 2.799853528 alpha/beta hydrolase;Peptidase-S15 domain-containing protein;Alpha/beta family hydrolase;hypothetical protein;Alpha/beta fold family hydrolase 554 alpha/beta hydrolase UniRef50_A0A2T5XVE0,UniRef90_Q64UJ8

group_14538 99.91696751 1 98.91696751 0 4.604339516 4.604339516 L-asparaginase 1;hypothetical protein 554 L-asparaginase 1 UniRef50_Q5LBZ1,UniRef90_A0A149N041

group_14488 99.91696751 1 98.91696751 0 4.604339516 4.604339516 hypothetical protein 554 hypothetical protein UPI0002132316 UniRef100_A0A1C0WKV1,UniRef50_A0A1C0WKV1,UniRef90_A0A1C0WKV1

group_14480 99.91696751 1 98.91696751 0 4.604339516 4.604339516 alcohol dehydrogenase;hypothetical protein;ADH-zinc-N domain-containing protein;ADH-N domain-containing protein 554 alcohol dehydrogenase UniRef50_A0A0F0CC44,UniRef90_K1FU33

group_14478 99.91696751 1 98.91696751 0 4.604339516 4.604339516 hypothetical protein 554 hypothetical protein WP_032529323.1 UPI00044E4B98 UniRef100_A0A829SIF9,UniRef50_A0A553EG35,UniRef90_A0A380YWD0

group_8052 99.91696751 1 98.91696751 0 4.604339516 4.604339516 hypothetical protein 554 hypothetical protein UniRef50_A0A016EJA2,UniRef90_A0A016EJA2

group_6222 99.91696751 1 98.91696751 0 4.604339516 4.604339516 transcriptional regulator 554 transcriptional regulator UniRef50_I9SAG6,UniRef90_I9VX07

group_5094 99.91696751 1 98.91696751 0 4.604339516 4.604339516 hypothetical protein;Cobalt-zinc-cadmium resistance protein;Cation efflux system protein CzcA;CusA/CzcA family heavy metal efflux RND transporter;Cobalt-zinc-cadmium resistance protein CzcA;AcrB/AcrD/AcrF family;AcrB/AcrD/AcrF family cation efflux system protein 554 CusA/CzcA family heavy metal efflux RND transporter WP_005803351.1 UPI000043E7A4 UniRef100_A0A396ES53,UniRef50_A0A174JSX0,UniRef90_K1FPU0

group_4984 99.91696751 1 98.91696751 0 4.604339516 4.604339516 hypothetical protein 554 hypothetical protein UniRef50_E6SRM8,UniRef90_D1JMM9

group_4875 99.91696751 1 98.91696751 0 4.604339516 4.604339516 efflux RND transporter permease subunit;AcrB/AcrD/AcrF family protein;RND transporter HAE1/HME family permease protein;multidrug transporter AcrB 554 multidrug transporter AcrB WP_005798910.1 UPI000043EC54 UniRef100_A0A0K6BX61,UniRef50_A0A0F5IUM4,UniRef90_Q5LAM7

group_3629 99.91696751 1 98.91696751 0 4.604339516 4.604339516 hypothetical protein;HlyD-D23 domain-containing protein;efflux transporter periplasmic adaptor subunit 554 efflux transporter periplasmic adaptor subunit WP_130070831.1 UPI00101FFE31 UniRef100_A0A642HU90,UniRef50_F5IYG2,UniRef90_A0A2M9VCH1

group_14440 99.73646209 1 98.73646209 0 4.602531328 4.602531328 DUF6769 domain-containing protein 553 DUF6769 domain-containing protein UPI0004486FD0 UniRef100_A0A016BY35,UniRef50_F7LQK6,UniRef90_F7LQK6

group_14242 99.73646209 1 98.73646209 0 4.602531328 4.602531328 Crp/Fnr family transcriptional regulator;Cyclic nucleotide-binding domain-containing protein;hypothetical protein 553 Crp/Fnr family transcriptional regulator UPI001230C501 UniRef100_A0A5M5PHE5,UniRef50_I9BLJ9,UniRef90_I9BLJ9

group_4624 99.73646209 1 98.73646209 0 4.602531328 4.602531328 hypothetical protein;Putative exported protein 553 Putative exported protein UniRef50_E1WMK0,UniRef90_E1WMK0

group_2760 99.73646209 1 98.73646209 0 4.602531328 4.602531328 hypothetical protein;leucine-rich repeat domain-containing protein 553 hypothetical protein WP_010992886.1 UPI00004E2459 UniRef100_A0A642L499,UniRef50_W0ERP7,UniRef90_F7LQL6

group_14666 99.37545126 1.714285714 97.66116555 0.538996501 4.598905114 4.059908613 hypothetical protein 552 hypothetical protein UPI0002579F0F UniRef100_K1FX01,UniRef50_K1FX01,UniRef90_K1FX01

group_6453 99.55595668 1 98.55595668 0 4.600719865 4.600719865 hypothetical protein 552 hypothetical protein

group_5613 99.37545126 1 98.37545126 0 4.598905114 4.598905114 hypothetical protein 551 hypothetical protein UniRef50_D1JPF5,UniRef90_D1JPF5

group_4944 99.37545126 1 98.37545126 0 4.598905114 4.598905114 hypothetical protein;LytTR family transcriptional regulator 551 LytTR family transcriptional regulator WP_130071439.1 UPI0010208238 UniRef100_A0A642EZ07,UniRef50_I8WI95,UniRef90_I9VYB2

group_4230 99.37545126 1 98.37545126 0 4.598905114 4.598905114 hypothetical protein 551 hypothetical protein UniRef50_I9ASJ9,UniRef90_I9ASJ9

group_4016 99.37545126 1 98.37545126 0 4.598905114 4.598905114 hypothetical protein;SOS response associated peptidase (SRAP);Transposase;transposase 551 Transposase WP_005787595.1 UPI00026932F6 UniRef100_UPI0009B5E67F,UniRef50_I9VFV8,UniRef90_I9VFV8

group_3522 99.37545126 1 98.37545126 0 4.598905114 4.598905114 hypothetical protein 551 hypothetical protein UniRef50_F7LWA0,UniRef90_F7LWA0

group_19076 98.6534296 3.142857143 95.51057246 1.145132304 4.591612997 3.446480693 hypothetical protein 550 hypothetical protein UniRef50_A0A0K6C0I0,UniRef90_A0A0K6C0I0

group_14252 99.01444043 1.714285714 97.30015472 0.538996501 4.595265702 4.056269202 DUF418 domain-containing protein;Putative transmembrane protein 550 DUF418 domain-containing protein WP_032581508.1 UPI0004515864 UniRef100_UPI0004515864,UniRef50_A0A1H6KKD1,UniRef90_I8WJ80

group_5287 99.19494585 1 98.19494585 0 4.597087064 4.597087064 hypothetical protein;TIGR01212 family radical SAM protein 550 TIGR01212 family radical SAM protein WP_014298320.1 UPI0001DDE363 UniRef100_A0A2M9V921,UniRef50_E6SUH9,UniRef90_Q64XP4

group_2672 99.19494585 1 98.19494585 0 4.597087064 4.597087064 alpha-galactosidase;hypothetical protein 550 alpha-galactosidase WP_005813509.1 UPI00028095EF UniRef100_A0A5M5XEW7,UniRef50_Q5LHX6,UniRef90_Q5LHX6

group_998 99.01444043 1.714285714 97.30015472 0.538996501 4.595265702 4.056269202 hypothetical protein 550 hypothetical protein UniRef50_D1JVS1,UniRef90_D1JVS1

group_461 99.19494585 1 98.19494585 0 4.597087064 4.597087064 hypothetical protein 550 hypothetical protein WP_005785989.1 UPI000257A473 UniRef100_E1WRR3,UniRef50_Q64WU2,UniRef90_E1WRR3

group_5168 99.01444043 1 98.01444043 0 4.595265702 4.595265702 Y-Y-Y domain-containing protein;hypothetical protein;histidine kinase 549 histidine kinase WP_032568052.1 UPI000448E519 UniRef100_UPI000448E519,UniRef50_A0A0E2AWA1,UniRef90_I9VXS2

group_15309 98.83393502 1 97.83393502 0 4.593441018 4.593441018 Aldehyde reductase;Aldo/keto reductase;Glyoxal reductase;hypothetical protein;aldo/keto reductase 548 Aldehyde reductase UniRef50_A0A380YYH8,UniRef90_A0A380YYH8

group_14250 98.83393502 1 97.83393502 0 4.593441018 4.593441018 aldo/keto reductase;hypothetical protein;Aldo-ket-red domain-containing protein 548 aldo/keto reductase WP_130070830.1 UPI0010228BA7 UniRef100_A0A642F8C5,UniRef50_A0A143WV70,UniRef90_A0A2M9VCI4

group_9690 98.83393502 1 97.83393502 0 4.593441018 4.593441018 hypothetical protein;Thioredoxin-like family protein;TlpA family protein disulfide reductase;Thioredoxin domain-containing protein;Thioredoxin-like-fold domain-containing protein 548 Thioredoxin domain-containing protein WP_121963259.1 UPI000EE52F34 UniRef100_A0A642HJL1,UniRef50_K1FUG7,UniRef90_K1FUG7

group_15152 98.6534296 1 97.6534296 0 4.591612997 4.591612997 Aldo-ket-red domain-containing protein;25-diketo-D-gluconic acid reductase;Uncharacterized oxidoreductase MSMEG_2407;Glyoxal reductase;25-didehydrogluconate reductase;Aldo/keto reductase family protein 547 2,5-diketo-D-gluconic acid reductase WP_032556674.1 UPI0004485492 UniRef100_A0A2M9UVQ2,UniRef50_I9FGG0,UniRef90_I9VGI1

group_14733 98.6534296 1 97.6534296 0 4.591612997 4.591612997 DUF4469 domain-containing protein;hypothetical protein 547 hypothetical protein

group_14641 97.93140794 4.571428571 93.35997937 1.519825754 4.584267315 3.064441561 hypothetical protein;6-bladed beta-propeller 547 hypothetical protein WP_011203752.1 UPI000043F135 UniRef100_Q64MG5,UniRef50_F7LJ34,UniRef90_F7LJ34

group_4768 98.6534296 1 97.6534296 0 4.591612997 4.591612997 hypothetical protein;TonB-dep-Rec domain-containing protein;SusC/RagA family TonB-linked outer membrane protein 547 SusC/RagA family TonB-linked outer membrane protein WP_005798139.1 UPI000043EE26 UniRef100_A0A0E2ALY5,UniRef50_U6RBW6,UniRef90_A0A0E2ALY5

group_15351 98.47292419 1 97.47292419 0 4.589781629 4.589781629 cystathionine beta-lyase;Aminotran-1-2 domain-containing protein;Aminotransferase class I and II;hypothetical protein;Cystathionine beta-lyase PatB 546 cystathionine beta-lyase WP_032570407.1 UPI000451AFB1 UniRef100_A0A016BW28,UniRef50_A0A0P0M586,UniRef90_Q5LDI3

group_14436 98.47292419 1 97.47292419 0 4.589781629 4.589781629 NADPH-flavin oxidoreductase;hypothetical protein;ABM domain-containing protein;Flavin-Reduct domain-containing protein 546 NADPH-flavin oxidoreductase UniRef50_I9SNT5,UniRef90_I9SNT5

group_15366 98.11191336 1.714285714 96.39762764 0.538996501 4.5861088 4.047112299 aldo/keto reductase;Aldo-ket-red domain-containing protein;hypothetical protein 545 aldo/keto reductase UniRef50_G1UT75,UniRef90_R6LDH5

group_14731 98.29241877 1 97.29241877 0 4.587946901 4.587946901 MATE family efflux transporter;Putative efflux protein MATE family;Putative cation efflux protein;hypothetical protein;MATE efflux family protein 545 MATE family efflux transporter UPI0012318088 UniRef100_A0A642KNN6,UniRef50_Q5LDI4,UniRef90_Q5LDI4

group_14606 98.29241877 1 97.29241877 0 4.587946901 4.587946901 hypothetical protein 545 hypothetical protein UPI0002132F4F UniRef100_A0A2M9V3Y3,UniRef50_A0A2M9V3Y3,UniRef90_A0A2M9V3Y3

group_14439 98.29241877 1 97.29241877 0 4.587946901 4.587946901 DUF3737 domain-containing protein;PF12541 family protein;hypothetical protein 545 DUF3737 domain-containing protein UniRef50_U2QGD5,UniRef90_E1WWM6

ung 98.29241877 1 97.29241877 0 4.587946901 4.587946901 Uracil-DNA glycosylase;uracil-DNA glycosylase 545 uracil-DNA glycosylase WP_005802535.1 UPI000043ED11 UniRef100_Q64QI5,UniRef50_A0RM89,UniRef90_Q5LA67

group_1481 98.29241877 1 97.29241877 0 4.587946901 4.587946901 hypothetical protein 545 hypothetical protein

group_14435 98.11191336 1 97.11191336 0 4.5861088 4.5861088 aldo/keto reductase;twin-arginine translocation signal domain-containing protein;Aldo-ket-red domain-containing protein;hypothetical protein;Putative dehydrogenase 544 aldo/keto reductase WP_005787280.1 UPI00025FD3CD UniRef100_A0A0E2AQY4,UniRef50_A0A174U4I9,UniRef90_R6LDH0

group_5093 98.11191336 1 97.11191336 0 4.5861088 4.5861088 SusC/RagA family protein;hypothetical protein;TonB dependent receptor;TonB-dep-Rec domain-containing protein 544 SusC/RagA family protein UniRef50_A0A5P3AX17,UniRef90_D1JJ71

group_4948 98.11191336 1 97.11191336 0 4.5861088 4.5861088 hypothetical protein 544 hypothetical protein

group_4251 98.11191336 1 97.11191336 0 4.5861088 4.5861088 Membrane protein;hypothetical protein 544 Membrane protein WP_195593882.1 UPI00189C9DC4 UniRef100_UPI00189C9DC4,UniRef50_W0EQR1,UniRef90_A0A0E2APX1

group_5918 97.93140794 1 96.93140794 0 4.584267315 4.584267315 PorT family protein;OMP-b-brl-2 domain-containing protein;hypothetical protein 543 PorT family protein UniRef50_E6SS99,UniRef90_E1WMJ6

group_7435 97.75090253 1 96.75090253 0 4.582422432 4.582422432 hypothetical protein 542 hypothetical protein UniRef50_D1JMM0,UniRef90_D1JMM0

group_7164 97.02888087 3.857142857 93.17173801 1.349926717 4.575008675 3.225081958 Sulfotransferase family;hypothetical protein 542 Sulfotransferase family WP_005811224.1 UPI00028092DD UniRef100_A0A3E5CHE3,UniRef50_Q5L8T9,UniRef90_Q5L8T9

group_4010 97.02888087 3.857142857 93.17173801 1.349926717 4.575008675 3.225081958 hypothetical protein 542 hypothetical protein UniRef50_A0A2K9H1J1,UniRef90_Q5L8U0

group_4592 97.75090253 1 96.75090253 0 4.582422432 4.582422432 hypothetical protein 541 hypothetical protein UniRef50_I9JU69,UniRef90_I9JU69

group_3727 97.57039711 1 96.57039711 0 4.580574139 4.580574139 hypothetical protein 541 hypothetical protein UniRef50_I9VFW8,UniRef90_I9VFW8

group_14892 97.20938628 1.714285714 95.49510057 0.538996501 4.576867273 4.037870773 hypothetical protein;DUF4221 domain-containing protein 540 hypothetical protein WP_032584408.1 UPI000447FD81 UniRef100_A0A015S228,UniRef50_Q5L9I7,UniRef90_Q5L9I7

group_8752 97.20938628 1 96.20938628 0 4.576867273 4.576867273 hypothetical protein 539 hypothetical protein

group_4917 97.20938628 1 96.20938628 0 4.576867273 4.576867273 hypothetical protein 539 hypothetical protein

group_1497 97.02888087 1 96.02888087 0 4.575008675 4.575008675 hypothetical protein 538 hypothetical protein

group_8844 96.84837545 1 95.84837545 0 4.573146616 4.573146616 DUF2589 domain-containing protein;hypothetical protein 537 DUF2589 domain-containing protein UniRef50_D1JTK3,UniRef90_D1JTK3

group_9702 96.66787004 1 95.66787004 0 4.571281083 4.571281083 hypothetical protein;Putative lipoprotein;DUF4906 domain-containing protein 536 DUF4906 domain-containing protein WP_008657217.1 UPI0001AFBC11 UniRef100_UPI0001AFBC11,UniRef50_Q64QH2,UniRef90_Q64QH2

group_6974 96.66787004 1 95.66787004 0 4.571281083 4.571281083 hypothetical protein;RNA-binding transcriptional accessory protein;RNA-binding protein;S1 RNA binding domain protein 536 RNA-binding transcriptional accessory protein UniRef50_H1DDY8,UniRef90_F7LP59

group_5585 96.30685921 1.714285714 94.59257349 0.538996501 4.567539544 4.028543043 DUF1566 domain-containing protein;hypothetical protein 535 DUF1566 domain-containing protein UniRef50_A0A0E2AZ27,UniRef90_A0A0E2AZ27

group_4091 96.48736462 1 95.48736462 0 4.569412063 4.569412063 AraC family transcriptional regulator;hypothetical protein 535 AraC family transcriptional regulator WP_009292719.1 UPI00021328F1 UniRef100_R6Z0N4,UniRef50_A0A7U8Q9V4,UniRef90_A0A7U8Q9V4

group_7478 96.30685921 1 95.30685921 0 4.567539544 4.567539544 DUF1566 domain-containing protein;Putative lipoprotein;hypothetical protein 534 Putative lipoprotein WP_032571133.1 UPI000446B085 UniRef100_A0A015Y9V6,UniRef50_R5RWG8,UniRef90_A0A015Y9V6

group_7271 96.12635379 1.714285714 94.41206808 0.538996501 4.565663511 4.026667011 hypothetical protein 534 hypothetical protein UPI00028096DA UniRef100_K1GMK9,UniRef50_K1GMK9,UniRef90_K1GMK9

group_7224 96.30685921 1 95.30685921 0 4.567539544 4.567539544 hypothetical protein;TonB-dependent receptor 534 TonB-dependent receptor UniRef50_A0A1M4T716,UniRef90_A0A016FLF1

group_4977 96.30685921 1 95.30685921 0 4.567539544 4.567539544 hypothetical protein;Putative lipoprotein 534 Putative lipoprotein UniRef50_E1WV12,UniRef90_E1WV12

group_4237 96.30685921 1 95.30685921 0 4.567539544 4.567539544 DUF3575 domain-containing protein;hypothetical protein 534 DUF3575 domain-containing protein WP_005790242.1 UPI000043ED1B UniRef100_A0A0K6BXP9,UniRef50_A0A0K6BXP9,UniRef90_A0A0K6BXP9

group_2529 96.30685921 1 95.30685921 0 4.567539544 4.567539544 hypothetical protein 534 hypothetical protein WP_014299281.1 UPI0001DDD664 UniRef100_E1WV14,UniRef50_E1WV14,UniRef90_E1WV14

group_18226 96.12635379 1 95.12635379 0 4.565663511 4.565663511 hypothetical protein 533 hypothetical protein WP_005788695.1 UPI000043EAF9 UniRef100_A0A0K6BWL2,UniRef50_A0A6I0SHJ6,UniRef90_A0A0K6BWL2

group_5034 95.94584838 1 94.94584838 0 4.563783953 4.563783953 OMP-b-brl-2 domain-containing protein 532 OMP-b-brl-2 domain-containing protein WP_032533455.1 UPI000446F065 UniRef100_A0A016EKN6,UniRef50_A0A7U8L8C5,UniRef90_A0A7U8L8C5

group_9275 95.76534296 1 94.76534296 0 4.561900855 4.561900855 DUF1566 domain-containing protein;fimbrillin family protein;Fimbrillin-like;Putative lipoprotein 531 DUF1566 domain-containing protein UniRef50_A0A6I0SGF0

group_7058 95.58483755 1 94.58483755 0 4.560014204 4.560014204 hypothetical protein 530 hypothetical protein

group_4598 95.76534296 1 94.76534296 0 4.561900855 4.561900855 ABC transporter ATP-binding protein;hypothetical protein 530 ABC transporter ATP-binding protein WP_005791987.1 UPI00004E28E3 UniRef100_A0A0K6BYY8,UniRef50_A0A0K6BYY8,UniRef90_A0A0K6BYY8

group_3972 95.58483755 1 94.58483755 0 4.560014204 4.560014204 Lipopolysaccharide kinase (Kdo/WaaP) family 529 Lipopolysaccharide kinase (Kdo/WaaP) family WP_005783846.1 UPI000043E091 UniRef100_F7LJR4,UniRef50_I9BPZ4,UniRef90_F7LJR4

group_7270 95.0433213 1.714285714 93.32903559 0.538996501 4.554332801 4.015336301 hypothetical protein 528 hypothetical protein UniRef50_A0A380Z215,UniRef90_A0A380Z215

group_5061 95.40433213 1 94.40433213 0 4.558123988 4.558123988 hypothetical protein;Erythromycin esterase;Erythromycin esterase family protein 528 Erythromycin esterase UniRef50_F7LWV5,UniRef90_F7LWV5

group_14239 95.22382671 1 94.22382671 0 4.556230191 4.556230191 hypothetical protein 527 hypothetical protein UniRef50_F7LMM1,UniRef90_F7LMM1

group_907 95.22382671 1 94.22382671 0 4.556230191 4.556230191 hypothetical protein 527 hypothetical protein

group_15310 94.68231047 1 93.68231047 0 4.550527187 4.550527187 hypothetical protein 525 hypothetical protein UniRef50_A0A0E2APM7,UniRef90_A0A0E2APM7

group_14593 94.50180505 1.714285714 92.78751934 0.538996501 4.548618935 4.009622435 AraC family transcriptional regulator;hypothetical protein;AraC family Bacterial regulatory helix-turn-helix protein 525 hypothetical protein

group_11459 94.32129964 2.428571429 91.89272821 0.887303195 4.546707035 3.65940384 DUF2961 domain-containing protein;hypothetical protein 525 DUF2961 domain-containing protein UniRef50_Q5L834,UniRef90_Q5L834

group_14604 94.68231047 1 93.68231047 0 4.550527187 4.550527187 hypothetical protein 524 hypothetical protein

group_14594 94.50180505 1 93.50180505 0 4.548618935 4.548618935 GTP cyclohydrolase;YciI family protein 524 GTP cyclohydrolase WP_005790301.1 UPI000043ED33 UniRef100_A0A149N3N2,UniRef50_D3IB55,UniRef90_A0A149N3N2

group_7608 94.50180505 1 93.50180505 0 4.548618935 4.548618935 hypothetical protein 524 hypothetical protein UniRef50_Q64X88,UniRef90_Q64X88

group_7151 94.68231047 1 93.68231047 0 4.550527187 4.550527187 hypothetical protein;glycosyl transferase 524 glycosyl transferase WP_005783851.1 UPI0002132C0D UniRef100_A0A1C0WN68,UniRef50_A0A7U8L3P9,UniRef90_A0A7U8L3P9

group_3983 94.68231047 1 93.68231047 0 4.550527187 4.550527187 glycosyl transferase;hypothetical protein;Putative glycosyltransferase 524 glycosyl transferase UniRef50_Q5LIR2,UniRef90_Q5LIR2

group_2540 94.50180505 1 93.50180505 0 4.548618935 4.548618935 hypothetical protein 524 hypothetical protein UniRef50_F7LLV6,UniRef90_F7LLV6

group_7254 94.14079422 1 93.14079422 0 4.544791473 4.544791473 ORF6N domain-containing protein;hypothetical protein 522 ORF6N domain-containing protein UniRef50_Q5L9M1,UniRef90_Q5L9M1

group_7838 93.96028881 1 92.96028881 0 4.542872234 4.542872234 hypothetical protein 521 hypothetical protein

group_12087 93.23826715 1 92.23826715 0 4.535158229 4.535158229 hypothetical protein 517 hypothetical protein

group_7519 93.41877256 1 92.41877256 0 4.537092316 4.537092316 hypothetical protein 517 hypothetical protein WP_053873857.1 UPI0006A669BE UniRef100_A0A5M5UDM5,UniRef50_D1JNU8,UniRef90_D1JNU8

group_4796 93.23826715 1 92.23826715 0 4.535158229 4.535158229 hypothetical protein;TonB-dependent receptor;TonB dependent receptor family protein 516 TonB-dependent receptor UniRef50_A0A069QSX8,UniRef90_Q5LAC2

group_4876 93.23826715 1 92.23826715 0 4.535158229 4.535158229 hypothetical protein;LruC domain-containing protein;DUF4842 domain-containing protein 515 LruC domain-containing protein WP_032533685.1 UPI000448613D UniRef100_A0A5C6JGY1,UniRef50_E1WU17,UniRef90_E1WU17

group_1431 92.87725632 1 91.87725632 0 4.531278797 4.531278797 Putative lipoprotein;hypothetical protein 514 Putative lipoprotein WP_041926249.1 UPI0005A542B0 UniRef100_A0A380YSF1,UniRef50_U2MBS5,UniRef90_D1JSA6

group_1071 92.87725632 1 91.87725632 0 4.531278797 4.531278797 hypothetical protein 514 hypothetical protein UniRef50_A0A380YP92,UniRef90_A0A380YP92

group_7501 92.33574007 1 91.33574007 0 4.525431283 4.525431283 hypothetical protein 512 hypothetical protein UniRef50_A0A5M5PHK8,UniRef90_A0A5M5PHK8

group_15176 91.25270758 2.428571429 88.82413615 0.887303195 4.513632664 3.626329469 hypothetical protein 508 hypothetical protein

group_4334 91.25270758 1 90.25270758 0 4.513632664 4.513632664 hypothetical protein 506 hypothetical protein UniRef50_A0A5M5PRL2,UniRef90_A0A5M5PRL2

group_13325 88.54512635 8.142857143 80.40226921 2.097141119 4.483512324 2.386371206 hypothetical protein;Membrane protein 500 hypothetical protein WP_120145246.1 UPI000E74EAFB UniRef100_A0A412YKH3,UniRef50_F0R8K6,UniRef90_I9SDZ7

group_2456 90.16967509 1 89.16967509 0 4.501693174 4.501693174 hypothetical protein;chloramphenicol acetyltransferase;Chloramphenicol acetyltransferase family protein 500 chloramphenicol acetyltransferase WP_008660317.1 UPI0001AFB991 UniRef100_A0A642F8W9,UniRef50_A0A380YZL9,UniRef90_A0A380YZL9

group_7492 89.80866426 1 88.80866426 0 4.497681455 4.497681455 hypothetical protein 498 hypothetical protein UniRef50_A0A2M9V4F2,UniRef90_A0A2M9V4F2

group_2315 89.98916968 1 88.98916968 0 4.499689326 4.499689326 hypothetical protein 498 hypothetical protein UniRef50_E6SWG4,UniRef90_Q5LDR9

group_4602 89.80866426 1 88.80866426 0 4.497681455 4.497681455 hypothetical protein;MFS domain-containing protein;Bcr/CflA family drug resistance efflux transporter;Bcr/CflA family efflux MFS transporter 497 Bcr/CflA family drug resistance efflux transporter UniRef50_A0A149NJA2,UniRef90_A0A149NJA2

group_1312 89.80866426 1 88.80866426 0 4.497681455 4.497681455 hypothetical protein;AraC family transcriptional regulator 497 AraC family transcriptional regulator WP_005798660.1 UPI00025FD5FB UniRef100_A0A415A5J5,UniRef50_A0A016BT27,UniRef90_A0A016BT27

group_7342 89.44765343 1 88.44765343 0 4.493653576 4.493653576 hypothetical protein;recombinase;Putative tyrosine recombinase XerD-like;Phage integrase family protein;Recombinase 495 recombinase UniRef50_A0A358PRK8,UniRef90_E1WVV8

group_7359 89.26714801 1 88.26714801 0 4.491633537 4.491633537 hypothetical protein 494 hypothetical protein UniRef50_A0A0E2AWH7,UniRef90_A0A0E2AWH7

group_17282 89.26714801 1 88.26714801 0 4.491633537 4.491633537 DUF3876 domain-containing protein 493 DUF3876 domain-containing protein WP_005788703.1 UPI000043EAFE UniRef100_D1JTJ9,UniRef50_Q5LBJ1,UniRef90_Q5LBJ1

group_4358 89.0866426 1 88.0866426 0 4.489609409 4.489609409 hypothetical protein;AraC family transcriptional regulator 493 AraC family transcriptional regulator WP_009292281.1 UPI0002133091 UniRef100_A0A642FN00,UniRef50_K9E4R5,UniRef90_D1JMN7

group_8225 89.0866426 1 88.0866426 0 4.489609409 4.489609409 LytTR family transcriptional regulator 492 LytTR family transcriptional regulator WP_050551125.1 UPI0006ACFEEF UniRef100_UPI0006ACFEEF,UniRef50_R5RY12,UniRef90_A0A149NFK8

group_7358 88.90613718 1 87.90613718 0 4.487581175 4.487581175 hypothetical protein 492 hypothetical protein

group_4533 88.72563177 1 87.72563177 0 4.485548819 4.485548819 GxxExxY protein 491 GxxExxY protein UniRef50_A0A2E2NYQ8,UniRef90_A0A0K6BPL3

group_7022 88.54512635 1 87.54512635 0 4.483512324 4.483512324 hypothetical protein;two-component sensor histidine kinase;ATPase/histidine kinase/DNA gyrase B/HSP90 domain protein;Histidine protein kinase divJ 490 two-component sensor histidine kinase WP_005794263.1 UPI0001BD979C UniRef100_D1JMN6,UniRef50_A0A2M9VCE9,UniRef90_A0A2M9VCE9

group_5609 88.00361011 1.714285714 86.28932439 0.538996501 4.477377838 3.938381337 hypothetical protein 489 hypothetical protein UPI000445DF9F UniRef100_A0A015WBI3,UniRef50_F7LPW8,UniRef90_F7LPW8

group_3631 88.00361011 1 87.00361011 0 4.477377838 4.477377838 hypothetical protein 488 hypothetical protein

group_1959 87.64259928 1 86.64259928 0 4.473267173 4.473267173 hypothetical protein 486 hypothetical protein UniRef50_I9B525,UniRef90_I9B525

group_5060 86.55956679 1 85.55956679 0 4.46083281 4.46083281 hypothetical protein 480 hypothetical protein

group_4497 85.65703971 1 84.65703971 0 4.450351413 4.450351413 hypothetical protein 473 hypothetical protein

group_10549 84.75451264 1 83.75451264 0 4.439758991 4.439758991 histidine kinase;hypothetical protein 470 histidine kinase UniRef50_A0A0E2AVC4,UniRef90_K1GT23

ynbB 84.75451264 1 83.75451264 0 4.439758991 4.439758991 phosphatidate cytidylyltransferase 470 phosphatidate cytidylyltransferase WP_005790388.1 UPI000043ED65 UniRef100_A0A0K6BY04,UniRef50_A0A0P0F817,UniRef90_A0A0P0F817

group_3841 84.75451264 1 83.75451264 0 4.439758991 4.439758991 hypothetical protein;1-acyl-sn-glycerol-3-phosphate acyltransferase 470 1-acyl-sn-glycerol-3-phosphate acyltransferase UniRef50_R6DHV6,UniRef90_A0A3E5ICC7

group_2053 84.75451264 1 83.75451264 0 4.439758991 4.439758991 CDP-diacylglycerol--glycerol-3-phosphate 3-phosphatidyltransferase;hypothetical protein;CDP-alcohol phosphatidyltransferase family protein 470 CDP-diacylglycerol--glycerol-3-phosphate 3-phosphatidyltransferase UniRef50_Q5L9Z3

group_4997 84.75451264 1 83.75451264 0 4.439758991 4.439758991 hypothetical protein;ATP-binding protein;AAA domain-containing protein 469 ATP-binding protein WP_042986886.1 UPI0004B4440C UniRef100_UPI0004B4440C,UniRef50_F7LQ66,UniRef90_F7LQ66

group_7691 82.76895307 8.857142857 73.91181021 2.181224236 4.416053028 2.234828792 hypothetical protein 467 hypothetical protein WP_121963447.1 UPI000ED4D5C2 UniRef100_UPI000ED4D5C2,UniRef50_A0A4S3I3P0,UniRef90_A0A4S3I3P0

group_1352 84.03249097 1 83.03249097 0 4.431203521 4.431203521 hypothetical protein 466 hypothetical protein UniRef50_A0A380YZX7,UniRef90_A0A380YZX7

group_8859 83.67148014 1.714285714 81.95719443 0.538996501 4.42689818 3.88790168 hypothetical protein 464 hypothetical protein

group_8151 82.94945848 3.857142857 79.09231563 1.349926717 4.418231488 3.068304771 winged helix-turn-helix domain-containing protein;hypothetical protein;OmpR/PhoB-type domain-containing protein 463 OmpR/PhoB-type domain-containing protein UPI000043E0A9 UniRef100_A0A3E5I6Y1,UniRef50_A0A3E5I6Y1,UniRef90_A0A3E5I6Y1

group_9531 82.76895307 3.857142857 78.91181021 1.349926717 4.416053028 3.066126311 NVEALA domain-containing protein;NVEALA family protein 462 hypothetical protein

group_19058 83.31046931 1.714285714 81.5961836 0.538996501 4.422574223 3.883577723 hypothetical protein 461 hypothetical protein UniRef50_A0A0E2AR84,UniRef90_A0A0E2AR84

group_7341 82.58844765 1 81.58844765 0 4.413869812 4.413869812 hypothetical protein 457 hypothetical protein WP_032564754.1 UPI000516E396 UniRef100_UPI000516E396,UniRef50_Q5LDS6,UniRef90_Q5LDS6

group_1320 82.58844765 1 81.58844765 0 4.413869812 4.413869812 hypothetical protein 456 hypothetical protein UniRef50_A0A0E2AS54,UniRef90_A0A0E2AS54

group_7672 82.04693141 1.714285714 80.33264569 0.538996501 4.407291418 3.868294917 hypothetical protein 455 hypothetical protein UniRef50_Q64QZ0,UniRef90_Q64QZ0

group_7789 81.32490975 3.142857143 78.1820526 1.145132304 4.398452363 3.253320058 hypothetical protein;6-bladed beta-propeller 453 6-bladed beta-propeller UniRef50_A0A395WN38,UniRef90_A0A395WN38

group_9360 80.96389892 3.142857143 77.82104177 1.145132304 4.394003363 3.248871059 hypothetical protein 451 hypothetical protein UniRef50_F7LJU0,UniRef90_F7LJU0

group_3961 80.96389892 1.714285714 79.2496132 0.538996501 4.394003363 3.855006862 hypothetical protein 449 hypothetical protein UniRef50_A0A0K6BNV5,UniRef90_A0A0K6BNV5

group_9108 80.7833935 1 79.7833935 0 4.391771418 4.391771418 hypothetical protein;DNA-binding protein;Virulence RhuM family protein;Putative dNA-binding protein 446 DNA-binding protein UniRef50_Q5LIB0,UniRef90_Q5LIB0

group_14814 80.06137184 1 79.06137184 0 4.382793489 4.382793489 hypothetical protein 443 hypothetical protein

group_9125 80.06137184 2.428571429 77.63280041 0.887303195 4.382793489 3.495490294 hypothetical protein 443 hypothetical protein WP_193684235.1 UPI0018799D92 UniRef100_UPI0018799D92,UniRef50_UPI0018799D92,UniRef90_UPI0018799D92

group_8121 78.97833935 1 77.97833935 0 4.369173629 4.369173629 4-oxalocrotonate tautomerase;Tautomerase 437 hypothetical protein

group_6334 79.15884477 1 78.15884477 0 4.371456527 4.371456527 hypothetical protein 434 hypothetical protein UniRef50_R6ZDK2,UniRef90_R6ZDK2

group_15186 78.07581227 1 77.07581227 0 4.357680307 4.357680307 Putative transmembrane protein;hypothetical protein 432 Putative transmembrane protein UniRef50_Q5L7N8,UniRef90_Q5L7N8

group_12094 75.36823105 9.571428571 65.79680248 2.25878247 4.322385847 2.063603377 Tyr recombinase domain-containing protein;integrase;Integrase;hypothetical protein;Tyrosine recombinase XerC;Arm-DNA-bind-5 domain-containing protein 427 integrase WP_005782064.1 UPI0001B49772 UniRef100_A0A7D4GEA3,UniRef50_K5ZEZ5,UniRef90_K5ZEZ5

group_15253 76.81227437 1.714285714 75.09798865 0.538996501 4.34136445 3.802367949 type II toxin-antitoxin system HigB family toxin 425 type II toxin-antitoxin system HigB family toxin UniRef50_A0A1M3H8L5,UniRef90_A0A6F9ZDY2

group_14775 76.81227437 1.714285714 75.09798865 0.538996501 4.34136445 3.802367949 transcriptional regulator 425 transcriptional regulator UPI0012312601 UniRef100_A0A5M5PF53,UniRef50_S2ECY1,UniRef90_R9IGC5

group_14617 76.45126354 1 75.45126354 0 4.33665346 4.33665346 hypothetical protein 423 hypothetical protein WP_005783715.1 UPI000043E04C UniRef100_A0A0K6BPI0,UniRef50_A0A0K6BPI0,UniRef90_A0A0K6BPI0

group_12294 75.36823105 6.714285714 68.65394533 1.904237453 4.322385847 2.418148395 ORF6N domain-containing protein;hypothetical protein 423 ORF6N domain-containing protein UniRef50_A0A1F3ET25

group_7673 76.63176895 1 75.63176895 0 4.339011729 4.339011729 hypothetical protein 423 hypothetical protein

group_1609 75.54873646 1 74.54873646 0 4.324777964 4.324777964 hypothetical protein 419 hypothetical protein

group_7139 75.54873646 1 74.54873646 0 4.324777964 4.324777964 hypothetical protein 417 hypothetical protein UPI0002809213 UniRef100_K1G7P5,UniRef50_I9BC61,UniRef90_I9BC61

group_4170 74.64620939 1 73.64620939 0 4.312759744 4.312759744 hypothetical protein;CMD domain-containing protein;Uncharacterized conserved protein YurZ alkylhydroperoxidase/carboxymuconolactone decarboxylase family;4-carboxymuconolactone decarboxylase;gamma-carboxymuconolactone decarboxylase;Cupin-2 domain-containing protein 412 gamma-carboxymuconolactone decarboxylase WP_011202637.1 UPI000043E6EC UniRef100_A0A415AIJ3,UniRef50_F7LQ15,UniRef90_F7LQ15

group_12102 72.29963899 8.142857143 64.15678185 2.097141119 4.280819136 2.183678017 hypothetical protein 410 hypothetical protein UPI00004E1F5C UniRef100_D1JQR1,UniRef50_D1JQR1,UniRef90_D1JQR1

group_13805 74.28519856 1.714285714 72.57091284 0.538996501 4.30791172 3.768915219 AraC family transcriptional regulator;Transcriptional regulator AraC family 409 AraC family transcriptional regulator WP_193683864.1 UPI0018795B83 UniRef100_UPI0018795B83,UniRef50_A0A0E2AQD9,UniRef90_UPI0011061E86

group_1323 73.92418773 1 72.92418773 0 4.303040078 4.303040078 AraC family transcriptional regulator 409 AraC family transcriptional regulator WP_005785567.1 UPI00004E2144 UniRef100_A0A0K6BRH2,UniRef50_A0A0K6BRH2,UniRef90_A0A0K6BRH2

ribB 73.74368231 1 72.74368231 0 4.300595328 4.300595328 Shikimate dehydrogenase (NADP(+));34-dihydroxy-2-butanone 4-phosphate synthase;aroE 408 shikimate dehydrogenase WP_130071340.1 UPI001021CE88 UniRef100_A0A642EYB1,UniRef50_Q5LGF4,UniRef90_Q5LGF4

group_7386 73.5631769 1 72.5631769 0 4.298144587 4.298144587 RNA polymerase subunit sigma-70 407 RNA polymerase subunit sigma-70 WP_005796768.1 UPI000043E0C4 UniRef100_A0A0E2AVY8,UniRef50_A0A449I098,UniRef90_A0A0E2AVY8

group_3867 73.5631769 1 72.5631769 0 4.298144587 4.298144587 hypothetical protein;anti-sigma factor 407 anti-sigma factor WP_005796770.1 UPI0001BD9456 UniRef100_D1JQR2,UniRef50_E1WM44,UniRef90_E1WM44

group_5147 73.38267148 1 72.38267148 0 4.295687824 4.295687824 STN domain-containing protein;SusC/RagA family TonB-linked outer membrane protein;TonB-dependent receptor;hypothetical protein 406 SusC/RagA family TonB-linked outer membrane protein UniRef50_F7LJV8,UniRef90_F7LJV8

group_1277 72.4801444 1 71.4801444 0 4.283312654 4.283312654 RagB/SusD family nutrient uptake outer membrane protein;hypothetical protein 402 RagB/SusD family nutrient uptake outer membrane protein WP_008769526.1 UPI000043E35D UniRef100_D1JPX2,UniRef50_D3I9B2,UniRef90_Q5LGY0

group_2753 72.29963899 2.428571429 69.87106756 0.887303195 4.280819136 3.393515941 hypothetical protein;Putative exported protein 399 Putative exported protein UniRef50_E1WM78,UniRef90_E1WM78

group_18971 71.93862816 1 70.93862816 0 4.275813369 4.275813369 hypothetical protein 396 hypothetical protein UPI00025FD336 UniRef100_A0A0E2B6X3,UniRef50_A0A0E2B6X3,UniRef90_A0A0E2B6X3

group_7527 71.57761733 1 70.57761733 0 4.270782418 4.270782418 bPH-5 domain-containing protein;hypothetical protein 396 bPH-5 domain-containing protein UniRef50_D1JWC0,UniRef90_D1JWC0

group_6084 71.03610108 1 70.03610108 0 4.263188214 4.263188214 hypothetical protein 391 hypothetical protein

group_4510 69.95306859 1 68.95306859 0 4.247824569 4.247824569 hypothetical protein 387 hypothetical protein UniRef50_A0A380YVC4,UniRef90_A0A380YVC4

group_4815 70.13357401 1 69.13357401 0 4.250401624 4.250401624 hypothetical protein 386 hypothetical protein UniRef50_Q5LBI0,UniRef90_Q5LBI0

group_1681 69.95306859 1 68.95306859 0 4.247824569 4.247824569 hypothetical protein 385 hypothetical protein WP_005815252.1 UPI00004E2616 UniRef100_D1JTI9,UniRef50_E1WS14,UniRef90_E1WS14

group_14696 69.41155235 1 68.41155235 0 4.240053314 4.240053314 hypothetical protein 384 hypothetical protein UPI00021321FD UniRef100_F7LKP0,UniRef50_F7LKP0,UniRef90_F7LKP0

group_12692 68.14801444 5.285714286 62.86230015 1.665007764 4.221682023 2.556674259 hypothetical protein 383 hypothetical protein WP_080714155.1 UPI0009B846CA UniRef100_A0A4P8LQX0,UniRef50_A0A0E2AP44,UniRef90_A0A4P8LQX0

group_14504 68.8700361 1 67.8700361 0 4.232221194 4.232221194 hypothetical protein 380 hypothetical protein UPI000268F7B4 UniRef100_I9VXI9,UniRef50_A0A0E2ASP4,UniRef90_A0A0E2ASP4

group_14286 68.32851986 1 67.32851986 0 4.224327247 4.224327247 AAA domain protein;adenylate kinase;hypothetical protein 377 adenylate kinase WP_009292012.1 UPI000043E648 UniRef100_F7LNW8,UniRef50_R9KGF5,UniRef90_F7LNW8

group_15203 68.14801444 1 67.14801444 0 4.221682023 4.221682023 hypothetical protein 376 hypothetical protein UniRef50_K1GRS5,UniRef90_K1GRS5

group_7255 68.50902527 1 67.50902527 0 4.226965492 4.226965492 hypothetical protein 376 hypothetical protein UniRef50_A0A380YYL6,UniRef90_A0A380YYL6

group_5056 66.88447653 6 60.88447653 1.791759469 4.2029669 2.411207431 hypothetical protein 376 hypothetical protein WP_008768416.1 UPI0001BD8DDB UniRef100_D1JLG7,UniRef50_F7LU36,UniRef90_Q64V65

group_4105 67.96750903 1 66.96750903 0 4.219029783 4.219029783 hypothetical protein 376 hypothetical protein WP_005794642.1 UPI00025FD777 UniRef100_A0A2M9UTP4,UniRef50_K9E6K6,UniRef90_Q5LDS3

group_3049 67.96750903 1 66.96750903 0 4.219029783 4.219029783 hypothetical protein 375 hypothetical protein UniRef50_Q64N92,UniRef90_Q64N92

group_6853 66.88447653 3.857142857 63.02733368 1.349926717 4.2029669 2.853040183 hypothetical protein 374 hypothetical protein WP_005797867.1 UPI000043ED89 UniRef100_A0A2M9VBG0,UniRef50_A0A380YWE2,UniRef90_A0A380YWE2

group_4433 66.88447653 3.857142857 63.02733368 1.349926717 4.2029669 2.853040183 ATPase AAA;hypothetical protein;DUF3696 domain-containing protein 374 DUF3696 domain-containing protein WP_011203374.1 UPI000043ED8A UniRef100_A0A2M9UUS9,UniRef50_R6ZF37,UniRef90_R6ZF37

group_4574 66.70397112 1 65.70397112 0 4.200264488 4.200264488 Arm-DNA-bind-5 domain-containing protein;hypothetical protein 368 Arm-DNA-bind-5 domain-containing protein UniRef50_I9BGF0,UniRef90_I9BGF0

group_5058 66.34296029 1 65.34296029 0 4.194837656 4.194837656 6-bladed beta-propeller;hypothetical protein;DUF4933 domain-containing protein 366 6-bladed beta-propeller WP_130070798.1 UPI00101ED8EB UniRef100_A0A642F802,UniRef50_Q5LDL7,UniRef90_Q5LDL7

group_4582 66.34296029 1 65.34296029 0 4.194837656 4.194837656 hypothetical protein;DUF4933 domain-containing protein 366 DUF4933 domain-containing protein WP_066402929.1 UPI000827B869 UniRef100_A0A853PX75,UniRef50_Q5LDL6,UniRef90_Q5LDL6

group_8180 66.5234657 1 65.5234657 0 4.197554753 4.197554753 hypothetical protein 365 hypothetical protein UniRef50_A0A0E2ANL1,UniRef90_A0A0E2ANL1

group_8202 65.62093863 1 64.62093863 0 4.183894831 4.183894831 hypothetical protein 362 hypothetical protein UniRef50_E1WNI5,UniRef90_K1G8X0

group_7268 65.44043321 1 64.44043321 0 4.181140312 4.181140312 hypothetical protein 360 hypothetical protein UniRef50_K1GCY8,UniRef90_A0A380YY16

group_7227 65.07942238 1 64.07942238 0 4.175608407 4.175608407 hypothetical protein 360 hypothetical protein UPI00025FD23A UniRef100_A0A015U5I7,UniRef50_A0A015U5I7,UniRef90_A0A015U5I7

group_4489 64.17689531 1 63.17689531 0 4.16164326 4.16164326 hypothetical protein 354 hypothetical protein

group_3975 63.99638989 1.714285714 62.28210418 0.538996501 4.158826674 3.619830173 sugar kinase;ROK family;hypothetical protein 354 sugar kinase WP_196036679.1 UPI0018A8E93C UniRef100_UPI0018A8E93C,UniRef50_A0A2M9V337,UniRef90_A0A2M9V337

group_3948 63.81588448 1 62.81588448 0 4.156002132 4.156002132 hypothetical protein;CPBP family intramembrane metalloprotease domain-containing protein;CPBP family intramembrane metalloprotease;CAAX protease self-immunity family protein;Abi (CAAX) family protease putative bacteriocin immunity protein 352 CPBP family intramembrane metalloprotease UPI001230DFF3 UniRef100_A0A5M5X3G1,UniRef50_I9HPB4,UniRef90_A0A0E2ASA1

group_13903 62.9133574 1.714285714 61.19907169 0.538996501 4.1417585 3.602762 hypothetical protein 349 hypothetical protein UniRef50_H1DEK0,UniRef90_A0A374MN73

group_9797 62.73285199 1 61.73285199 0 4.138885266 4.138885266 hypothetical protein 348 hypothetical protein UPI0005173283 UniRef100_A0A380YQ85,UniRef50_A0A380YQ85,UniRef90_A0A380YQ85

group_15693 60.74729242 7.428571429 53.31872099 2.00533357 4.106722512 2.101388942 hypothetical protein 345 hypothetical protein WP_195317116.1 UPI00189BE8FD UniRef100_UPI00189BE8FD,UniRef50_Q5LDS4,UniRef90_Q5LDS4

group_11760 62.19133574 1.714285714 60.47705003 0.538996501 4.130215693 3.591219193 DUF4369 domain-containing protein;hypothetical protein 342 DUF4369 domain-containing protein UniRef50_A0A0E2AVS1,UniRef90_A0A0E2AVS1

group_15525 61.83032491 1 60.83032491 0 4.124393938 4.124393938 Fimbrillin-like;hypothetical protein 338 Fimbrillin-like UniRef50_E1WP75,UniRef90_E1WP75

group_14281 61.83032491 1 60.83032491 0 4.124393938 4.124393938 Fimbrillin-like protein;Fimbrillin family protein;Fimbrillin-like;fimbrillin family protein;hypothetical protein 338 Fimbrillin-like WP_129659519.1 UPI00101B89C5 UniRef100_UPI00101B89C5,UniRef50_A0A0I9S5I3,UniRef90_E1WP78

group_15526 61.64981949 1 60.64981949 0 4.121470302 4.121470302 Fimbrillin-like;fimbrillin family protein;hypothetical protein;Putative exported lipoprotein 337 fimbrillin family protein WP_008660404.1 UPI000257A360 UniRef100_UPI000257A360,UniRef50_A0A4P8LH24,UniRef90_A0A0E2ANW1

group_7209 61.46931408 1 60.46931408 0 4.118538092 4.118538092 DUF4906 domain-containing protein;Putative exported transmembrane protein;hypothetical protein 336 DUF4906 domain-containing protein UniRef50_A0A015V7M8,UniRef90_K1GND3

group_15591 61.10830325 1 60.10830325 0 4.112647753 4.112647753 Fimbrillin-like;fimbrillin family protein;Fimbrillin-like protein;Fimbrillin family protein;hypothetical protein 334 Fimbrillin-like UniRef50_E1WP77,UniRef90_E1WP77

group_10082 60.74729242 1.714285714 59.0330067 0.538996501 4.106722512 3.567726011 hypothetical protein 334 hypothetical protein UniRef50_F7LUI7,UniRef90_F7LUI7

group_15122 60.566787 1 59.566787 0 4.103746674 4.103746674 hypothetical protein 333 hypothetical protein WP_008661599.1 UPI0001AFBC24 UniRef100_E1WRH9,UniRef50_Q64MU3,UniRef90_Q64MU3

group_8011 60.20577617 1 59.20577617 0 4.097768297 4.097768297 hypothetical protein 330 hypothetical protein UniRef50_F7LSF4,UniRef90_F7LSF4

group_7179 58.58122744 1 57.58122744 0 4.070414294 4.070414294 hypothetical protein 324 hypothetical protein UniRef50_A0A016GAX1,UniRef90_A0A833HT16

group_2637 58.58122744 1 57.58122744 0 4.070414294 4.070414294 hypothetical protein 324 hypothetical protein

group_2432 58.58122744 1 57.58122744 0 4.070414294 4.070414294 hypothetical protein 324 hypothetical protein

group_15465 58.94223827 1 57.94223827 0 4.076557952 4.076557952 Fimbrillin-like;hypothetical protein 322 Fimbrillin-like UniRef50_E1WP79,UniRef90_E1WP79

group_1192 58.40072202 1 57.40072202 0 4.067328253 4.067328253 hypothetical protein 322 hypothetical protein UniRef50_D1JVH2,UniRef90_D1JVH2

group_15472 58.76173285 1 57.76173285 0 4.073490841 4.073490841 Putative lipo domain protein;Fimbrillin-like;hypothetical protein;Putative lipoprotein 321 Fimbrillin-like UniRef50_E1WP80,UniRef90_E1WP80

group_10041 57.13718412 1 56.13718412 0 4.045455115 4.045455115 hypothetical protein;6-bladed beta-propeller 316 6-bladed beta-propeller WP_022347344.1 UPI0002D4EFA5 UniRef100_A0A642F694,UniRef50_A0A3E5GAJ1,UniRef90_I9BP15

group_8135 57.13718412 1 56.13718412 0 4.045455115 4.045455115 6-bladed beta-propeller;hypothetical protein 316 6-bladed beta-propeller WP_005801611.1 UPI000269226A UniRef100_A0A2M9V5S0,UniRef50_E1WKQ8,UniRef90_A0A2M9V5S0

group_4747 57.13718412 2.428571429 54.70861269 0.887303195 4.045455115 3.15815192 DUF6057 domain-containing protein;hypothetical protein 316 DUF6057 domain-containing protein UniRef50_Q5LGY1,UniRef90_Q5LGY1

group_4888 57.13718412 1 56.13718412 0 4.045455115 4.045455115 Beta-glucosidase BoGH3A;glycosyl hydrolase;Thermostable beta-glucosidase B;Putative beta-glucosidase Bgl7;hypothetical protein 315 glycosyl hydrolase WP_005798179.1 UPI0002132EE4 UniRef100_F7LSG6,UniRef50_A0A3S4R1D6,UniRef90_K1G220

group_5783 54.06859206 1 53.06859206 0 3.990253464 3.990253464 hypothetical protein 300 hypothetical protein UniRef50_E1WNY9,UniRef90_E1WNY9

group_2045 53.3465704 1 52.3465704 0 3.976809691 3.976809691 hypothetical protein 292 hypothetical protein UniRef50_Q64YG3,UniRef90_Q64YG3

group_7199 51.90252708 1 50.90252708 0 3.94936748 3.94936748 hypothetical protein;P-gingi-FimA domain-containing protein 285 P-gingi-FimA domain-containing protein WP_130070994.1 UPI00101F0570 UniRef100_A0A642HSB3,UniRef50_I9VJP1,UniRef90_I9VJP1

group_4882 51.72202166 1 50.72202166 0 3.945883642 3.945883642 hypothetical protein;Fimbrillin-A associated anchor proteins Mfa1 and Mfa2 284 Fimbrillin-A associated anchor proteins Mfa1 and Mfa2 WP_005790256.1 UPI0002693A3C UniRef100_A0A3E5IC14,UniRef50_E1WV19,UniRef90_A0A015ZNX1

lolD 51.36101083 1 50.36101083 0 3.93887934 3.93887934 ATP-binding cassette domain-containing protein;phosphonate ABC transporter ATP-binding protein 283 phosphonate ABC transporter ATP-binding protein WP_032567636.1 UPI0005174448 UniRef100_A0A2M9V3M3,UniRef50_A0A174RUE9,UniRef90_A0A0P0F8Y2

group_2464 51.18050542 1 50.18050542 0 3.935358706 3.935358706 hypothetical protein 281 hypothetical protein

group_9853 12.73285199 100.2857143 -87.5528623 4.608023255 2.544185424 -2.063837831 Fimbrillin-like 204 Fimbrillin-like UniRef50_A0A174I0L3,UniRef90_I9VHC5

group_7277 12.73285199 100.2857143 -87.5528623 4.608023255 2.544185424 -2.063837831 DUF2961 domain-containing protein;hypothetical protein 204 DUF2961 domain-containing protein WP_195460672.1 UPI001899DBAD UniRef100_UPI001899DBAD,UniRef50_Q5L834,UniRef90_Q5L834

group_11218 11.10830325 101 -89.89169675 4.615120517 2.407692869 -2.207427648 hypothetical protein;Aldehyde dehydrogenase family protein;aldehyde dehydrogenase;proline dehydrogenase;bifunctional proline dehydrogenase/L-glutamate gamma-semialdehyde dehydrogenase 196 proline dehydrogenase WP_005781362.1 UPI0001B49FF3 UniRef100_A0A7D4JLJ4,UniRef50_E4T356,UniRef90_A0A0I9S4W2

group_7355 12.19133574 96 -83.80866426 4.564348191 2.500725514 -2.063622677 hypothetical protein 196 hypothetical protein

group_8159 7.137184116 101 -93.86281588 4.615120517 1.965318317 -2.6498022 LruC domain-containing protein 176 LruC domain-containing protein UniRef50_E1WU17,UniRef90_A0A0I9S832

group_8063 7.137184116 100.2857143 -93.14853017 4.608023255 1.965318317 -2.642704938 hypothetical protein;T2SS-T3SS-pil-N domain-containing protein;pilus assembly protein N-terminal domain-containing protein 175 hypothetical protein

group_9909 6.776173285 99.57142857 -92.79525529 4.600875262 1.91341253 -2.687462731 alpha/beta hydrolase;hypothetical protein 171 alpha/beta hydrolase WP_032570928.1 UPI00044EDBB2 UniRef100_A0A015WBH5,UniRef50_A0A015WBH5,UniRef90_A0A015WBH5

group_4925 6.415162455 101 -94.58483755 4.615120517 1.858664322 -2.756456195 hypothetical protein;glycosyl transferase family 2 171 glycosyl transferase family 2 WP_080975842.1 UPI000453504C UniRef100_A0A5C6JJK7,UniRef50_A0A6N2X0S7,UniRef90_A0A396CC92

group_11203 6.59566787 98.85714286 -92.26147499 4.593675807 1.88641305 -2.707262756 hypothetical protein 169 hypothetical protein

group_5522 6.415162455 100.2857143 -93.87055183 4.608023255 1.858664322 -2.749358933 TonB-dependent receptor;hypothetical protein 169 TonB-dependent receptor WP_032574163.1 UPI000448A004 UniRef100_UPI000448A004,UniRef50_A0A1M4T716,UniRef90_A0A016FLF1

group_4926 6.415162455 98.14285714 -91.72769469 4.586424143 1.858664322 -2.727759821 alpha-12-fucosyltransferase 167 alpha-1,2-fucosyltransferase UPI0009B8D099 UniRef100_A0A5E8KA46,UniRef50_R5RQ30,UniRef90_R5RQ30

group_3991 6.415162455 98.14285714 -91.72769469 4.586424143 1.858664322 -2.727759821 glycosyltransferase family 2 protein;glycosyltransferase;hypothetical protein 167 glycosyltransferase family 2 protein WP_032530643.1 UPI0004479901 UniRef100_A0A2K9H3F5,UniRef50_A0A0I9S7P8,UniRef90_A0A0I9S7P8

group_4972 8.581227437 86.71428571 -78.13305828 4.462618642 2.149576961 -2.313041681 transcriptional regulator;hypothetical protein 164 transcriptional regulator WP_005781063.1 UPI0001B49555 UniRef100_A0A0I9S190,UniRef50_A0A0I9S190,UniRef90_A0A0I9S190

group_4343 8.581227437 86.71428571 -78.13305828 4.462618642 2.149576961 -2.313041681 Type 1 glutamine amidotransferase-like domain-containing protein;hypothetical protein;peptidase S51 164 peptidase S51 UniRef50_A0A395UT15,UniRef90_A0A0I9RQW9

group_7454 4.971119134 98.14285714 -93.17173801 4.586424143 1.603644993 -2.982779151 Sulfotransferase family;hypothetical protein 158 Sulfotransferase family WP_122348881.1 UPI000EFE7343 UniRef100_UPI000EFE7343,UniRef50_Q5L8T9,UniRef90_Q5L8T9

group_4789 4.971119134 98.14285714 -93.17173801 4.586424143 1.603644993 -2.982779151 hypothetical protein 158 hypothetical protein UniRef50_A0A2K9H1J1,UniRef90_A0A2K9H1J1

glnA3 4.068592058 100.2857143 -96.21712223 4.608023255 1.403297008 -3.204726247 glutamine synthetase type III;Glutamine synthetase;hypothetical protein;glutamine synthetase III 157 glutamine synthetase type III WP_032570764.1 UPI0004482307 UniRef100_A0A5M5W6M8,UniRef50_P15623,UniRef90_K9E5M9

gltD 3.888086643 100.2857143 -96.39762764 4.608023255 1.357917171 -3.250106084 Glutamate synthase NADH/NADPH small subunit domain protein;Glutamate synthase [NADPH] small chain;glutamate synthase;NAD(P)-binding protein;hypothetical protein 156 Glutamate synthase, NADH/NADPH, small subunit domain protein WP_115473755.1 UPI000E1DDA26 UniRef100_A0A642KQE9,UniRef50_A0A3C0V9B8,UniRef90_A0A015VCL6

gltB 3.888086643 99.57142857 -95.68334193 4.600875262 1.357917171 -3.242958091 Glutamate synthase large subunit;hypothetical protein;glutamate synthase large subunit 155 Glutamate synthase large subunit WP_122287987.1 UPI000F000017 UniRef100_A0A5M5PLT0,UniRef50_A0A7W8GED8,UniRef90_A0A5M5PLT0

group_9198 4.610108303 97.42857143 -92.81846313 4.579119509 1.52825135 -3.050868159 hypothetical protein;Fimbrillin-A associated anchor proteins Mfa1 and Mfa2;FimB/Mfa2 family fimbrial subunit 155 FimB/Mfa2 family fimbrial subunit WP_220654688.1 UPI001C7E145C UniRef100_UPI001C7E145C,UniRef50_K9EMC8,UniRef90_K1FT10

group_11178 3.707581227 100.2857143 -96.57813306 4.608023255 1.310379704 -3.297643551 NVEALA protein;NVEALA domain-containing protein;hypothetical protein 154 NVEALA protein WP_005801299.1 UPI000268EF0B UniRef100_I9BNM9,UniRef50_I9BNM9,UniRef90_I9BNM9

group_5475 8.581227437 77.42857143 -68.84734399 4.349355852 2.149576961 -2.199778891 hypothetical protein;arsenite S-adenosylmethyltransferase 150 arsenite S-adenosylmethyltransferase UniRef50_A0A847L7Z2,UniRef90_A0A7D4G6Y6

group_4932 2.805054152 101 -98.19494585 4.615120517 1.031422844 -3.583697673 TIGR01212 family radical SAM protein 150 TIGR01212 family radical SAM protein WP_005801247.1 UPI0002692264 UniRef100_I9BMM4,UniRef50_E6SUH9,UniRef90_Q64XP4

group_2266 8.400722022 78.14285714 -69.74213512 4.358538653 2.128317657 -2.230220996 Transcriptional regulator;transcriptional regulator 150 hypothetical protein

group_2092 2.805054152 101 -98.19494585 4.615120517 1.031422844 -3.583697673 LytTR family transcriptional regulator 150 LytTR family transcriptional regulator UniRef50_I8WI95,UniRef90_I9VYB2

group_12186 2.624548736 101 -98.37545126 4.615120517 0.964908971 -3.650211545 His Kinase A domain protein;Sensor histidine kinase;hypothetical protein;Histidine kinase 149 Histidine kinase UniRef50_A0A853PVD8,UniRef90_A0A4P8MQE3

group_5656 3.346570397 98.14285714 -94.79628675 4.586424143 1.207936059 -3.378488084 hypothetical protein 149 hypothetical protein UPI0009B83848 UniRef100_A0A396BT82,UniRef50_A0A396BT82,UniRef90_R5RC41

group_9339 2.805054152 99.57142857 -96.76637442 4.600875262 1.031422844 -3.569452418 hypothetical protein;6-bladed beta-propeller 148 6-bladed beta-propeller UniRef50_A0A0E2APH9,UniRef90_A0A0E2APH9

group_9126 2.444043321 101 -98.55595668 4.615120517 0.893653767 -3.72146675 hypothetical protein;Outer membrane efflux protein;TolC family protein 148 Outer membrane efflux protein WP_005780160.1 UPI0001B49533 UniRef100_A0A081TWE0,UniRef50_R5X1U2,UniRef90_A0A0K6BWY4

group_18236 6.054151625 86 -79.94584838 4.454347296 1.800744256 -2.653603041 hypothetical protein 147 hypothetical protein WP_005779961.1 UPI0001B48F48 UniRef100_A0A0I9RW45,UniRef50_A0A6I0SHJ6,UniRef90_A0A0K6BWL2

group_4125 2.624548736 99.57142857 -96.94687983 4.600875262 0.964908971 -3.63596629 EamA family transporter;hypothetical protein 147 EamA family transporter UniRef50_A0A415J3A0,UniRef90_A0A0E2AU00

group_132 2.263537906 101 -98.73646209 4.615120517 0.816929034 -3.798191482 multidrug transporter AcrB 147 multidrug transporter AcrB WP_032535708.1 UPI0005163164 UniRef100_A0A412Y5K8,UniRef50_A0A0F5IUM4,UniRef90_A0A2K9HB61

group_11175 6.9566787 81 -74.0433213 4.394449155 1.939702162 -2.454746992 endonuclease VIII 146 endonuclease VIII WP_032561510.1 UPI000452F53F UniRef100_UPI000452F53F,UniRef50_A0A0P0FR28,UniRef90_A0A0I9S5K0

group_2647 4.068592058 93.14285714 -89.07426509 4.534134413 1.403297008 -3.130837405 DUF4870 domain-containing protein 146 DUF4870 domain-containing protein WP_005807718.1 UPI00028085B9 UniRef100_A0A081U052,UniRef50_Q64R06,UniRef90_E1WU32

group_15461 6.9566787 79.57142857 -72.61474987 4.376655091 1.939702162 -2.436952929 hypothetical protein 144 hypothetical protein

group_8730 1.541516245 101 -99.45848375 4.615120517 0.432766507 -4.18235401 hypothetical protein;Membrane protein 143 Membrane protein WP_005778402.1 UPI0001B4A1AE UniRef100_A0A0I9SAH0,UniRef50_E6SRR2,UniRef90_Q64ST9

group_7135 1.541516245 101 -99.45848375 4.615120517 0.432766507 -4.18235401 Putative transmembrane protein 143 Putative transmembrane protein WP_235331132.1 UPI001F3BF434 UniRef100_UPI001F3BF434,UniRef50_E1WUB9,UniRef90_A0A0I9SCS4

group_4632 1.902527076 99.57142857 -97.6689015 4.600875262 0.643183042 -3.957692219 hypothetical protein 143 hypothetical protein WP_032594590.1 UPI00044B1A94 UniRef100_A0A015YID3,UniRef50_Q64QT7,UniRef90_A0A081U0C2

group_15013 1.722021661 99.57142857 -97.84940691 4.600875262 0.543498985 -4.057376277 6-bladed beta-propeller 142 6-bladed beta-propeller WP_005781833.1 UPI0001B48F86 UniRef100_A0A7D4KYS1,UniRef50_F7LVH9,UniRef90_A0A0I9S9N2

group_11986 1.722021661 99.57142857 -97.84940691 4.600875262 0.543498985 -4.057376277 hypothetical protein;UDP-GlcNAc--UDP-phosphate GlcNAc-1-phosphate transferase 142 UDP-GlcNAc--UDP-phosphate GlcNAc-1-phosphate transferase WP_005778119.1 UPI0001B49933 UniRef100_A0A4V1ET56,UniRef50_A0A174JAZ5,UniRef90_A0A0I9SA69

group_9000 1.36101083 101 -99.63898917 4.615120517 0.308227681 -4.306892836 AGE family epimerase/isomerase;N-acylglucosamine 2-epimerase 142 N-acylglucosamine 2-epimerase WP_005776495.1 UPI0001B49F5C UniRef100_A0A081UCD6,UniRef50_A0A0P0LCG9,UniRef90_A0A2M9URA4

cfiA~~~cfiA21~~~cfiA14 1.541516245 100.2857143 -98.74419804 4.608023255 0.432766507 -4.175256748 CfiA family subclass B1 metallo-beta-lactamase;subclass B1 metallo-beta-lactamase CfiA21;subclass B1 metallo-beta-lactamase CfiA/CcrA;subclass B1 metallo-beta-lactamase CfiA14 142 CfiA family subclass B1 metallo-beta-lactamase UniRef50_P25910,UniRef90_P25910

group_3257 5.151624549 86 -80.84837545 4.454347296 1.639312111 -2.815035185 hypothetical protein;DUF2589 domain-containing protein 142 DUF2589 domain-containing protein WP_005779963.1 UPI0001EC40C3 UniRef100_A0A081UHQ4,UniRef50_D1JTK3,UniRef90_D1JTK3

group_2057 1.541516245 100.2857143 -98.74419804 4.608023255 0.432766507 -4.175256748 DNA polymerase III subunit delta 142 DNA polymerase III subunit delta WP_005783087.1 UPI0001B4939B UniRef100_A0A0I9SBC4,UniRef50_A0A174H5G4,UniRef90_A0A2M9V3D5

group_15192 1.180505415 101 -99.81949458 4.615120517 0.165942665 -4.449177852 alpha-L-fucosidase;hypothetical protein;glycoside hydrolase family 95 protein 141 alpha-L-fucosidase WP_032535561.1 UPI000516F6F1 UniRef100_A0A396C1C6,UniRef50_A0A0I9S6K0,UniRef90_A0A0I9S6K0

group_13026 1.180505415 101 -99.81949458 4.615120517 0.165942665 -4.449177852 ABC transporter domain-containing protein;hypothetical protein;ABC transporter family protein;phosphonate ABC transporter ATP-binding protein 141 phosphonate ABC transporter ATP-binding protein WP_005777044.1 UPI0001B49B1E UniRef100_A0A081U5X0,UniRef50_A0A011P6Z7,UniRef90_I9KFF8

group_12270 6.23465704 80.28571429 -74.05105725 4.385591701 1.830123572 -2.555468129 redoxin domain-containing protein;AhpC-TSA domain-containing protein 141 redoxin domain-containing protein WP_121963982.1 UPI000EEEF3F2 UniRef100_UPI000EEEF3F2,UniRef50_A0A3E5CGV7,UniRef90_UPI000EEEF3F2

group_12033 1.180505415 101 -99.81949458 4.615120517 0.165942665 -4.449177852 Mannan endo-14-beta-mannosidase;hypothetical protein;beta-mannosidase 141 beta-mannosidase UniRef50_A0A098C1Z4,UniRef90_A0A0I9SAI6

group_11345 1.36101083 100.2857143 -98.92470346 4.608023255 0.308227681 -4.299795574 hypothetical protein;TonB-dependent receptor;SusC/RagA family TonB-linked outer membrane protein 141 SusC/RagA family TonB-linked outer membrane protein WP_032541827.1 UPI0001B49FE9 UniRef100_A0A7D4FQV0,UniRef50_Q8A9E8,UniRef90_A0A0E2ALZ0

group_7421 1.180505415 101 -99.81949458 4.615120517 0.165942665 -4.449177852 beta-N-acetylglucosaminidase;hypothetical protein;serine hydrolase 141 beta-N-acetylglucosaminidase UniRef50_F3ZSA4,UniRef90_A0A7U8QCM5

group_6833 1.180505415 101 -99.81949458 4.615120517 0.165942665 -4.449177852 hypothetical protein;Carboxynorspermidine/carboxyspermidine decarboxylase;carboxynorspermidine decarboxylase 141 carboxynorspermidine decarboxylase UniRef50_A0A6F9ZM47,UniRef90_A0A174KNP3

cbiD 1.180505415 101 -99.81949458 4.615120517 0.165942665 -4.449177852 cobalt-precorrin-5B (C(1))-methyltransferase CbiD;Precorrin-6x reductase CbiJ/CobK/protein CbiD multi-domain protein;Putative cobalt-precorrin-6A synthase [deacetylating];hypothetical protein 141 cobalt-precorrin-5B (C(1))-methyltransferase CbiD UniRef50_E5CEQ5,UniRef90_A0A0I9SAF6

group_4252 1.180505415 101 -99.81949458 4.615120517 0.165942665 -4.449177852 nitroreductase;hypothetical protein 141 nitroreductase UniRef50_A6LCN8,UniRef90_A0A0I9S8B4

group_3981 1.36101083 100.2857143 -98.92470346 4.608023255 0.308227681 -4.299795574 hypothetical protein;DUF3316 domain-containing protein 141 DUF3316 domain-containing protein UniRef50_A0A0P0FK26,UniRef90_Q64ZB7

group_2429 1.541516245 99.57142857 -98.02991233 4.600875262 0.432766507 -4.168108755 hypothetical protein 141 hypothetical protein UniRef50_R5RXU1,UniRef90_R5RXU1

group_1321 1.180505415 101 -99.81949458 4.615120517 0.165942665 -4.449177852 Uncharacterized conserved protein 141 Uncharacterized conserved protein WP_005777921.1 UPI0001B49301 UniRef100_A0A7D4JSE6,UniRef50_A0A379MNP4,UniRef90_A0A0I9SAH7

group_908 1.180505415 101 -99.81949458 4.615120517 0.165942665 -4.449177852 hypothetical protein 141 hypothetical protein UniRef50_A0A0I9UPW6,UniRef90_A0A0I9UPW6

group_18676 1 101 -100 4.615120517 0 -4.615120517 hypothetical protein 140 hypothetical protein WP_005778894.1 UPI0001B48F2C UniRef100_A0A081TYV7,UniRef50_A0A0N7IAW9,UniRef90_A0A081TYV7

group_17462 1 101 -100 4.615120517 0 -4.615120517 hypothetical protein 140 hypothetical protein WP_171810368.1 UPI001493F8F8 UniRef100_A0A7D4JMP8,UniRef50_A0A069CXQ2,UniRef90_A0A414ZYR5

group_16681 1 101 -100 4.615120517 0 -4.615120517 pyridoxamine 5'-phosphate oxidase 140 pyridoxamine 5'-phosphate oxidase WP_005775889.1 UPI0001B49F36 UniRef100_A0A081UL16,UniRef50_I9RVF6,UniRef90_A0A081UL16

group_16256 1 101 -100 4.615120517 0 -4.615120517 SNARE associated Golgi protein 140 SNARE associated Golgi protein WP_005779653.1 UPI0001B4A870 UniRef100_A0A081U860,UniRef50_A0A0P0FWC7,UniRef90_A0A0K6BND2

group_12751 1 101 -100 4.615120517 0 -4.615120517 TetR family transcriptional regulator 140 TetR family transcriptional regulator WP_005782733.1 UPI0001B4AB20 UniRef100_A0A7D4JWI6,UniRef50_R5I1D8,UniRef90_A0A081UBG7

group_11331 1 101 -100 4.615120517 0 -4.615120517 Carbohydrate-binding family 9 140 Carbohydrate-binding family 9 WP_005779568.1 UPI0001B4A623 UniRef100_A0A081U812,UniRef50_E5WW92,UniRef90_D1JQI1

group_8874 1 101 -100 4.615120517 0 -4.615120517 two-component system response regulator 140 two-component system response regulator WP_217737488.1 UPI001C3869CE UniRef100_UPI001C3869CE,UniRef50_A0A0K6BVB7,UniRef90_A0A081UJY7

group_8848 1 101 -100 4.615120517 0 -4.615120517 DUF5009 domain-containing protein;hypothetical protein 140 DUF5009 domain-containing protein WP_193683159.1 UPI001879C734 UniRef100_UPI001879C734,UniRef50_B0NSH7,UniRef90_A0A0I9SD66

group_8591 1 101 -100 4.615120517 0 -4.615120517 hypothetical protein 140 hypothetical protein UniRef50_R5RWW4,UniRef90_R5RWW4

group_8523 1 101 -100 4.615120517 0 -4.615120517 DNA-binding protein;HU-HIG domain-containing protein;hypothetical protein 140 hypothetical protein

group_7747 1 101 -100 4.615120517 0 -4.615120517 acyl-ACP--UDP-N-acetylglucosamine O-acyltransferase 140 acyl-ACP--UDP-N-acetylglucosamine O-acyltransferase WP_005779566.1 UPI0001B4A622 UniRef100_A0A4P8LA76,UniRef50_A0A0P0F0Z8,UniRef90_A0A0K6BNF0

group_7570 1 101 -100 4.615120517 0 -4.615120517 hypothetical protein;6-bladed beta-propeller 140 6-bladed beta-propeller WP_005780762.1 UPI0001B4AAEB UniRef100_A0A0I9TZ74,UniRef50_A0A0I9S7D4,UniRef90_A0A0I9S7D4

group_7462 1.180505415 100.2857143 -99.10520887 4.608023255 0.165942665 -4.44208059 hypothetical protein;formylglycine-generating enzyme family protein 140 formylglycine-generating enzyme family protein WP_005822420.1 UPI0001B4AD01 UniRef100_A0A2K9H2K4,UniRef50_F3PQS3,UniRef90_R5RHF3

group_7460 1 101 -100 4.615120517 0 -4.615120517 Aminotransferase;hypothetical protein;Aminotransferase class I and II;Aminotran-1-2 domain-containing protein;cystathionine beta-lyase 140 cystathionine beta-lyase WP_005777596.1 UPI0001B4AA70 UniRef100_A0A7D4JPC0,UniRef50_A0A0P0M586,UniRef90_A0A0I9UQ41

group_7324 1 101 -100 4.615120517 0 -4.615120517 hypothetical protein 140 hypothetical protein UniRef50_A0A2K9H0C3,UniRef90_A0A2K9H0C3

group_7101 1 101 -100 4.615120517 0 -4.615120517 RNA polymerase sigma-70 factor 140 RNA polymerase sigma-70 factor WP_220391312.1 UPI001C704858 UniRef100_UPI001C704858,UniRef50_A0A0E2AM90,UniRef90_A0A0E2AM90

group_6485 1 101 -100 4.615120517 0 -4.615120517 Ribosomal RNA large subunit methyltransferase J 140 Ribosomal RNA large subunit methyltransferase J WP_220575424.1 UPI001C731B7D UniRef100_UPI001C731B7D,UniRef50_A0A0N7I9J7,UniRef90_A0A0I9S8J4

group_6476 1.180505415 100.2857143 -99.10520887 4.608023255 0.165942665 -4.44208059 MBL fold metallo-hydrolase 140 MBL fold metallo-hydrolase WP_193684201.1 UPI001879D5DF UniRef100_UPI001879D5DF,UniRef50_A0A120A243,UniRef90_D1JTH4

group_6049 1 101 -100 4.615120517 0 -4.615120517 hypothetical protein;Asparagine synthetase B;asparagine synthase B;Asparagine synthetase B (Glutamine-hydrolyzing) 140 asparagine synthase B WP_122143174.1 UPI000EFD56DC UniRef100_A0A412Y0A3,UniRef50_Q54MB4,UniRef90_E1WQD6

group_5610 1 101 -100 4.615120517 0 -4.615120517 integration host factor subunit alpha 140 integration host factor subunit alpha WP_032544110.1 UPI00051648ED UniRef100_A0A396C327,UniRef50_K1G5K2,UniRef90_R5S8A1

group_5265 1 101 -100 4.615120517 0 -4.615120517 glutamate formimidoyltransferase 140 glutamate formimidoyltransferase WP_005782240.1 UPI0001B49BD4 UniRef100_A0A396BYX0,UniRef50_A6TVD7,UniRef90_A7LT54

group_5182 1 101 -100 4.615120517 0 -4.615120517 AsmA-like C-terminal region;hypothetical protein;outer membrane assembly protein 140 outer membrane assembly protein WP_005779293.1 UPI0001B49DAD UniRef100_A0A7D4FUA6,UniRef50_I9R8B7,UniRef90_Q5LIK6

group_5126 1 101 -100 4.615120517 0 -4.615120517 Isopentenyl-diphosphate delta-isomerase 140 Isopentenyl-diphosphate delta-isomerase UniRef50_W4PHR9,UniRef90_A0A0K6BQZ1

group_5097 1.180505415 100.2857143 -99.10520887 4.608023255 0.165942665 -4.44208059 hypothetical protein;Magnesium-transporting ATPase P-type 1;magnesium-translocating P-type ATPase 140 magnesium-translocating P-type ATPase UniRef50_P22036,UniRef90_A0A0I9S9Z0

group_5038 1 101 -100 4.615120517 0 -4.615120517 cellulase;hypothetical protein 140 cellulase WP_233484411.1 UPI001F18CDF4 UniRef100_UPI001F18CDF4,UniRef50_A0A108T8E5,UniRef90_A0A0I9S972

group_5004 1 101 -100 4.615120517 0 -4.615120517 hypothetical protein;phage tail protein 140 phage tail protein WP_005776590.1 UPI0001B4A5D8 UniRef100_A0A7D4JQ97,UniRef50_R6Z9S9,UniRef90_A0A4P8LQ15

group_4921 1 101 -100 4.615120517 0 -4.615120517 hypothetical protein;TonB-dep-Rec domain-containing protein;SusC/RagA family TonB-linked outer membrane protein 140 SusC/RagA family TonB-linked outer membrane protein UniRef50_U6RBW6,UniRef90_A0A0E2ALY5

group_4800 1 101 -100 4.615120517 0 -4.615120517 DUF3737 domain-containing protein;hypothetical protein 140 DUF3737 domain-containing protein WP_005777594.1 UPI0001B4AA6F UniRef100_A0A081UKQ9,UniRef50_U2QGD5,UniRef90_A0A081UKQ9

group_4782 1 101 -100 4.615120517 0 -4.615120517 ABC transporter permease;hypothetical protein;FtsX-like permease family 140 FtsX-like permease family WP_005777486.1 UPI0001B4A826 UniRef100_A0A7D4K461,UniRef50_A0A3E5CPD7,UniRef90_A0A3E5CPD7

group_4736 1 101 -100 4.615120517 0 -4.615120517 hypothetical protein;two-component sensor histidine kinase 140 two-component sensor histidine kinase WP_005778433.1 UPI0001B4A3BB UniRef100_A0A7D4GIR7,UniRef50_K1L0Q7,UniRef90_A0A0I9RKI5

group_4693 1 101 -100 4.615120517 0 -4.615120517 metallophosphatase;hypothetical protein 140 metallophosphatase WP_195341219.1 UPI001897E59B UniRef100_UPI001897E59B,UniRef50_K9EMP0,UniRef90_K1FZ83

group_4652 1 101 -100 4.615120517 0 -4.615120517 hypothetical protein 140 hypothetical protein WP_232303471.1 UPI0002F7F83F UniRef100_A0A413JU98,UniRef50_A0A081UL72,UniRef90_A0A081UL72

group_4604 1 101 -100 4.615120517 0 -4.615120517 Beta-galactosidase;hypothetical protein;beta-galactosidase 140 beta-galactosidase WP_005776527.1 UPI0001B4A384 UniRef100_A0A7D4FSW0,UniRef50_A0A174RLX4,UniRef90_A0A0K6BSQ6

group_4473 1 101 -100 4.615120517 0 -4.615120517 Glycerate kinase;Glycerate kinase family protein;glycerate kinase;hypothetical protein 140 glycerate kinase WP_005778896.1 UPI0001B48F2D UniRef100_A0A7D4G879,UniRef50_A0A1S8M7Q6,UniRef90_A0A0I9SBV8

group_4236 1 101 -100 4.615120517 0 -4.615120517 beta-galactosidase;hypothetical protein 140 beta-galactosidase WP_032541831.1 UPI0001B4A65C UniRef100_A0A7D4GET9,UniRef50_A0A6N2ZXA7,UniRef90_A0A0I9UM83

group_4098 1 101 -100 4.615120517 0 -4.615120517 hypothetical protein;NAD-dependent protein deacylase 140 NAD-dependent protein deacylase UniRef50_Q8A3H9,UniRef90_R5RZ55

group_4056 1 101 -100 4.615120517 0 -4.615120517 hypothetical protein 140 hypothetical protein UniRef50_R5R9E7,UniRef90_R5R9E7

group_4055 1 101 -100 4.615120517 0 -4.615120517 hypothetical protein;phosphoesterase 140 phosphoesterase WP_005781325.1 UPI0001B49DEE UniRef100_A0A7D4FTZ9,UniRef50_R5R863,UniRef90_R5R863

group_3941 1 101 -100 4.615120517 0 -4.615120517 zinc-binding dehydrogenase 140 zinc-binding dehydrogenase WP_005777591.1 UPI0001B4AA6D UniRef100_A0A7D4K5D2,UniRef50_A0A1R4FQY6,UniRef90_U6RN69

group_3891 1 101 -100 4.615120517 0 -4.615120517 hypothetical protein;ATPase 140 hypothetical protein

group_3888 1 101 -100 4.615120517 0 -4.615120517 hypothetical protein 140 hypothetical protein WP_005781470.1 UPI0001B4A42C UniRef100_A0A2K9HCN6,UniRef50_A0A0K6BY82,UniRef90_A0A0I9RM65

group_3838 1 101 -100 4.615120517 0 -4.615120517 HTH araC/xylS-type domain-containing protein;hypothetical protein 140 HTH araC/xylS-type domain-containing protein UniRef50_A0A0I9SAW3,UniRef90_A0A0I9SAW3

group_3154 1 101 -100 4.615120517 0 -4.615120517 alpha/beta fold hydrolase;DLH domain-containing protein;hypothetical protein;Serine aminopeptidase S33 140 Serine aminopeptidase, S33 UniRef50_Q5LH43,UniRef90_A0A3E5CKU4

psd 1 101 -100 4.615120517 0 -4.615120517 Phosphatidylserine decarboxylase proenzyme;phosphatidylserine decarboxylase family protein 140 phosphatidylserine decarboxylase family protein WP_005775393.1 UPI0001EC3F50 UniRef100_A0A081UA67,UniRef50_Q8A5K8,UniRef90_Q5LHJ3

group_2276 1 101 -100 4.615120517 0 -4.615120517 zinc ABC transporter substrate-binding protein 140 zinc ABC transporter substrate-binding protein UniRef50_R6JBJ1,UniRef90_A0A2K9H306

murB 1 101 -100 4.615120517 0 -4.615120517 hypothetical protein;UDP-N-acetylmuramate dehydrogenase 140 UDP-N-acetylmuramate dehydrogenase UniRef50_Q8A806,UniRef90_A0A0I9RNA9

group_2140 1 101 -100 4.615120517 0 -4.615120517 Glycosyltransferase group 2 family protein;Glyco-trans-2-like domain-containing protein;glycosyl transferase 140 glycosyl transferase WP_193684342.1 UPI00187AF42A UniRef100_UPI00187AF42A,UniRef50_K6A633,UniRef90_A0A2K9H2M3

group_1986 1 101 -100 4.615120517 0 -4.615120517 SAM-dependent methyltransferase;Ubiquinone/menaquinonebiosynthesismethyltransfer ase UBIE 140 SAM-dependent methyltransferase UniRef50_R5RK05,UniRef90_R5RK05

group_1868 1 101 -100 4.615120517 0 -4.615120517 X-Pro dipeptidyl-peptidase (S15 family) 140 X-Pro dipeptidyl-peptidase (S15 family) WP_005777592.1 UPI0001B4AA6E UniRef100_A0A7D4G1Y7,UniRef50_A0A1M6Q2T3,UniRef90_A0A2K9H5P2

group_1724 1 101 -100 4.615120517 0 -4.615120517 precorrin-2 C(20)-methyltransferase;hypothetical protein 140 precorrin-2 C(20)-methyltransferase UniRef50_A0A415IDP8,UniRef90_A0A0I9S918

group_1678 1 101 -100 4.615120517 0 -4.615120517 hypothetical protein;ABC transporter permease 140 ABC transporter permease WP_005777963.1 UPI0001B494FE UniRef100_A0A2K9GXX7,UniRef50_A0A396MEZ2,UniRef90_F7LR61

group_1489 1 101 -100 4.615120517 0 -4.615120517 3-deoxy-manno-octulosonate cytidylyltransferase 140 3-deoxy-manno-octulosonate cytidylyltransferase WP_005777647.1 UPI0001B4AC8C UniRef100_A0A081UED7,UniRef50_G8UMR9,UniRef90_A0A081UED7

group_1400 1 101 -100 4.615120517 0 -4.615120517 DNA-binding response regulator 140 DNA-binding response regulator UniRef50_A0A0G3M1B7,UniRef90_A0A0I9SA43

group_1302 1 101 -100 4.615120517 0 -4.615120517 hypothetical protein 140 hypothetical protein WP_193683435.1 UPI0018793C00 UniRef100_UPI0018793C00,UniRef50_A0A7D4KFL0,UniRef90_A0A7D4KFL0

group_1160 1 101 -100 4.615120517 0 -4.615120517 hypothetical protein 140 hypothetical protein UniRef50_R5R8W5,UniRef90_R5R8W5

group_1052 1 101 -100 4.615120517 0 -4.615120517 hypothetical protein 140 hypothetical protein

group_1025 1 101 -100 4.615120517 0 -4.615120517 class I SAM-dependent methyltransferase 140 class I SAM-dependent methyltransferase UPI00033824E7 UniRef100_R5S8X6,UniRef50_A0A0I9S4J8,UniRef90_A0A0I9S4J8

group_919 1 101 -100 4.615120517 0 -4.615120517 DUF6078 domain-containing protein 140 DUF6078 domain-containing protein WP_005806583.1 UPI00028089CD UniRef100_A0A081UK25,UniRef50_A0A0P0FST5,UniRef90_A0A0I9SA27

group_912 1 101 -100 4.615120517 0 -4.615120517 protease 140 protease UniRef50_D1ARG0,UniRef90_A0A0I9SAH9

group_19451 1 100.2857143 -99.28571429 4.608023255 0 -4.608023255 DUF6486 domain-containing protein 139 DUF6486 domain-containing protein WP_005807693.1 UPI0001B4A654 UniRef100_R5REL7,UniRef50_R5REL7,UniRef90_R5REL7

group_19087 1 100.2857143 -99.28571429 4.608023255 0 -4.608023255 hypothetical protein 139 hypothetical protein UPI0001B4A1B5 UniRef100_R5REJ5,UniRef50_R5REJ5,UniRef90_R5REJ5

group_16759 1 100.2857143 -99.28571429 4.608023255 0 -4.608023255 hypothetical protein 139 hypothetical protein UPI0001B49F7B UniRef100_A0A081UK11,UniRef50_A0A081UK11,UniRef90_A0A081UK11

group_15193 1.180505415 99.57142857 -98.39092316 4.600875262 0.165942665 -4.434932597 GNAT family N-acetyltransferase 139 GNAT family N-acetyltransferase WP_032541803.1 UPI0001B48F6C UniRef100_A0A0N9DZD1,UniRef50_A0A650LRT6,UniRef90_C5J4Z6

group_14388 1 100.2857143 -99.28571429 4.608023255 0 -4.608023255 DUF3575 domain-containing protein 139 DUF3575 domain-containing protein WP_032541937.1 UPI0001B48F9F UniRef100_A0A7D4KYM6,UniRef50_A0A3E5CLU1,UniRef90_A0A4V1ETQ7

group_13384 1.36101083 98.85714286 -97.49613203 4.593675807 0.308227681 -4.285448125 arylsulfatase;Sulfatase domain-containing protein;hypothetical protein;Arylsulfatase 139 arylsulfatase UniRef50_A0A174K3E6,UniRef90_A0A139KL75

group_12861 3.888086643 88.85714286 -84.96905621 4.487029944 1.357917171 -3.129112773 Fimbrillin-like;Putative lipoprotein;fimbrillin family protein;Fimbrillin family protein 139 Fimbrillin-like WP_008660411.1 UPI0001AFBA5F UniRef100_UPI0001AFBA5F,UniRef50_E1WP80,UniRef90_UPI0001AFBA5F

group_9386 1 100.2857143 -99.28571429 4.608023255 0 -4.608023255 HmuY protein 139 HmuY protein UniRef50_G5GAZ1,UniRef90_A0A0I9RLW6

group_9355 1 100.2857143 -99.28571429 4.608023255 0 -4.608023255 hypothetical protein 139 hypothetical protein WP_122296212.1 UPI000F000875 UniRef100_UPI000F000875,UniRef50_A0A0I9RZ50,UniRef90_A0A0I9RZ50

group_7555 1 100.2857143 -99.28571429 4.608023255 0 -4.608023255 RagB/SusD family nutrient uptake outer membrane protein;hypothetical protein 139 RagB/SusD family nutrient uptake outer membrane protein WP_005818890.1 UPI000282561B UniRef100_A0A5E8KEB2,UniRef50_E5X1B9,UniRef90_A0A0I9SCL2

group_7485 1 100.2857143 -99.28571429 4.608023255 0 -4.608023255 ABC transporter permease;hypothetical protein 139 ABC transporter permease WP_195317088.1 UPI00189B50DF UniRef100_UPI00189B50DF,UniRef50_Q5LFF4,UniRef90_A0A0I9RS21

group_7354 1 100.2857143 -99.28571429 4.608023255 0 -4.608023255 HTH araC/xylS-type domain-containing protein;hypothetical protein 139 HTH araC/xylS-type domain-containing protein UniRef50_I8XU37,UniRef90_R5R7F0

group_7301 1 100.2857143 -99.28571429 4.608023255 0 -4.608023255 Plug domain-containing protein;SusC/RagA family protein;SusC/RagA family TonB-linked outer membrane protein;TonB-dependent receptor plug domain protein;hypothetical protein 139 SusC/RagA family protein UPI000EFB6D59 UniRef100_A0A412Y1G3,UniRef50_A0A174MEE6,UniRef90_A0A0I9SCQ4

group_7243 1 100.2857143 -99.28571429 4.608023255 0 -4.608023255 hypothetical protein 139 hypothetical protein

group_7184 1 100.2857143 -99.28571429 4.608023255 0 -4.608023255 ATP-dependent DNA helicase 139 hypothetical protein

group_6801 1 100.2857143 -99.28571429 4.608023255 0 -4.608023255 MarR family transcriptional regulator 139 MarR family transcriptional regulator WP_044302196.1 UPI0005C7D9CA UniRef100_A0A0I9S4P0,UniRef50_I8XS19,UniRef90_A0A0K6BQN7

group_6580 1 100.2857143 -99.28571429 4.608023255 0 -4.608023255 hypothetical protein;Glycoside hydrolase family 2;glycoside hydrolase family 2 139 glycoside hydrolase family 2 WP_005775993.1 UPI0001B4A5A2 UniRef100_A0A7D4JJ67,UniRef50_A0A0F5JJ95,UniRef90_A0A853PP25

group_6579 1 100.2857143 -99.28571429 4.608023255 0 -4.608023255 DNA-binding protein 139 DNA-binding protein WP_005775997.1 UPI0001B4A5A5 UniRef100_A0A2K9H0V3,UniRef50_R9I0F9,UniRef90_A0A0I9S1F1

group_5929 1 100.2857143 -99.28571429 4.608023255 0 -4.608023255 hypothetical protein;Crp/Fnr family transcriptional regulator 139 Crp/Fnr family transcriptional regulator UniRef50_A0A0F5JP33,UniRef90_A0A0I9SCM9

group_5853 1 100.2857143 -99.28571429 4.608023255 0 -4.608023255 hypothetical protein;PAS domain-containing sensor histidine kinase;Histidine kinase domain-containing protein;HAMP domain-containing histidine kinase 139 Histidine kinase domain-containing protein UniRef50_R5P0J3,UniRef90_A0A2K9H1U3

group_5124 1 100.2857143 -99.28571429 4.608023255 0 -4.608023255 adenosylcobinamide-GDP ribazoletransferase 139 adenosylcobinamide-GDP ribazoletransferase UniRef50_F3PFA5,UniRef90_A0A081UDZ0

group_4871 1 100.2857143 -99.28571429 4.608023255 0 -4.608023255 ABC transporter permease;hypothetical protein 139 ABC transporter permease WP_005819146.1 UPI000282484B UniRef100_UPI000282484B,UniRef50_A0A174NND5,UniRef90_A0A0I9TS07

group_4847 1 100.2857143 -99.28571429 4.608023255 0 -4.608023255 S41 family peptidase;hypothetical protein;Peptidase family S41 139 Peptidase family S41 WP_065739643.1 UPI000810AC59 UniRef100_UPI000810AC59,UniRef50_F7LQL2,UniRef90_A0A0I9SCU5

group_4669 1 100.2857143 -99.28571429 4.608023255 0 -4.608023255 25-diketo-D-gluconic acid reductase;25-diketo-D-gluconic acid reductase A;Aldo-ket-red domain-containing protein 139 2,5-diketo-D-gluconic acid reductase UniRef50_F5VY58,UniRef90_A0A0I9RTB1

group_4524 1 100.2857143 -99.28571429 4.608023255 0 -4.608023255 SOS response-associated peptidase;DUF159 domain-containing protein;hypothetical protein 139 DUF159 domain-containing protein WP_005777661.1 UPI0001EC3D9A UniRef100_A0A413JWU7,UniRef50_H1DDU9,UniRef90_A0A0I9SDK1

group_4506 1 100.2857143 -99.28571429 4.608023255 0 -4.608023255 Sensor histidine kinase;hypothetical protein;Two-component system histidine kinase 139 Two-component system histidine kinase WP_171810386.1 UPI0001B4AB2C UniRef100_A0A7D4FR09,UniRef50_R9HS65,UniRef90_R5REC7

group_4208 1 100.2857143 -99.28571429 4.608023255 0 -4.608023255 TonB-dependent receptor;hypothetical protein 139 TonB-dependent receptor WP_032541374.1 UPI0001B4A5D4 UniRef100_A0A7D4JE45,UniRef50_A0A7Z8YCW7,UniRef90_A0A0I9SCV6

group_4111 1 100.2857143 -99.28571429 4.608023255 0 -4.608023255 SAM-dependent methyltransferase;hypothetical protein;Methyltransf-11 domain-containing protein;Dimethylglycine N-methyltransferase 139 SAM-dependent methyltransferase WP_005776720.1 UPI0001B4AE6B UniRef100_A0A081TXH9,UniRef50_A0A644YEM7,UniRef90_A0A0I9SDN8

group_4108 1 100.2857143 -99.28571429 4.608023255 0 -4.608023255 AraC family transcriptional regulator 139 AraC family transcriptional regulator WP_181996467.1 UPI0015F724F7 UniRef100_A0A412Y0L8,UniRef50_I8X0Z7,UniRef90_A0A015XEE0

group_3932 1 100.2857143 -99.28571429 4.608023255 0 -4.608023255 GNAT family N-acetyltransferase;YoaP domain-containing protein;hypothetical protein 139 GNAT family N-acetyltransferase WP_005809603.1 UPI0001B4AA26 UniRef100_A0A0I9U9A0,UniRef50_C8WK25,UniRef90_A0A0I9SCY8

group_3433 1 100.2857143 -99.28571429 4.608023255 0 -4.608023255 DUF4903 domain-containing protein;hypothetical protein 139 DUF4903 domain-containing protein WP_005776587.1 UPI0001B4A5D5 UniRef100_A0A7D4K6G0,UniRef50_G1W863,UniRef90_A0A0I9SCR4

cdr 1 100.2857143 -99.28571429 4.608023255 0 -4.608023255 NADH dehydrogenase;pyridine nucleotide-disulfide oxidoreductase 139 pyridine nucleotide-disulfide oxidoreductase WP_005778806.1 UPI0001B4AEAC UniRef100_A0A7D4GLR2,UniRef50_Q5LHV0,UniRef90_Q5LHV0

group_2545 1 100.2857143 -99.28571429 4.608023255 0 -4.608023255 AraC family transcriptional regulator 139 AraC family transcriptional regulator WP_005775995.1 UPI0001EC3EE5 UniRef100_A0A081TWT8,UniRef50_R5BZ50,UniRef90_A0A081TWT8

group_2212 1 100.2857143 -99.28571429 4.608023255 0 -4.608023255 hypothetical protein 139 hypothetical protein UniRef50_R5RDQ5,UniRef90_R5RDQ5

group_2153 1 100.2857143 -99.28571429 4.608023255 0 -4.608023255 AraC family transcriptional regulator 139 AraC family transcriptional regulator UniRef50_I8XIG6,UniRef90_R5RBM9

group_2152 1 100.2857143 -99.28571429 4.608023255 0 -4.608023255 Cupin domain-containing protein 139 Cupin domain-containing protein WP_032541344.1 UPI000516E707 UniRef100_A0A7D4KKS5,UniRef50_I3YR46,UniRef90_A0A3E5CYF9

group_2075 1 100.2857143 -99.28571429 4.608023255 0 -4.608023255 hypothetical protein;DUF4373 domain-containing protein 139 DUF4373 domain-containing protein UniRef50_A0A2K9H2E0,UniRef90_A0A2K9H2E0

group_1869 1 100.2857143 -99.28571429 4.608023255 0 -4.608023255 NADPH-dependent FMN reductase 139 NADPH-dependent FMN reductase UniRef50_G5HB24,UniRef90_I9GS78

group_1752 1 100.2857143 -99.28571429 4.608023255 0 -4.608023255 cyclic nucleotide-binding protein 139 cyclic nucleotide-binding protein UniRef50_R5V6K1,UniRef90_A0A0I9S829

group_1599 1 100.2857143 -99.28571429 4.608023255 0 -4.608023255 hypothetical protein 139 hypothetical protein UniRef50_R5RQH4,UniRef90_R5RQH4

group_1474 1 100.2857143 -99.28571429 4.608023255 0 -4.608023255 DUF2807 domain-containing protein 139 DUF2807 domain-containing protein WP_005819158.1 UPI0002824987 UniRef100_A0A0I9SCT9,UniRef50_I9BKV9,UniRef90_A0A0I9SCT9

group_1464 1 100.2857143 -99.28571429 4.608023255 0 -4.608023255 ABC transporter permease;hypothetical protein 139 ABC transporter permease UniRef50_F7LNA6,UniRef90_D1JKG4

group_1137 1 100.2857143 -99.28571429 4.608023255 0 -4.608023255 N-acetylgalactosamine-6-sulfatase 139 N-acetylgalactosamine-6-sulfatase UniRef50_A0A6I6JWF3,UniRef90_R5RX47

group_1069 1 100.2857143 -99.28571429 4.608023255 0 -4.608023255 hypothetical protein 139 hypothetical protein UniRef50_R5RJX9,UniRef90_R5RJX9

group_1066 1.180505415 99.57142857 -98.39092316 4.600875262 0.165942665 -4.434932597 hypothetical protein 139 hypothetical protein UniRef50_E1WVP2,UniRef90_A0A081U755

group_977 1 100.2857143 -99.28571429 4.608023255 0 -4.608023255 hypothetical protein 139 hypothetical protein UniRef50_A0A2K9H3R2,UniRef90_A0A2K9H3R2

group_976 1 100.2857143 -99.28571429 4.608023255 0 -4.608023255 hypothetical protein;acyltransferase 139 acyltransferase WP_005776051.1 UPI0001B4A7F2 UniRef100_A0A0I9RQD0,UniRef50_K5ZM26,UniRef90_A0A0I9RQD0

cobM 1 100.2857143 -99.28571429 4.608023255 0 -4.608023255 precorrin-4 C(11)-methyltransferase 139 precorrin-4 C(11)-methyltransferase WP_005777988.1 UPI0001B4950D UniRef100_A0A7D4FPK1,UniRef50_A0A0P0M3U2,UniRef90_A0A0I9SA66

group_15158 1 99.57142857 -98.57142857 4.600875262 0 -4.600875262 acetyltransferase;hypothetical protein 138 acetyltransferase UniRef50_A0A031WJH2,UniRef90_A0A0I9UPG6

group_12915 3.888086643 88.14285714 -84.2547705 4.478958875 1.357917171 -3.121041704 Fimbrillin-like;hypothetical protein;fimbrillin family protein;Fimbrillin family protein 138 Fimbrillin-like UniRef50_E1WP79,UniRef90_UPI001CE0C7EA

group_10307 7.498194946 73.14285714 -65.6446622 4.292414476 2.014662318 -2.277752158 hypothetical protein;GxxExxY protein 138 GxxExxY protein UniRef50_A0A5C9BJE6,UniRef90_A0A0I9URG0

group_8980 1.180505415 98.85714286 -97.67663744 4.593675807 0.165942665 -4.427733142 hypothetical protein 138 hypothetical protein

uraH 1 99.57142857 -98.57142857 4.600875262 0 -4.600875262 hypothetical protein;hydroxyisourate hydrolase 138 hydroxyisourate hydrolase WP_220576382.1 UPI001C730795 UniRef100_UPI001C730795,UniRef50_A0A0I9SCY7,UniRef90_A0A0I9SCY7

group_7603 1 99.57142857 -98.57142857 4.600875262 0 -4.600875262 hypothetical protein 138 hypothetical protein UniRef50_A0A396C043,UniRef90_A0A396C043

group_6648 1 99.57142857 -98.57142857 4.600875262 0 -4.600875262 flavin oxidoreductase 138 flavin oxidoreductase WP_223126380.1 UPI001CA7EC57 UniRef100_UPI001CA7EC57,UniRef50_G4Q2K5,UniRef90_I9GS91

group_5516 1 99.57142857 -98.57142857 4.600875262 0 -4.600875262 hypothetical protein 138 hypothetical protein UniRef50_R5RD15,UniRef90_R5RD15

group_5078 1 99.57142857 -98.57142857 4.600875262 0 -4.600875262 hypothetical protein;DUF3244 domain-containing protein 138 DUF3244 domain-containing protein WP_005781727.1 UPI0001EC4050 UniRef100_A0A081UF39,UniRef50_R5RTQ5,UniRef90_R5RTQ5

group_4819 1 99.57142857 -98.57142857 4.600875262 0 -4.600875262 DNA primase/helicase;DNA primase;hypothetical protein;toprim domain-containing protein 138 DNA primase UniRef50_R5RQX9,UniRef90_R5RQX9

group_4805 1 99.57142857 -98.57142857 4.600875262 0 -4.600875262 hypothetical protein;6-bladed beta-propeller 138 6-bladed beta-propeller WP_032539113.1 UPI0004D82025 UniRef100_A0A4P8L9Q2,UniRef50_F3PQS2,UniRef90_A0A0I9UR87

group_2918 1 99.57142857 -98.57142857 4.600875262 0 -4.600875262 AraC family transcriptional regulator 138 AraC family transcriptional regulator WP_122143605.1 UPI000EFAAC03 UniRef100_A0A412XRH5,UniRef50_A0A3E5GAI6,UniRef90_A0A081TV41

group_2459 1 99.57142857 -98.57142857 4.600875262 0 -4.600875262 Membrane protein;hypothetical protein 138 Membrane protein WP_032541373.1 UPI0002D8EBA9 UniRef100_A0A7D4JIL4,UniRef50_F9D0M4,UniRef90_A0A0I9SCJ4

group_1972 1 99.57142857 -98.57142857 4.600875262 0 -4.600875262 RagB/SusD family nutrient uptake outer membrane protein 138 RagB/SusD family nutrient uptake outer membrane protein WP_005811892.1 UPI0001B4931E UniRef100_A0A0I9U4F7,UniRef50_A0A1I5WNP6,UniRef90_A0A0I9U4F7

group_1648 1 99.57142857 -98.57142857 4.600875262 0 -4.600875262 hypothetical protein;tryptophan--tRNA ligase 138 tryptophan--tRNA ligase WP_195317407.1 UPI00189B6ECA UniRef100_UPI00189B6ECA,UniRef90_J9DD46

group_1397 1 99.57142857 -98.57142857 4.600875262 0 -4.600875262 DUF4933 domain-containing protein 138 DUF4933 domain-containing protein UniRef50_Q5LDL6,UniRef90_A0A0I9USG3

group_1213 1 99.57142857 -98.57142857 4.600875262 0 -4.600875262 hypothetical protein;PorT family protein 138 PorT family protein UniRef50_E6SS99,UniRef90_K1FV43

group_1141 1 99.57142857 -98.57142857 4.600875262 0 -4.600875262 TonB-dependent receptor;SusC/RagA family protein 138 SusC/RagA family protein WP_032541610.1 UPI0001B4931F UniRef100_A0A413K1L7,UniRef50_A0A0P0FPD8,UniRef90_A0A0I9SC06

group_1138 1 99.57142857 -98.57142857 4.600875262 0 -4.600875262 DUF4248 domain-containing protein 138 DUF4248 domain-containing protein UniRef50_A0A069CZM0,UniRef90_A0A081U030

group_1086 1 99.57142857 -98.57142857 4.600875262 0 -4.600875262 hypothetical protein 138 hypothetical protein WP_122141911.1 UPI000EFD7FB3 UniRef100_A0A412YJT1,UniRef50_A0A0I9SCL1,UniRef90_A0A0I9SCL1

group_14378 1 98.85714286 -97.85714286 4.593675807 0 -4.593675807 hypothetical protein 137 hypothetical protein UPI0002808796 UniRef100_R5RX34,UniRef50_R5RX34,UniRef90_R5RX34

group_6906 1 98.85714286 -97.85714286 4.593675807 0 -4.593675807 hypothetical protein;DUF4373 domain-containing protein 137 DUF4373 domain-containing protein UniRef50_A0A081TVC6,UniRef90_A0A081TVC6

group_5626 1.180505415 98.14285714 -96.96235173 4.586424143 0.165942665 -4.420481478 hypothetical protein 137 hypothetical protein

group_5611 1 98.85714286 -97.85714286 4.593675807 0 -4.593675807 Phenylalanine--tRNA ligase beta subunit 137 Phenylalanine--tRNA ligase beta subunit WP_005778115.1 UPI0001B49932 UniRef100_A0A7D4G1N9,UniRef50_Q5LC76,UniRef90_Q5LC76

group_3783 1 98.85714286 -97.85714286 4.593675807 0 -4.593675807 ribonucleotide-diphosphate reductase subunit beta 137 ribonucleotide-diphosphate reductase subunit beta WP_005776743.1 UPI0001B4AE80 UniRef100_A0A2K9H580,UniRef50_O83092,UniRef90_Q5LBV8

group_3707 1 98.85714286 -97.85714286 4.593675807 0 -4.593675807 hypothetical protein 137 hypothetical protein UniRef50_A0A2K9H469,UniRef90_A0A2K9H469

group_2983 1 98.85714286 -97.85714286 4.593675807 0 -4.593675807 TSCPD domain-containing protein 137 TSCPD domain-containing protein WP_044300173.1 UPI0005C7D7D6 UniRef100_A0A0I9SA20,UniRef50_S0F5Y4,UniRef90_A0A0K6BUU2

group_1910 1 98.85714286 -97.85714286 4.593675807 0 -4.593675807 hypothetical protein;ribonucleoside-diphosphate reductase subunit alpha 137 ribonucleoside-diphosphate reductase subunit alpha WP_005776742.1 UPI0001B4AE7F UniRef100_A0A7D4G6M6,UniRef50_O83972,UniRef90_A0A081TXJ7

group_1043 1 98.85714286 -97.85714286 4.593675807 0 -4.593675807 hypothetical protein 137 hypothetical protein UniRef50_R5RGH2,UniRef90_R5RGH2

group_16906 1 98.14285714 -97.14285714 4.586424143 0 -4.586424143 hypothetical protein 136 hypothetical protein WP_005778111.1 UPI0001B4992F UniRef100_A0A2K9GY40,UniRef50_I9S274,UniRef90_A0A017N8S1

group_12372 1 98.14285714 -97.14285714 4.586424143 0 -4.586424143 hypothetical protein 136 hypothetical protein WP_005776667.1 UPI0001B4AA4A UniRef100_A0A7D4G6Q4,UniRef50_A0A0I9SCN7,UniRef90_A0A0I9SCN7

group_9689 1 98.14285714 -97.14285714 4.586424143 0 -4.586424143 hypothetical protein;FtsX-like permease family protein;permease 136 permease WP_005777826.1 UPI0001B49105 UniRef100_A0A2K9H907,UniRef50_A0A1M6FWH9,UniRef90_A0A0I9S8L7

group_9536 1 98.14285714 -97.14285714 4.586424143 0 -4.586424143 LruC domain-containing protein 136 LruC domain-containing protein WP_220576886.1 UPI001C73175B UniRef100_UPI001C73175B,UniRef50_A0A412Y5N3,UniRef90_A0A412Y5N3

group_7461 1 98.14285714 -97.14285714 4.586424143 0 -4.586424143 hypothetical protein 136 hypothetical protein

group_7102 1.180505415 97.42857143 -96.24806601 4.579119509 0.165942665 -4.413176844 hypothetical protein 136 hypothetical protein

group_4298 1 98.14285714 -97.14285714 4.586424143 0 -4.586424143 signal peptidase I 136 signal peptidase I WP_005777079.1 UPI0001EC3DA4 UniRef100_A0A7D4FTQ1,UniRef50_A0A0I9S7H7,UniRef90_A0A0I9SBC1

group_3998 1 98.14285714 -97.14285714 4.586424143 0 -4.586424143 SAM-dependent methyltransferase 136 SAM-dependent methyltransferase WP_005775709.1 UPI0001B4949D UniRef100_A0A7D4FUZ4,UniRef50_K0XGK5,UniRef90_A0A174NSH4

group_3327 1 98.14285714 -97.14285714 4.586424143 0 -4.586424143 glycosyl hydrolase family 43 136 glycosyl hydrolase family 43 WP_005822801.1 UPI0001B49B65 UniRef100_A0A081TZB5,UniRef50_A0A1V5YTH8,UniRef90_A0A0K6BNV7

group_2454 3.707581227 87.42857143 -83.7209902 4.470822133 1.310379704 -3.16044243 hypothetical protein 136 hypothetical protein UniRef50_R5RUF9,UniRef90_R5RUF9

group_1856 1 98.14285714 -97.14285714 4.586424143 0 -4.586424143 DUF3256 domain-containing protein 136 DUF3256 domain-containing protein WP_005818533.1 UPI000282497B UniRef100_A0A413K390,UniRef50_A0A0N7IAN6,UniRef90_A0A0I9SBK7

group_1377 1 98.14285714 -97.14285714 4.586424143 0 -4.586424143 Lipocalin-like domain-containing protein 136 Lipocalin-like domain-containing protein UniRef50_E6SP55,UniRef90_A0A0I9UUU7

group_6666 1 97.42857143 -96.42857143 4.579119509 0 -4.579119509 hypothetical protein 135 hypothetical protein WP_022012966.1 UPI000339CBE1 UniRef100_R5RZ63,UniRef50_E6SRM8,UniRef90_A0A0I9TM41

group_6129 1 97.42857143 -96.42857143 4.579119509 0 -4.579119509 hypothetical protein 135 hypothetical protein

group_2862 1 97.42857143 -96.42857143 4.579119509 0 -4.579119509 acyltransferase;Acyltransferase 135 acyltransferase WP_005782922.1 UPI0001B48FA9 UniRef100_A0A0I9S619,UniRef50_K9E9U8,UniRef90_A0A0I9S619

group_1055 1 97.42857143 -96.42857143 4.579119509 0 -4.579119509 hypothetical protein 135 hypothetical protein UniRef50_R5RC77,UniRef90_R5RC77

group_3126 1 96.71428571 -95.71428571 4.571761124 0 -4.571761124 hypothetical protein 134 hypothetical protein UniRef50_R5RKT6,UniRef90_R5RKT6

group_2720 1 96.71428571 -95.71428571 4.571761124 0 -4.571761124 hypothetical protein 134 hypothetical protein

group_19371 1 96 -95 4.564348191 0 -4.564348191 hypothetical protein 133 hypothetical protein

group_7356 1 96 -95 4.564348191 0 -4.564348191 hypothetical protein 133 hypothetical protein

group_1036 2.263537906 91 -88.73646209 4.510859507 0.816929034 -3.693930472 Polysaccharide biosynthesis/export protein 133 Polysaccharide biosynthesis/export protein UniRef50_I8XIQ4,UniRef90_R5RW91

group_12468 1.36101083 93.85714286 -92.49613203 4.541773869 0.308227681 -4.233546188 acetyltransferase;Acetyltransferase 132 acetyltransferase WP_235331750.1 UPI001F17D98D UniRef100_UPI001F17D98D,UniRef50_A0A0P0FBA6,UniRef90_A0A081U0S3

group_7449 2.083032491 91 -88.91696751 4.510859507 0.73382476 -3.777034746 Hydrolase 132 Hydrolase WP_182114648.1 UPI0015F47CE3 UniRef100_UPI0015F47CE3,UniRef50_A0A2M9TSI8,UniRef90_A0A0I9RN40

group_7367 1.902527076 91.71428571 -89.81175864 4.518678155 0.643183042 -3.875495112 hypothetical protein 132 hypothetical protein

group_4783 2.083032491 91 -88.91696751 4.510859507 0.73382476 -3.777034746 capsular biosynthesis protein;Wzz/FepE/Etk N-terminal domain-containing protein 132 capsular biosynthesis protein UniRef50_A0A081TYP0,UniRef90_A0A081TYP0

group_4509 1 95.28571429 -94.28571429 4.556879897 0 -4.556879897 hypothetical protein 132 hypothetical protein UniRef50_A0A0I9SAP8,UniRef90_A0A0I9SAP8

group_4383 1.180505415 94.57142857 -93.39092316 4.549355407 0.165942665 -4.383412742 hypothetical protein 132 hypothetical protein UPI0009B6B5CB UniRef100_A0A4P8MS96,UniRef50_A0A2K9H0I8,UniRef90_A0A2K9H0I8

group_1041 1 95.28571429 -94.28571429 4.556879897 0 -4.556879897 hypothetical protein 132 hypothetical protein UPI00028095AD UniRef100_R5RCI5,UniRef50_Q5LHH7,UniRef90_A0A0I9S4B7

group_13939 5.873646209 74.57142857 -68.69778236 4.311757439 1.770475601 -2.541281838 hypothetical protein 131 hypothetical protein UniRef50_A0A7J5PSU1,UniRef90_F7M6J7

group_3098 1.722021661 91.71428571 -89.99226405 4.518678155 0.543498985 -3.97517917 hypothetical protein 131 hypothetical protein

group_2411 1.36101083 93.14285714 -91.78184631 4.534134413 0.308227681 -4.225906732 hypothetical protein 131 hypothetical protein

group_1343 1 94.57142857 -93.57142857 4.549355407 0 -4.549355407 hypothetical protein 131 hypothetical protein UniRef50_K9E7U6,UniRef90_A0A0I9SAQ5

group_5494 1.180505415 93.14285714 -91.96235173 4.534134413 0.165942665 -4.368191748 alpha-glucosidase;hypothetical protein 130 alpha-glucosidase UniRef50_A0A379MNG1,UniRef90_A0A0I9S739

group_13482 1.36101083 91.71428571 -90.35327488 4.518678155 0.308227681 -4.210450473 Type I phosphodiesterase / nucleotide pyrophosphatase family protein;hypothetical protein;DUF4976 domain-containing protein;Sulfatase domain-containing protein;Sulfatase 129 Type I phosphodiesterase / nucleotide pyrophosphatase family protein UniRef50_R7P8T2,UniRef90_A0A015WYS4

group_7407 1 93.14285714 -92.14285714 4.534134413 0 -4.534134413 hypothetical protein 129 hypothetical protein

sucC 1 93.14285714 -92.14285714 4.534134413 0 -4.534134413 Ligase-CoA domain-containing protein;ADP-forming succinate--CoA ligase subunit beta 129 ADP-forming succinate--CoA ligase subunit beta WP_022012734.1 UPI00033FF315 UniRef100_R5RL42,UniRef50_O28097,UniRef90_Q5LCW4

sucD 1 93.14285714 -92.14285714 4.534134413 0 -4.534134413 succinate--CoA ligase subunit alpha 129 succinate--CoA ligase subunit alpha WP_195316755.1 UPI0018971445 UniRef100_UPI0018971445,UniRef50_A0A0N4V8W0,UniRef90_A0A0K6BU24

group_19450 1 92.42857143 -91.42857143 4.526436145 0 -4.526436145 hypothetical protein 128 hypothetical protein UPI0001B4A8BA UniRef100_R5S827,UniRef50_R5S827,UniRef90_R5S827

group_13481 1.36101083 91 -89.63898917 4.510859507 0.308227681 -4.202631825 RagB/SusD family nutrient uptake outer membrane protein;hypothetical protein;SusD-like-3 domain-containing protein;Outer membrane protein;SusD-RagB domain-containing protein 128 RagB/SusD family nutrient uptake outer membrane protein WP_005777072.1 UPI0001B49B30 UniRef100_A0A0I9SAQ1,UniRef50_C6IQA4,UniRef90_A0A0I9SAQ1

group_13408 2.263537906 87.42857143 -85.16503352 4.470822133 0.816929034 -3.653893099 hypothetical protein;Acetyltransferase;maltose acetyltransferase 128 maltose acetyltransferase UniRef50_Q4BXE7,UniRef90_D1JVP8

group_13392 1.36101083 91 -89.63898917 4.510859507 0.308227681 -4.202631825 Glyco-hydro2-C5 domain-containing protein;Glycosyl hydrolases family 2 sugar binding domain protein;beta-galactosidase;hypothetical protein;Beta-galactosidase 128 beta-galactosidase UniRef50_A0A060R7G5,UniRef90_A0A412XWF1

group_8838 1.180505415 91.71428571 -90.5337803 4.518678155 0.165942665 -4.35273549 hypothetical protein 128 hypothetical protein

group_6876 1 92.42857143 -91.42857143 4.526436145 0 -4.526436145 hypothetical protein 128 hypothetical protein

group_4179 1 92.42857143 -91.42857143 4.526436145 0 -4.526436145 triose-phosphate isomerase 128 triose-phosphate isomerase WP_032531412.1 UPI0004D59A96 UniRef100_A0A4P8LLR6,UniRef50_Q8A0U2,UniRef90_Q8A0U2

group_4079 1 92.42857143 -91.42857143 4.526436145 0 -4.526436145 hypothetical protein;AraR-C domain-containing protein;DNA mismatch repair protein MutT 128 DNA mismatch repair protein MutT WP_005806044.1 UPI0002808B03 UniRef100_A0A081UEB1,UniRef50_K9E9Y7,UniRef90_A0A149NKK0

group_1771 1 92.42857143 -91.42857143 4.526436145 0 -4.526436145 hypothetical protein 128 hypothetical protein UniRef50_R5RC60,UniRef90_R5RC60

group_1361 1 92.42857143 -91.42857143 4.526436145 0 -4.526436145 hypothetical protein 128 hypothetical protein UniRef50_R5RF77,UniRef90_R5RF77

group_13794 1.36101083 90.28571429 -88.92470346 4.502979245 0.308227681 -4.194751564 FecR family protein;hypothetical protein;anti-sigma factor 127 anti-sigma factor WP_227559412.1 UPI000A788877 UniRef100_A0A2K9H9V6,UniRef50_A0A015W7D1,UniRef90_A0A2K9H9V6

group_13778 1.36101083 90.28571429 -88.92470346 4.502979245 0.308227681 -4.194751564 SusC/RagA family protein;hypothetical protein 127 SusC/RagA family protein WP_032594985.1 UPI0004B37C1E UniRef100_A0A015WYQ7,UniRef50_B6VW79,UniRef90_A0A015WYQ7

group_4691 1 91.71428571 -90.71428571 4.518678155 0 -4.518678155 FGGY-N domain-containing protein;L-xylulose/3-keto-L-gulonate kinase;carbohydrate kinase 127 carbohydrate kinase WP_193684137.1 UPI00187ACEE3 UniRef100_UPI00187ACEE3,UniRef50_A0A1W6LKI4,UniRef90_A0A0I9S8E8

group_4355 1 91.71428571 -90.71428571 4.518678155 0 -4.518678155 C2H2-type domain-containing protein;hypothetical protein 127 C2H2-type domain-containing protein WP_005780050.1 UPI0001B4915E UniRef100_A0A081UHK5,UniRef50_A0A081UHK5,UniRef90_A0A081UHK5

thiD 1 91.71428571 -90.71428571 4.518678155 0 -4.518678155 bifunctional hydroxymethylpyrimidine kinase/phosphomethylpyrimidine kinase 127 bifunctional hydroxymethylpyrimidine kinase/phosphomethylpyrimidine kinase WP_188520389.1 UPI00166C9C0C UniRef100_UPI00166C9C0C,UniRef50_A0A0N7IA57,UniRef90_A0A0I9UP48

group_8513 1.902527076 87.42857143 -85.52604435 4.470822133 0.643183042 -3.827639091 Membrane protein;ECF transporter S component 126 Membrane protein WP_032580566.1 UPI00044670BD UniRef100_A0A853PNK9,UniRef50_E5X1T0,UniRef90_Q64N97

group_6538 1 91 -90 4.510859507 0 -4.510859507 CPBP family intramembrane metalloprotease;hypothetical protein 126 CPBP family intramembrane metalloprotease UniRef50_A0A7G2M6U7,UniRef90_A0A081U8E2

group_4846 1 91 -90 4.510859507 0 -4.510859507 hypothetical protein 126 hypothetical protein

group_2002 1 91 -90 4.510859507 0 -4.510859507 hypothetical protein 126 hypothetical protein

group_1080 1.180505415 90.28571429 -89.10520887 4.502979245 0.165942665 -4.33703658 hypothetical protein 126 hypothetical protein UPI0001B4AE70 UniRef100_R5RAT4,UniRef50_R5RAT4,UniRef90_R5RAT4

group_15072 1 90.28571429 -89.28571429 4.502979245 0 -4.502979245 hypothetical protein 125 hypothetical protein WP_101602584.1 UPI000C76EC4F UniRef100_A0A2K9GU87,UniRef50_A0A0I9UHT0,UniRef90_A0A0I9UHT0

group_13799 1.36101083 88.85714286 -87.49613203 4.487029944 0.308227681 -4.178802262 Sigma-70 region 4 family protein;DNA-directed RNA polymerase sigma-70 factor;RNA polymerase sigma-70 factor 125 DNA-directed RNA polymerase sigma-70 factor UniRef50_A0A174PY12

group_13520 1.722021661 87.42857143 -85.70654977 4.470822133 0.543498985 -3.927323149 hypothetical protein;helix-turn-helix transcriptional regulator 125 helix-turn-helix transcriptional regulator WP_165017322.1 UPI0013EA00FE UniRef100_UPI0013EA00FE,UniRef50_K1FWN0,UniRef90_A0A0I9RUP6

group_9147 6.23465704 68.85714286 -62.62248582 4.232033965 1.830123572 -2.401910393 6-bladed beta-propeller 125 6-bladed beta-propeller UniRef50_A0A374VF19,UniRef90_A0A2K9H7W1

group_9021 1 90.28571429 -89.28571429 4.502979245 0 -4.502979245 hypothetical protein 125 hypothetical protein UniRef50_D7J491,UniRef90_D7J491

group_4284 1 90.28571429 -89.28571429 4.502979245 0 -4.502979245 hypothetical protein;AraC family transcriptional regulator 125 AraC family transcriptional regulator UniRef50_A0A0I9UQ99,UniRef90_A0A0I9UQ99

group_3415 1 90.28571429 -89.28571429 4.502979245 0 -4.502979245 hypothetical protein 125 hypothetical protein WP_122143349.1 UPI000EFB693C UniRef100_A0A412XW04,UniRef50_K9DYX8,UniRef90_A0A0I9SDM7

group_1094 4.249097473 77.42857143 -73.17947396 4.349355852 1.446706601 -2.902649251 hypothetical protein 125 hypothetical protein UniRef50_Q64WB7,UniRef90_I9BGD9

group_9925 1.180505415 88.85714286 -87.67663744 4.487029944 0.165942665 -4.321087279 Fimbrillin-like;hypothetical protein 124 Fimbrillin-like UniRef50_A0A4P8LH24,UniRef90_A0A3E5CFD1

group_7077 6.054151625 68.85714286 -62.80299123 4.232033965 1.800744256 -2.431289709 NVEALA protein;hypothetical protein 124 NVEALA protein UniRef50_A0A396C3X1,UniRef90_A0A396C3X1

group_6416 1 89.57142857 -88.57142857 4.495036392 0 -4.495036392 integrase 124 integrase WP_005780046.1 UPI0001B4915B UniRef100_A0A7D4FQR8,UniRef50_A0A0I9S502,UniRef90_A0A081UHK7

group_5648 1.902527076 86 -84.09747292 4.454347296 0.643183042 -3.811164254 SusC/RagA family TonB-linked outer membrane protein;hypothetical protein;TonB-dependent receptor;SusC/RagA family protein 124 SusC/RagA family protein WP_005781049.1 UPI0001EC3FFF UniRef100_A0A7D4JLX5,UniRef50_F7LT23,UniRef90_F7LT23

aslA 1 89.57142857 -88.57142857 4.495036392 0 -4.495036392 N-acetylgalactosamine-6-sulfatase;hypothetical protein 124 N-acetylgalactosamine-6-sulfatase WP_005781597.1 UPI0001B4A8A7 UniRef100_A0A7D4JW34,UniRef50_A0A517MG29,UniRef90_A0A0P0F8Q7

group_1256 1 89.57142857 -88.57142857 4.495036392 0 -4.495036392 MATE family efflux transporter 124 MATE family efflux transporter WP_005776593.1 UPI0001B4A7F9 UniRef100_A0A0I9SCR7,UniRef50_A0A0K6BSP5,UniRef90_A0A0K6BSP5

group_11314 6.23465704 67.42857143 -61.19391439 4.211068837 1.830123572 -2.380945265 hypothetical protein 123 hypothetical protein UniRef50_A0A081UF31,UniRef90_A0A081UF31

group_7371 1.902527076 85.28571429 -83.38318721 4.446006964 0.643183042 -3.802823922 GNAT family N-acetyltransferase 123 GNAT family N-acetyltransferase UniRef50_A0A0I9S819,UniRef90_A0A0I9S819

group_6496 1 88.85714286 -87.85714286 4.487029944 0 -4.487029944 Fimbrillin-like 123 Fimbrillin-like WP_122330425.1 UPI000EE1272E UniRef100_A0A396BZ18,UniRef50_E1WP75,UniRef90_A0A3E5CEX2

group_10373 6.23465704 66.71428571 -60.47962867 4.200419109 1.830123572 -2.370295537 hypothetical protein 122 hypothetical protein WP_032561996.1 UPI000516EF36 UniRef100_A0A642F2R9,UniRef50_A0A0I9S9L2,UniRef90_A0A0I9S9L2

group_9901 1 88.14285714 -87.14285714 4.478958875 0 -4.478958875 Fimbrillin-like 122 Fimbrillin-like UniRef50_E1WP77,UniRef90_A0A0I9S585

group_6383 1 88.14285714 -87.14285714 4.478958875 0 -4.478958875 Fimbrillin-like;Fimbrillin family protein 122 Fimbrillin-like UniRef50_A0A0I9S5I3,UniRef90_A0A0I9S5I3

group_3894 1 88.14285714 -87.14285714 4.478958875 0 -4.478958875 AraC family transcriptional regulator;hypothetical protein 122 AraC family transcriptional regulator WP_005777585.1 UPI0001B4AA69 UniRef100_A0A3E5CTR0,UniRef50_I8XIH8,UniRef90_I8XIH8

group_3304 1 88.14285714 -87.14285714 4.478958875 0 -4.478958875 hypothetical protein 122 hypothetical protein UniRef50_A0A396BWP1,UniRef90_A0A396BWP1

group_2209 1.180505415 87.42857143 -86.24806601 4.470822133 0.165942665 -4.304879469 hypothetical protein 122 hypothetical protein UniRef50_R5RIZ6,UniRef90_R5RIZ6

group_10965 6.054151625 66.71428571 -60.66013409 4.200419109 1.800744256 -2.399674853 hypothetical protein;6-bladed beta-propeller 121 6-bladed beta-propeller WP_005781716.1 UPI0001B4AB13 UniRef100_A0A7D4FW05,UniRef50_A0A0I9S9W8,UniRef90_A0A0I9S9W8

group_6735 1 87.42857143 -86.42857143 4.470822133 0 -4.470822133 Core-binding (CB) domain-containing protein;Integrase/recombinase XerD;Recombinase;Putative tyrosine recombinase;hypothetical protein 121 Recombinase UniRef50_A0A3N2N4J8,UniRef90_A0A3E5CQA1

group_6483 1 87.42857143 -86.42857143 4.470822133 0 -4.470822133 Methylated-DNA--protein-cysteine methyltransferase 121 Methylated-DNA--protein-cysteine methyltransferase UniRef50_A0A142EMA4,UniRef90_A0A0I9S7J8

group_6482 1 87.42857143 -86.42857143 4.470822133 0 -4.470822133 AraC family transcriptional regulator;Regulatory protein of adaptative response;cysteine methyltransferase 121 cysteine methyltransferase WP_032539183.1 UPI0004D62C30 UniRef100_A0A4P8L9Z5,UniRef50_A0A371YTP3,UniRef90_A0A0I9UNC6

group_4202 1 87.42857143 -86.42857143 4.470822133 0 -4.470822133 hypothetical protein 121 hypothetical protein WP_005777638.1 UPI0001B4AC87 UniRef100_A0A7D4K5C5,UniRef50_F3PNN3,UniRef90_A0A0I9SB81

group_3545 1 87.42857143 -86.42857143 4.470822133 0 -4.470822133 mannose-1-phosphate guanylyltransferase 121 mannose-1-phosphate guanylyltransferase WP_195317734.1 UPI0018990C92 UniRef100_UPI0018990C92,UniRef50_A0A449I2Y7,UniRef90_A0A0K6BZE5

group_2189 1 87.42857143 -86.42857143 4.470822133 0 -4.470822133 DUF1349 domain-containing protein 121 DUF1349 domain-containing protein WP_032531587.1 UPI0004D5ECAA UniRef100_A0A4V1ETN4,UniRef50_Q5L815,UniRef90_Q5L815

group_6048 1 86.71428571 -85.71428571 4.462618642 0 -4.462618642 YnfA family protein;Uncharacterised BCR YnfA/UPF0060 family 120 YnfA family protein WP_122141938.1 UPI000EFABC9D UniRef100_A0A412YJV5,UniRef50_Q65MC6,UniRef90_A0A412YJV5

group_2448 1 86.71428571 -85.71428571 4.462618642 0 -4.462618642 hypothetical protein 120 hypothetical protein

group_2079 1 86.71428571 -85.71428571 4.462618642 0 -4.462618642 DUF4248 domain-containing protein 120 DUF4248 domain-containing protein WP_223126558.1 UPI001CA7BC9F UniRef100_UPI001CA7BC9F,UniRef50_A0A081UHK6,UniRef90_A0A081UHK6

group_1727 1 86.71428571 -85.71428571 4.462618642 0 -4.462618642 hypothetical protein 120 hypothetical protein UniRef50_R5RKG5,UniRef90_R5RKG5

group_941 1 86.71428571 -85.71428571 4.462618642 0 -4.462618642 DUF5054 domain-containing protein;hypothetical protein 120 DUF5054 domain-containing protein WP_193683223.1 UPI0018791CE1 UniRef100_UPI0018791CE1,UniRef50_A0A0I9S4V2,UniRef90_A0A0I9S4V2

group_349 2.444043321 81 -78.55595668 4.394449155 0.893653767 -3.500795388 S9 family peptidase 120 S9 family peptidase WP_022010975.1 UPI0001B4955E UniRef100_A0A0I9RQX6,UniRef50_A0A0I9S847,UniRef90_A0A0I9S847

group_10924 2.985559567 78.14285714 -75.15729758 4.358538653 1.093787189 -3.264751465 DUF4469 domain-containing protein;hypothetical protein 119 DUF4469 domain-containing protein WP_005821496.1 UPI000282565D UniRef100_A0A413K2X4,UniRef50_I9B7H3,UniRef90_I9B7H3

group_7133 1 85.28571429 -84.28571429 4.446006964 0 -4.446006964 6-bladed beta-propeller 118 6-bladed beta-propeller UniRef50_W4PMG2,UniRef90_A0A0I9UMF0

group_6392 1 85.28571429 -84.28571429 4.446006964 0 -4.446006964 Putative glycosyltransferase;glycosyl transferase 118 Putative glycosyltransferase UniRef50_Q5LIR2

group_5674 1 85.28571429 -84.28571429 4.446006964 0 -4.446006964 hypothetical protein 118 hypothetical protein UniRef50_A0A2K9GZ47,UniRef90_A0A2K9GZ47

group_1638 1 85.28571429 -84.28571429 4.446006964 0 -4.446006964 hypothetical protein;glycosyl transferase 118 glycosyl transferase WP_005779398.1 UPI0001B49FB7 UniRef100_A0A2K9H3B2,UniRef50_Q5LIR2,UniRef90_A0A2K9H3B2

group_10660 2.263537906 79.57142857 -77.30789067 4.376655091 0.816929034 -3.559726056 hypothetical protein 117 hypothetical protein UniRef50_A0A081UJP8,UniRef90_A0A081UJP8

group_7081 1 84.57142857 -83.57142857 4.437596486 0 -4.437596486 hypothetical protein;Glycosyl transferase family 4 117 Glycosyl transferase family 4 WP_171810371.1 UPI0002F078A7 UniRef100_A0A7D4FSB3,UniRef50_A0A174CUL6,UniRef90_A0A081U3L9

group_6448 1 84.57142857 -83.57142857 4.437596486 0 -4.437596486 AraC family transcriptional regulator 117 AraC family transcriptional regulator WP_122143119.1 UPI000EFB3D28 UniRef100_A0A412Y0I8,UniRef50_A0A081UBH5,UniRef90_A0A081UBH5

group_4507 1 84.57142857 -83.57142857 4.437596486 0 -4.437596486 hypothetical protein 117 hypothetical protein WP_122143120.1 UPI000EFDA30E UniRef100_A0A412XZV0,UniRef50_W0ESU1,UniRef90_A0A0I9S8L1

gldL 1 84.57142857 -83.57142857 4.437596486 0 -4.437596486 gliding motility protein GldL 117 gliding motility protein GldL UniRef50_B5CYX0,UniRef90_A0A0K6BVJ8

group_10310 2.985559567 76 -73.01444043 4.33073334 1.093787189 -3.236946151 recombinase;hypothetical protein 116 recombinase WP_005802503.1 UPI0002690162 UniRef100_I9VJI1,UniRef50_A0A0F5JTI0,UniRef90_I9VJI1

group_8736 1 83.85714286 -82.85714286 4.429114671 0 -4.429114671 hypothetical protein 116 hypothetical protein UniRef50_A0A015VHT8,UniRef90_A0A2K9GZR8

group_2058 1.180505415 83.14285714 -81.96235173 4.420560299 0.165942665 -4.254617634 hypothetical protein 116 hypothetical protein

group_7105 1 83.14285714 -82.14285714 4.420560299 0 -4.420560299 Epoxyqueuosine reductase;[Fe-S]-binding protein 115 Epoxyqueuosine reductase UniRef50_A0A0P0EYG3,UniRef90_A0A412XWH6

group_5995 1.722021661 79.57142857 -77.84940691 4.376655091 0.543498985 -3.833156106 hypothetical protein 114 hypothetical protein

group_3866 1 82.42857143 -81.42857143 4.411932117 0 -4.411932117 hypothetical protein 114 hypothetical protein WP_032540224.1 UPI0004D55408 UniRef100_A0A4V1ETC6,UniRef50_A0A0I9S8X6,UniRef90_A0A0I9S8X6

group_2224 1.36101083 81 -79.63898917 4.394449155 0.308227681 -4.086221473 hypothetical protein 114 hypothetical protein

group_7307 1 81.71428571 -80.71428571 4.403228842 0 -4.403228842 Transcriptional regulator;transcriptional regulator 113 transcriptional regulator WP_005778094.1 UPI0001B49926 UniRef100_A0A081U173,UniRef50_A0A0P0G1R2,UniRef90_A0A081U173

group_4401 1 81.71428571 -80.71428571 4.403228842 0 -4.403228842 Peptidase-M56 domain-containing protein;hypothetical protein 113 Peptidase-M56 domain-containing protein WP_005778093.1 UPI0001B49925 UniRef100_A0A081U172,UniRef50_A0A0I9URT8,UniRef90_A0A0I9URT8

group_15386 1 81 -80 4.394449155 0 -4.394449155 hypothetical protein;Domain of unknown function;DUF4988 domain-containing protein;PL29 family lyase N-terminal domain-containing protein;The GLUG motif 112 PL29 family lyase N-terminal domain-containing protein WP_188520949.1 UPI001666E6A4 UniRef100_UPI001666E6A4,UniRef50_A0A4P8M120,UniRef90_A0A4P8M120

group_1753 1 81 -80 4.394449155 0 -4.394449155 hypothetical protein;DUF4440 domain-containing protein 112 hypothetical protein

group_15011 1 80.28571429 -79.28571429 4.385591701 0 -4.385591701 hypothetical protein 111 hypothetical protein WP_005812506.1 UPI0002808A8F UniRef100_A0A081TUJ2,UniRef50_A0A0I9S6K4,UniRef90_A0A0I9S6K4

group_15010 1 80.28571429 -79.28571429 4.385591701 0 -4.385591701 toxin-antitoxin system YwqK family antitoxin 111 toxin-antitoxin system YwqK family antitoxin WP_005782880.1 UPI0001B4AF30 UniRef100_A0A0I9RR43,UniRef50_E1WW01,UniRef90_A0A0I9RR43

group_15009 1 80.28571429 -79.28571429 4.385591701 0 -4.385591701 DUF5991 domain-containing protein;hypothetical protein 111 DUF5991 domain-containing protein WP_005782878.1 UPI0001B4AF2F UniRef100_A0A2K9H2S6,UniRef50_A0A1Q6GD02,UniRef90_A0A396BZE6

group_7513 1 80.28571429 -79.28571429 4.385591701 0 -4.385591701 hypothetical protein 111 hypothetical protein UniRef50_A0A4P8LYS6,UniRef90_A0A4P8LYS6

group_5009 1 80.28571429 -79.28571429 4.385591701 0 -4.385591701 hypothetical protein 111 hypothetical protein

group_4075 2.083032491 76 -73.91696751 4.33073334 0.73382476 -3.59690858 hypothetical protein 111 hypothetical protein

group_15763 1 79.57142857 -78.57142857 4.376655091 0 -4.376655091 hypothetical protein 110 hypothetical protein

group_10922 3.527075812 69.57142857 -66.04435276 4.242353974 1.260469146 -2.981884828 hypothetical protein 110 hypothetical protein UniRef50_I9V5W7,UniRef90_I9V5W7

group_10013 1.180505415 78.14285714 -76.96235173 4.358538653 0.165942665 -4.192595989 hypothetical protein 110 hypothetical protein UPI0012309DFF UniRef100_A0A5M5PX17,UniRef50_A0A5M5PX17,UniRef90_A0A5M5PX17

group_7106 1 79.57142857 -78.57142857 4.376655091 0 -4.376655091 site-specific integrase;Tyr recombinase domain-containing protein;hypothetical protein;recombinase 110 Tyr recombinase domain-containing protein UniRef50_A0A0F5JTI0

group_6261 1 79.57142857 -78.57142857 4.376655091 0 -4.376655091 glycosyltransferase family 4 protein 110 glycosyltransferase family 4 protein UniRef50_A0A174SJV0,UniRef90_A0A0I9RV27

group_1707 1 79.57142857 -78.57142857 4.376655091 0 -4.376655091 glycosyltransferase 110 glycosyltransferase WP_005776895.1 UPI0001B494DB UniRef100_A0A7D4KAS0,UniRef50_A0A174IM66,UniRef90_A0A081TXL9

group_9337 1 78.85714286 -77.85714286 4.367637897 0 -4.367637897 hypothetical protein 109 hypothetical protein UniRef50_R5S3Q0,UniRef90_R5S3Q0

group_8144 1 78.85714286 -77.85714286 4.367637897 0 -4.367637897 hypothetical protein 109 hypothetical protein UniRef50_A0A0F5IP92,UniRef90_UPI001896EAFA

group_5982 3.346570397 69.57142857 -66.22485817 4.242353974 1.207936059 -3.034417915 hypothetical protein 109 hypothetical protein WP_193684586.1 UPI001879E992 UniRef100_UPI001879E992,UniRef50_I9KFS0,UniRef90_I9KFS0

group_4428 1 78.85714286 -77.85714286 4.367637897 0 -4.367637897 family 20 glycosylhydrolase;hypothetical protein;beta-N-acetylhexosaminidase 109 beta-N-acetylhexosaminidase WP_005778287.1 UPI0001B49D9F UniRef100_A0A2K9H0E4,UniRef50_A0A0I9RKP0,UniRef90_A0A0I9RKP0

group_9759 1.36101083 76 -74.63898917 4.33073334 0.308227681 -4.022505659 hypothetical protein 108 hypothetical protein UniRef50_A0A1Y4J3W7,UniRef90_A0A0I9SDK0

group_9633 3.346570397 68.85714286 -65.51057246 4.232033965 1.207936059 -3.024097906 hypothetical protein 108 hypothetical protein WP_022012974.1 UPI000334B0C6 UniRef100_R5RFW0,UniRef50_I9VSM2,UniRef90_R5RFW0

group_6138 1 78.14285714 -77.14285714 4.358538653 0 -4.358538653 AraC family transcriptional regulator;helix-turn-helix domain-containing protein 108 helix-turn-helix domain-containing protein WP_227559639.1 UPI001D0EECD2 UniRef100_UPI001D0EECD2,UniRef50_A0A5E8K011,UniRef90_UPI000317D5D6

group_19352 1 77.42857143 -76.42857143 4.349355852 0 -4.349355852 hypothetical protein 107 hypothetical protein

group_10305 1 77.42857143 -76.42857143 4.349355852 0 -4.349355852 hypothetical protein 107 hypothetical protein UniRef50_D6D4Q0,UniRef90_D6D4Q0

group_9032 1 77.42857143 -76.42857143 4.349355852 0 -4.349355852 hypothetical protein 107 hypothetical protein UniRef50_A0A0P0ETS8,UniRef90_A0A2K9HD30

group_8514 1 77.42857143 -76.42857143 4.349355852 0 -4.349355852 hypothetical protein 107 hypothetical protein WP_220575438.1 UPI001C72F18E UniRef100_UPI001C72F18E,UniRef50_A0A0I9RRT2,UniRef90_A0A0I9RRT2

group_6664 1 77.42857143 -76.42857143 4.349355852 0 -4.349355852 EamA domain-containing protein 107 EamA domain-containing protein WP_220653657.1 UPI001C7D2D66 UniRef100_UPI001C7D2D66,UniRef50_A0A412XW58,UniRef90_A0A412XW58

group_5303 1 77.42857143 -76.42857143 4.349355852 0 -4.349355852 Putative OmpA-OmpF-like porin family 107 Putative OmpA-OmpF-like porin family WP_122143556.1 UPI000ED66F04 UniRef100_A0A412XS09,UniRef50_A0A081UFV4,UniRef90_A0A0I9SCV8

group_3156 1 77.42857143 -76.42857143 4.349355852 0 -4.349355852 DUF5009 domain-containing protein;hypothetical protein 107 DUF5009 domain-containing protein WP_005775597.1 UPI0001EC3E88 UniRef100_A0A081U2V5,UniRef50_K5Z707,UniRef90_A0A081U2V5

group_2155 1 77.42857143 -76.42857143 4.349355852 0 -4.349355852 DUF4369 domain-containing protein;hypothetical protein 107 DUF4369 domain-containing protein UPI0002808E67 UniRef100_A0A3E5CXW1,UniRef50_A0A0E2AVS1,UniRef90_A0A3E5CXW1

group_1418 1 77.42857143 -76.42857143 4.349355852 0 -4.349355852 hypothetical protein 107 hypothetical protein UniRef50_A0A081U5D5,UniRef90_A0A081U5D5

group_6959 1 76.71428571 -75.71428571 4.340087945 0 -4.340087945 hypothetical protein;Core-binding (CB) domain-containing protein;Integrase 106 Integrase UniRef50_R9IDU4,UniRef90_A0A081U355

group_6270 1 76.71428571 -75.71428571 4.340087945 0 -4.340087945 hypothetical protein 106 hypothetical protein UPI0002808B1F UniRef100_R5RF23,UniRef50_R5RF23,UniRef90_R5RF23

group_10380 2.263537906 71 -68.73646209 4.262679877 0.816929034 -3.445750843 hypothetical protein 105 hypothetical protein WP_220653815.1 UPI001C7DDF3E UniRef100_UPI001C7DDF3E,UniRef50_R5RC33,UniRef90_UPI001C7DDF3E

group_7196 1 76 -75 4.33073334 0 -4.33073334 hypothetical protein;GyrI-like domain-containing protein;AraC family transcriptional regulator 105 AraC family transcriptional regulator WP_032530651.1 UPI0004D96FFB UniRef100_A0A4P8LSK3,UniRef50_A0A0P0FKX7,UniRef90_A0A0I9S253

group_5921 1 74.57142857 -73.57142857 4.311757439 0 -4.311757439 hypothetical protein 103 hypothetical protein UniRef50_R5RAP6,UniRef90_R5RAP6

group_1504 1 74.57142857 -73.57142857 4.311757439 0 -4.311757439 hypothetical protein 103 hypothetical protein UniRef50_R5R817,UniRef90_R5R817

group_10452 1 73.85714286 -72.85714286 4.302132725 0 -4.302132725 DUF4469 domain-containing protein 102 DUF4469 domain-containing protein WP_220653738.1 UPI001C7CB7E7 UniRef100_UPI001C7CB7E7,UniRef50_R5REL4,UniRef90_A0A0I9RTX4

group_1857 1.722021661 71 -69.27797834 4.262679877 0.543498985 -3.719180892 hypothetical protein 102 hypothetical protein

group_5099 1 73.14285714 -72.14285714 4.292414476 0 -4.292414476 hypothetical protein;DUF4906 domain-containing protein 101 DUF4906 domain-containing protein WP_005780023.1 UPI0001B4914F UniRef100_A0A2K9GYZ9,UniRef50_A0A081TS68,UniRef90_A0A081TS68

group_1188 1 73.14285714 -72.14285714 4.292414476 0 -4.292414476 ABC transporter ATP-binding protein 101 ABC transporter ATP-binding protein WP_032530042.1 UPI0004D82349 UniRef100_A0A4P8LTV4,UniRef50_A0A174SKS5,UniRef90_A0A396BWX5

group_570 1 73.14285714 -72.14285714 4.292414476 0 -4.292414476 ABC transporter ATP-binding protein 101 ABC transporter ATP-binding protein WP_195317223.1 UPI00189B1394 UniRef100_UPI00189B1394,UniRef50_A0A174SKA1,UniRef90_A0A396C3B1

group_7275 1.180505415 71.71428571 -70.5337803 4.272689971 0.165942665 -4.106747306 hypothetical protein 100 hypothetical protein UniRef50_R5RH71,UniRef90_R5RH71

group_1912 1.180505415 71 -69.81949458 4.262679877 0.165942665 -4.096737212 hypothetical protein 99 hypothetical protein UniRef50_Q64PF7,UniRef90_A0A396BLU1

group_4690 1 71 -70 4.262679877 0 -4.262679877 hypothetical protein;BF3164 family lipoprotein 98 hypothetical protein WP_032542407.1 UPI0005165AD8 UniRef100_A0A5E8KNC2,UniRef50_U6RNP1,UniRef90_A0A0I9USE5

group_3534 1 71 -70 4.262679877 0 -4.262679877 aminoglycoside phosphotransferase 98 aminoglycoside phosphotransferase WP_122130224.1 UPI000EFCC333 UniRef100_A0A413K391,UniRef50_A0A0K6BQD8,UniRef90_A0A0K6BQD8

group_18352 1 68.85714286 -67.85714286 4.232033965 0 -4.232033965 twin-arginine translocase TatA/TatE family subunit 95 twin-arginine translocase TatA/TatE family subunit WP_005775616.1 UPI0001B48EB5 UniRef100_A0A081U2W9,UniRef50_G5SRN7,UniRef90_A0A0I9SB40

group_7088 1 68.85714286 -67.85714286 4.232033965 0 -4.232033965 twin-arginine translocase subunit TatC 95 twin-arginine translocase subunit TatC WP_005775615.1 UPI0001B48EB4 UniRef100_A0A081TVT0,UniRef50_F8WWF2,UniRef90_A0A2M9VAL5

group_1909 1 68.85714286 -67.85714286 4.232033965 0 -4.232033965 hypothetical protein 95 hypothetical protein UniRef50_R5RHU6,UniRef90_R5RHU6

group_18920 1 68.14285714 -67.14285714 4.221606342 0 -4.221606342 hypothetical protein 94 hypothetical protein UPI0002808BB8 UniRef100_R5S109,UniRef50_A0A015V927,UniRef90_R5S109

group_15036 2.083032491 63.85714286 -61.77411037 4.156648446 0.73382476 -3.422823685 hypothetical protein;Transposase family protein;transposase family protein 94 hypothetical protein UniRef50_B0NNR1,UniRef90_A0A1E9BV01

group_5818 1 68.14285714 -67.14285714 4.221606342 0 -4.221606342 Single-stranded DNA-binding protein;single-stranded DNA-binding protein 94 Single-stranded DNA-binding protein UniRef50_Q8A7M7

gldE 1 68.14285714 -67.14285714 4.221606342 0 -4.221606342 hemolysin 94 hemolysin UniRef50_E6K7H2,UniRef90_A0A2M9VAE6

group_1037 1 68.14285714 -67.14285714 4.221606342 0 -4.221606342 4'-phosphopantetheinyl transferase superfamily protein 94 4'-phosphopantetheinyl transferase superfamily protein WP_220654690.1 UPI001C7CE7DD UniRef100_UPI001C7CE7DD,UniRef50_A0A5J4SL81,UniRef90_A0A081U0Y8

group_713 1 68.14285714 -67.14285714 4.221606342 0 -4.221606342 arylsulfatase 94 arylsulfatase WP_032540016.1 UPI0001B496AC UniRef100_A0A4P8LDK7,UniRef50_A0A174NVF4,UniRef90_Q5LF23

group_15007 1.36101083 66 -64.63898917 4.189654742 0.308227681 -3.881427061 hypothetical protein 93 hypothetical protein

group_12237 1 67.42857143 -66.42857143 4.211068837 0 -4.211068837 DUF1016 domain-containing protein;PDDEXK nuclease domain-containing protein 93 DUF1016 domain-containing protein WP_005821283.1 UPI0002824F00 UniRef100_A0A0I9S6T3,UniRef50_A0A240EC11,UniRef90_A6L8M1

group_1073 1 67.42857143 -66.42857143 4.211068837 0 -4.211068837 hypothetical protein 93 hypothetical protein

group_4739 1 66.71428571 -65.71428571 4.200419109 0 -4.200419109 xylosidase 92 xylosidase WP_005778601.1 UPI0001B4A840 UniRef100_A0A7D4GCY9,UniRef50_I9Q3M3,UniRef90_A0A0I9S4G0

group_6775 1 66 -65 4.189654742 0 -4.189654742 hypothetical protein 91 hypothetical protein UniRef50_A0A0P0F9E0,UniRef90_D6D4P4

group_5630 1 66 -65 4.189654742 0 -4.189654742 hypothetical protein 91 hypothetical protein

group_7582 1 65.28571429 -64.28571429 4.178773242 0 -4.178773242 DNA-binding response regulator 90 DNA-binding response regulator WP_005778313.1 UPI0001B49F81 UniRef100_A0A7D4GA67,UniRef50_A0A0P0F0V3,UniRef90_A0A081TYA5

group_6060 1.180505415 64.57142857 -63.39092316 4.167772031 0.165942665 -4.001829366 hypothetical protein 90 hypothetical protein

group_4619 1 65.28571429 -64.28571429 4.178773242 0 -4.178773242 SF4 helicase domain-containing protein 90 SF4 helicase domain-containing protein UniRef50_A0A0I9S8B8,UniRef90_A0A0I9S8B8

group_4174 1 65.28571429 -64.28571429 4.178773242 0 -4.178773242 hypothetical protein;histidine kinase 90 histidine kinase WP_005778311.1 UPI0001B49F80 UniRef100_A0A081TYA6,UniRef50_A0A0P0FFU5,UniRef90_A0A081TYA6

group_2365 1 65.28571429 -64.28571429 4.178773242 0 -4.178773242 hypothetical protein;DUF4251 domain-containing protein 90 DUF4251 domain-containing protein WP_022012619.1 UPI00033E09A6 UniRef100_A0A0I9SAD9,UniRef50_F0R322,UniRef90_A0A0I9SAD9

group_15033 1 64.57142857 -63.57142857 4.167772031 0 -4.167772031 hypothetical protein 89 hypothetical protein UPI0002808626 UniRef100_R5S401,UniRef50_R5S401,UniRef90_R5S401

group_14387 1 64.57142857 -63.57142857 4.167772031 0 -4.167772031 hypothetical protein 89 hypothetical protein

group_11522 2.624548736 58.14285714 -55.51830841 4.062903036 0.964908971 -3.097994065 hypothetical protein;6-bladed beta-propeller 89 6-bladed beta-propeller UniRef50_R5RVU1,UniRef90_R5RVU1

group_6946 1 63.85714286 -62.85714286 4.156648446 0 -4.156648446 hypothetical protein 88 hypothetical protein

group_5127 1.180505415 63.14285714 -61.96235173 4.145399733 0.165942665 -3.979457068 hypothetical protein 88 hypothetical protein WP_005777993.1 UPI0001B49510 UniRef100_A0A081UDW3,UniRef50_A0A0I9SAB1,UniRef90_A0A0I9SAB1

group_4087 1 63.85714286 -62.85714286 4.156648446 0 -4.156648446 hypothetical protein;DUF421 domain-containing protein;Transmembrane protein YetF 88 DUF421 domain-containing protein UniRef50_A0A379MQ58,UniRef90_A0A2K9GXT5

group_8010 1 63.14285714 -62.14285714 4.145399733 0 -4.145399733 hypothetical protein 87 hypothetical protein UniRef50_R5R839,UniRef90_R5R839

group_6905 1 63.14285714 -62.14285714 4.145399733 0 -4.145399733 hypothetical protein 87 hypothetical protein

group_11317 2.444043321 56.71428571 -54.27024239 4.038026132 0.893653767 -3.144372365 NVEALA domain-containing protein;NVEALA protein 86 NVEALA domain-containing protein WP_042985692.1 UPI0004B89EF9 UniRef100_UPI0004B89EF9,UniRef50_A0A7D4FWH4,UniRef90_UPI0004B89EF9

group_12242 1.36101083 60.28571429 -58.92470346 4.099095165 0.308227681 -3.790867484 hypothetical protein;6-bladed beta-propeller;BF3164 family lipoprotein 85 hypothetical protein UniRef50_Q5LI55,UniRef90_A0A853Q1L0

group_1101 1 61.71428571 -60.71428571 4.122515439 0 -4.122515439 hypothetical protein 85 hypothetical protein UniRef50_A0A413JTP3,UniRef90_A0A413JTP3

group_19393 1 61 -60 4.110873864 0 -4.110873864 hypothetical protein 84 hypothetical protein

group_10260 1.36101083 59.57142857 -58.21041774 4.087176073 0.308227681 -3.778948391 hypothetical protein 84 hypothetical protein

group_7383 1 61 -60 4.110873864 0 -4.110873864 hypothetical protein 84 hypothetical protein

group_12327 1 60.28571429 -59.28571429 4.099095165 0 -4.099095165 hypothetical protein 83 hypothetical protein WP_182007626.1 UPI0015F559CC UniRef100_A0A412Y369,UniRef50_A0A4P8LD89,UniRef90_A0A4P8LD89

group_2919 1 60.28571429 -59.28571429 4.099095165 0 -4.099095165 hypothetical protein 83 hypothetical protein WP_005779621.1 UPI0001EC41BA UniRef100_A0A0I9SDK4,UniRef50_A0A0K6BPI0,UniRef90_A0A0I9SDK4

group_8691 1.180505415 58.85714286 -57.67663744 4.0751132 0.165942665 -3.909170536 hypothetical protein 82 hypothetical protein UniRef50_UPI001C731804,UniRef90_UPI001C731804

group_15946 1 57.42857143 -56.42857143 4.05054194 0 -4.05054194 hypothetical protein 79 hypothetical protein

group_4567 1 57.42857143 -56.42857143 4.05054194 0 -4.05054194 hypothetical protein 79 hypothetical protein UniRef50_A0A081TUJ1,UniRef90_A0A081TUJ1

group_2677 1 57.42857143 -56.42857143 4.05054194 0 -4.05054194 flavodoxin 79 flavodoxin WP_005805928.1 UPI0002808835 UniRef100_A0A3E5CV02,UniRef50_I9S3Q4,UniRef90_I9S3Q4

group_15294 1 56.71428571 -55.71428571 4.038026132 0 -4.038026132 hypothetical protein 78 hypothetical protein UPI0002824E03 UniRef100_A0A396BVM4,UniRef50_A0A396BVM4,UniRef90_A0A396BVM4

group_6460 1.541516245 54.57142857 -53.02991233 3.99951046 0.432766507 -3.566743953 hypothetical protein 78 hypothetical protein

group_16120 1.180505415 54.57142857 -53.39092316 3.99951046 0.165942665 -3.833567795 hypothetical protein 77 hypothetical protein WP_005781462.1 UPI0001B4A426 UniRef100_A0A081U8G8,UniRef50_A0A0J9FIR4,UniRef90_A0A081U8G8

group_5102 1 55.28571429 -54.28571429 4.012514544 0 -4.012514544 DUF1016 domain-containing protein;Putative nuclease YhcG;hypothetical protein 76 DUF1016 domain-containing protein UniRef50_A0A1H3XU25,UniRef90_A0A2K9H576

group_2968 1 55.28571429 -54.28571429 4.012514544 0 -4.012514544 hypothetical protein 76 hypothetical protein WP_032536163.1 UPI0005178755 UniRef100_A0A0I9RUA8,UniRef50_A0A0I9RUA8,UniRef90_A0A0I9RUA8

group_1874 1 55.28571429 -54.28571429 4.012514544 0 -4.012514544 hypothetical protein 76 hypothetical protein UniRef50_A0A081TXM2,UniRef90_A0A081TXM2

group_1163 1 55.28571429 -54.28571429 4.012514544 0 -4.012514544 hypothetical protein 76 hypothetical protein UPI000EBD5156 UniRef100_A0A396BWC8,UniRef50_A0A2K9H6G6,UniRef90_A0A2K9H6G6

group_17046 1 54.57142857 -53.57142857 3.99951046 0 -3.99951046 GxxExxY protein 75 GxxExxY protein WP_005778635.1 UPI0001B4A852 UniRef100_A0A0I9TLK9,UniRef50_A0A6M0CRB9,UniRef90_A0A0I9TLK9

group_16431 1 54.57142857 -53.57142857 3.99951046 0 -3.99951046 DUF6108 domain-containing protein 75 DUF6108 domain-containing protein WP_005809082.1 UPI00028085E5 UniRef100_A0A081TME4,UniRef50_A0A3L7ZQY1,UniRef90_A0A0I9RLZ1

group_2734 1 54.57142857 -53.57142857 3.99951046 0 -3.99951046 hypothetical protein 75 hypothetical protein UniRef50_A0A3E5IIS8,UniRef90_A0A4P8LEA5

group_15006 1 53.85714286 -52.85714286 3.986335038 0 -3.986335038 hypothetical protein 74 hypothetical protein UPI0002809674 UniRef100_R5RJF5,UniRef50_R5RJF5,UniRef90_R5RJF5

group_6921 1 53.85714286 -52.85714286 3.986335038 0 -3.986335038 hypothetical protein 74 hypothetical protein UPI00028092EA UniRef100_A0A0I9S388,UniRef50_A0A0I9S388,UniRef90_A0A0I9S388

group_4849 1 53.85714286 -52.85714286 3.986335038 0 -3.986335038 OMP-b-brl-2 domain-containing protein 74 OMP-b-brl-2 domain-containing protein WP_122142385.1 UPI000EFC1EA9 UniRef100_A0A412YCH2,UniRef50_A0A015TWG3,UniRef90_A0A015TWG3

group_15083 1 52.42857143 -51.42857143 3.959451699 0 -3.959451699 Transposase;DDE transposase;Transposase family protein;DDE-Tnp-ISL3 domain-containing protein;hypothetical protein 72 DDE-Tnp-ISL3 domain-containing protein UniRef50_K6A0D8,UniRef90_I9PRF7

group_4729 1 52.42857143 -51.42857143 3.959451699 0 -3.959451699 hypothetical protein 72 hypothetical protein UniRef50_R5R9W9,UniRef90_R5R9W9

group_3418 1 52.42857143 -51.42857143 3.959451699 0 -3.959451699 hypothetical protein 72 hypothetical protein

group_3181 1.180505415 51.71428571 -50.5337803 3.945734063 0.165942665 -3.779791398 addiction module toxin RelE;Addiction module toxin RelE 72 addiction module toxin RelE UniRef50_A0A357Z7A8,UniRef90_A0A0I9SBW5

tadA 1 52.42857143 -51.42857143 3.959451699 0 -3.959451699 tRNA-specific adenosine deaminase 72 tRNA-specific adenosine deaminase WP_005809086.1 UPI00028085E7 UniRef100_A0A081U5C5,UniRef50_O34598,UniRef90_A0A0P0FBN9

group_6852 1 51.71428571 -50.71428571 3.945734063 0 -3.945734063 hypothetical protein 71 hypothetical protein UPI000280903D UniRef100_R5RK78,UniRef50_R5RK78,UniRef90_R5RK78

group_6284 1 51.71428571 -50.71428571 3.945734063 0 -3.945734063 DegT/DnrJ/EryC1/StrS family aminotransferase;pyridoxal phosphate-dependent aminotransferase;Aminotransferase class I/II-fold pyridoxal phosphate-dependent enzyme 71 pyridoxal phosphate-dependent aminotransferase WP_122296203.1 UPI000EFE5BE8 UniRef100_UPI000EFE5BE8,UniRef50_A0A174F6S5,UniRef90_Q5LH87

group_6267 1 51.71428571 -50.71428571 3.945734063 0 -3.945734063 hypothetical protein 71 hypothetical protein

group_1782 1 51.71428571 -50.71428571 3.945734063 0 -3.945734063 ATP-binding protein 71 ATP-binding protein WP_005775543.1 UPI0001B4AC31 UniRef100_A0A0I9RQU1,UniRef50_F0R6X9,UniRef90_A0A0I9RQU1

lpxK 1 51 -50 3.931825633 0 -3.931825633 tetraacyldisaccharide 4'-kinase 70 tetraacyldisaccharide 4'-kinase WP_005821381.1 UPI00028252B1 UniRef100_R5RKS4,UniRef50_A6L0S5,UniRef90_A0A396BM63

sppA 1 51 -50 3.931825633 0 -3.931825633 hypothetical protein;signal peptide peptidase SppA 70 signal peptide peptidase SppA WP_005807878.1 UPI0002808B47 UniRef100_A0A3E5CCL2,UniRef50_G8UNC2,UniRef90_A0A2M9VC11

Table 3: Genes core to either division I or II with annotation.
